# How Much Can Product Reformulation Improve Diet Quality in Households with Children and Adolescents?

**DOI:** 10.3390/nu11030618

**Published:** 2019-03-14

**Authors:** Mary K. Muth, Shawn A. Karns, Lisa Mancino, Jessica E. Todd

**Affiliations:** 1 RTI International, Research Triangle Park, NC 27709, USA; karns@rti.org; 2 Economic Research Service, U.S. Department of Agriculture, Washington, DC 20024, USA; lmancino@ers.usda.gov (L.M.); jtodd@ers.usda.gov (J.E.T.)

**Keywords:** food reformulation, scanner data, diet quality, children, adolescents

## Abstract

Improvements in the healthfulness of packaged foods and beverages through reformulation could help reduce the prevalence of obesity among children and adolescents through improved diet quality. This study assessed changes in calories and four nutrients (saturated fat, total sugars, sodium, and dietary fiber) from 2012 through 2014 for packaged products frequently consumed by children and adolescents, simulated effects of potential improvements in 12 frequently consumed product categories based on actual purchasing patterns, and compared differences in prices of healthier versus less healthy products. Analysis of trends showed limited evidence that healthfulness of foods improved over the years examined. Simulation results showed minimal changes for calories and sodium, but daily intake of saturated fat could decrease by 4%, sugar consumption could decrease by 5%, and dietary fiber consumption could increase by 11% if products were reformulated to meet an existing healthfulness standard. Using a higher standard, caloric intake could decline by 4%, saturated fat by 6%, sugar by 9%, and sodium by 4%, and dietary fiber could increase by 14%. Healthier versions of most products ranged from an average of 3 to 12 cents more per serving, but not all healthier versions were more costly. Overall, reformulation is a potential avenue for improving diet quality in households with children and adolescents, but price could be a barrier to purchasing healthier products for some households.

## 1. Introduction

Although the rate of increase slowed in recent years, the prevalence of obesity among children and adolescents in the United States (US) remains high at 18.5% in 2015–2016 [1]. Rates are highest for adolescents (20.6%), followed by six- to 11-year-olds (18.4%), and finally two- to five-year-olds (13.9%) and are higher for Hispanics (25.8%) and non-Hispanic blacks (22.0%) compared with other populations [1]. The public health community is concerned about the prevalence of obesity in children and adolescents and its long-term health effects, and the nutritional quality of the food supply is one of several potential contributors to the problem. Packaged foods and sugar-sweetened beverages are a substantial portion of calories consumed by children and adolescents; some of these foods are consumed as part of a healthy meal, and many others are consumed as snacks or sweets. Improvements in the healthfulness of packaged foods through reformulation could have an impact on child and adolescent obesity through improved dietary quality [2,3,4].

Although food manufacturers might reformulate foods for a number of different reasons, such as to reduce production costs or improve sensory characteristics, we focus here on reformulation to improve healthfulness. The effect of reformulation on child and adolescent obesity can occur even if households do not alter their food purchasing behavior; thus, it offers a different mechanism to affect childhood obesity and can complement efforts to change food choice behavior. Reformulation of foods to improve healthfulness can help remove the barriers to access to healthy foods for all populations, including rural, low-income, and ethnic and racial minority populations. Reformulation affects all consumers, not only those who are nutritionally aware [5]. Some governments began focusing on reformulation as one method to reduce childhood obesity. For example, the United Kingdom’s plan of action for childhood obesity released in 2016 calls for reductions in sugar, which should be accompanied by a reduction in calories, and the plan is conducted alongside work to achieve salt reduction targets [6]. Also, the Food and Drink Industry Ireland, under the oversight of the Food Safety Authority of Ireland, launched a national food reformulation program as part of an effort to tackle obesity [7].

Food manufacturers, as profit-seeking enterprises, consider the cost of reformulation, which is estimated to range from $5000 to over $4,000,000 per product [8], and the potential effect of reformulation on consumer acceptability and, thus, product sales. However, food manufacturers respond to other external incentives and already engage in voluntary efforts to improve the healthfulness of food and beverage products through reformulation. In particular, recent initiatives include the Healthy Weight Commitment by 16 major food manufacturers, covering an estimated 36% of calories in the marketplace [9,10], the Balance Calories Initiative by three major beverage manufacturers, and sodium reduction pledges in several countries. A verification study for the Healthy Weight Commitment initiative in the United States found that food manufacturers adhered to a commitment to reduce calories in packaged foods, but the study did not consider saturated fat, sugar, sodium, and fiber [9,10,11,12,13]. In contrast, a more recent evaluation study analyzed changes in placement, promotion, and prices of different types of beverages to determine whether the Balance Calories Initiative might result in reduced calories in beverage purchases in the United States, but found little evidence of changes [14]. Several other studies tracked sodium reduction pledges in the United States and internationally, and found that food manufacturers reformulated foods to reduce sodium [15,16,17,18,19].

New initiatives that were implemented recently could further encourage reformulation. For example, the Access to Nutrition Foundation (ATNF), an international organization funded by the Bill and Melinda Gates Foundation, Robert Wood Johnson Foundation, and the government of the Netherlands, released product profiles that assess the healthfulness of products produced by 10 major food manufacturers in the United States [20]. The ATNF report calls on manufacturers to invest more in increasing the nutritional quality of their products and adopt a clear definition of healthy products. Also, the Children’s Food and Beverage Advertising Initiative (CFBAI) is a voluntary initiative administered by the Better Business Bureau in the United States in which companies commit to advertising to children under age 12 only foods that meet its nutrition criteria. CFBAI recently released new nutrition criteria and estimates that approximately 40% of foods on its current list will need to be reformulated to meet the new criteria before 1 January 2020 (the implementation date) [21].

The food industry also has new incentives to reformulate foods in response to upcoming changes in the nutrition facts label and serving sizes that were published on 27 May 2016, and will go into effect for most manufacturers on 1 July 2020 [22]. As evidenced by past labeling changes, food manufacturers respond to changes in labeling requirements by reformulating foods. In particular, after labeling requirements for trans fatty acids went into effect on 1 January 2006, food manufacturers undertook substantial reformulation of foods to reduce or remove trans fatty acids from packaged foods [23,24,25,26,27,28]. Furthermore, food manufacturers reformulated foods in response to changes in recommendations for consumption of whole grains [29]. The expectation is that substantial reformulation may occur in response to the new requirements, such as declaring added sugars and an associated percent daily value, changing the definition of dietary fiber, updating daily reference values and reference daily intakes, including establishing values for young children, increasing the prominence of calories, and updating serving sizes for many foods. The Food and Drug Administration’s (FDA’s) final regulatory impact analysis for the nutrition facts label and serving size rules notes that the rule will motivate reformulation of foods, thus resulting in public health benefits.

The overall goal of this study was to determine the extent to which reformulation of foods frequently consumed by children and adolescents could improve the healthfulness of available foods and diet quality. We focused the analysis on per-serving values for calories, saturated fat, total sugars, sodium, and dietary fiber. Although sodium is not directly associated with weight status, we included it in the analysis because manufacturers may be balancing taste profiles of foods across fat, sugar, and salt. In other words, when manufacturers reformulate foods, they make trade-offs in taste and other sensory characteristics.

Our specific objectives were to (1) test whether healthfulness of foods frequently consumed by children improved over the 2012 through 2014 time period (prior to publication of the new nutrition facts label rule), (2) estimate the extent to which reformulation of foods to increase healthfulness could improve diet quality based on current purchasing patterns of households with children and adolescents, and (3) because reformulation could raise the costs of manufacturing foods, assess whether healthier foods have higher average prices than less healthy foods. We considered differences across demographic categories of households (race and ethnicity, income, and urban/rural location) in assessing the potential effects of reformulation. We also considered differences across branded and private-label products in assessing differences in prices of healthier versus less healthy foods.

## 2. Materials and Methods

We developed our methods based on a conceptual model of the effects of reformulation on child and adolescent weight status as described below. Data resources included IRI store scanner data, household scanner data, and nutrition label data, in conjunction with publicly available dietary recall and nutrition criteria data. In this section, we describe the analytical methods applied to these data.

### 2.1. Study Design

#### 2.1.1. Conceptual Model

**Figure 1:**
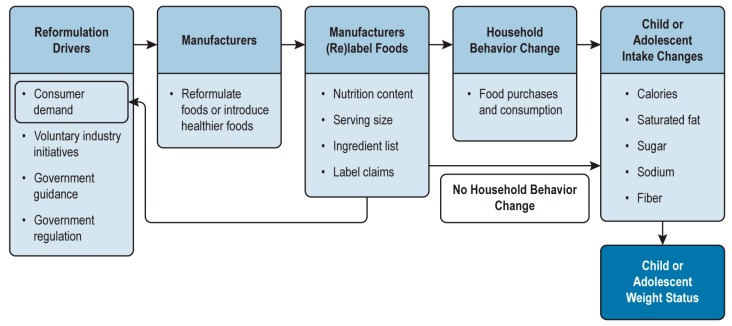
Conceptual model of the effects of food reformulation on child and adolescent obesity.

Figure 1 shows a conceptual model of reformulation starting with several drivers originating from consumers, industry initiatives, and government. Reformulation drivers include changes in consumer knowledge and attitudes that cause an increase in demand for healthier foods, voluntary industry initiatives under which food companies commit to offering and promoting healthier foods, government guidance that encourages offering healthier foods, and government regulations (e.g., changes to the nutrition facts label and standard serving sizes and banning of partially hydrogenated oils as a food ingredient). Food manufacturers reformulate foods or introduce healthier versions of foods in response to these drivers and subsequently relabel the foods to align with their attributes. Note that the desire to reformulate may be driven by the desire to include product claims or other label information, in which case the direction of causality could be reversed.

The relabeling of foods or the introduction of new foods could affect household food purchasing and consumption behaviors and, thus, affect intake of calories, saturated fat, sugar, sodium, and fiber by children and adolescents. Many individuals do not read or understand label information and, therefore, are unlikely to change behavior in response to relabeling [30,31]. However, even if households do not change their food purchasing behavior in response to label information, diet quality can improve as a result of reformulation, assuming that potential changes in sensory characteristics or prices of foods do not alter consumption patterns. Therefore, if food manufacturers are motivated to reformulate foods to improve healthfulness, reformulation should result in improved weight status in children and adolescents over time.

In this conceptual model, we assumed that food manufacturers do not increase the prices of foods to recoup the costs of reformulation. If, however, prices increase, purchases of healthier foods may decline, thus dampening the potential benefits associated with the availability of healthier foods in the marketplace.

#### 2.1.2. Data Sources and Preparation

To conduct the analysis, we used government survey data on dietary intake, commercial store and household scanner data matched to nutrition data, and nutrition criteria from rating systems developed by nongovernmental organizations. We describe our analyses of data from each source in more detail below.

To select product categories, we focused on packaged products frequently consumed by children and adolescents that can be reformulated. Products like milk, fruits, and vegetables have no or few options for reformulation and were, therefore, excluded. We also focused on products primarily consumed as-packaged, because mixes (e.g., pancake mixes and rice side dish mixes) can be prepared in different ways that affect the nutrient levels of the final food. Based on the first day of 24-h National Health and Nutrition Examination Survey (NHANES) dietary recall data for children and adolescents for 2013–2014, we identified 17 packaged products that were top sources of calories for one or more age groups across children and adolescents. These products included cereal (granola) bars, cookies and brownies, crackers, frankfurters, fruit drinks, ice cream, lunch combinations, macaroni and cheese mix, ready-to-eat (RTE) macaroni and cheese, nut butters, pizza, potato chips, RTE cereal, soft drinks, tortilla and other chips, yeast breads, and yogurt. All 17 product categories were used to calculate changes in average nutrient levels over time. To simulate potential changes due to reformulation, we focused the analysis on the 12 product categories that had five or more products with at least one star in the Guiding Stars program. Frankfurters, lunch combinations, nut butters, soft drinks, and RTE macaroni and cheese had no or too few products that had at least one star. Finally, six product categories (cereal bars, crackers, tortilla and other chips, RTE cereal, yeast bread, and yogurt) contained foods meeting the CFBAI criteria for child-directed adverting and could, therefore, be used to calculate differences in prices of healthier versus less healthy foods. Macaroni and cheese mix, pizza, and potato chips each had a few products meeting the CFBAI criteria, but they comprised less than 1% of products.

We used IRI InfoScan store scanner data matched with nutrition data at the barcode level to track changes in nutrients over time for the years 2012 through 2014 or 2015. InfoScan data represent barcode-level purchase data for approximately 60,000 grocery, drug, mass merchandiser, convenience, and dollar stores across the United States [32]. These data are provided directly by stores to IRI and tend to cover all the major retail chains and some smaller stores in the United States consistently across years. We included both branded and private-label products in the analysis, because branded manufacturers may have different production and marketing processes than retailers that produce their own private-label products. Private-label products comprise an estimated 15% to 17% of total foods sales [33] and, thus, a substantial portion of food consumption in the United States.

In 2012, 40% of barcodes in InfoScan, representing 81% of sales, were matched to nutrition data, and 48% of the barcodes in Consumer Network, representing 78% of sales, were matched to nutrition data [32]. Analyses of calories and nutrients were conducted on a per-serving basis, but to account for slight differences in serving sizes, we converted all to a consistent weight per serving by product category. For example, if most products listed a half-cup serving as being 30 g, we adjusted the nutrient levels of all other products to be consistent with this serving size weight.

We used IRI Consumer Network household scanner data to calculate nationally representative estimates of food purchase quantities for households with children and adolescents across demographic categories in 2014. Consumer Network data represent weekly barcode-level purchases of foods from a panel of approximately 120,000 households. Approximately one-third of households in the panel have children. We used the “static” portion of the data that represents households with sufficient purchase volumes to be considered representative (65,000 households of which approximately one-third have children). In calculating purchase quantities, we applied the projection factors or weights in the data to determine national estimates.

We used the online Guiding Stars database to identify one-, two-, and three-star products in the selected product categories, and calculated average nutrient levels for each set of products to use as target levels in simulations. Guiding Stars is a voluntary program used by participating grocery retailers in the United States to help consumers identify nutritious foods using shelf labels in stores. Guiding Stars ratings are assigned using an algorithm that considers vitamins, minerals, dietary fiber, whole grains, omega-3 fatty acids, saturated fat, trans fat, added sugar, added sodium, and artificial colors [34,35]. Products that meet the criteria are indicated on shelf labels in several grocery store chains across the country. We also considered the Walmart Better for You criteria, Partnership for a Healthier America (PHA) calculator, and the National Automatic Merchandising Association (NAMA) vending machines standards as the basis for determining the criteria level. The range of products included in the Walmart and NAMA standards is not representative enough of products in the marketplace, and the number of products available from the PHA calculator was too limited for our purposes. Using nutrient levels of products on the market ensures that target levels are achievable and not simply a theoretical construct.

Lastly, we used the 2011 CFBAI criteria to assign foods as more or less healthy for calculating differences in average prices. As noted previously, CFBAI is a voluntary program in the United States in which food companies commit to advertising to children only products that meet category-specific nutrition criteria [36]. We chose to use the CFBAI criteria because they are based on per-serving levels of nutrients available in our dataset (calories, saturated fat, total sugars, and sodium); therefore, we can clearly assign all products of interest. We also considered using the Guiding Stars criteria to assign products, but the algorithm based on nutrients and ingredients is beyond the focus of this study.

#### 2.1.3. Statistical Analysis

Firstly, for the analysis of changes in average nutrient levels by product category over time, we adjusted the per-serving nutrient values (calories), saturated fat (grams), sugar (grams), sodium (milligrams), and dietary fiber (grams) to an equivalent weight basis. We calculated annual averages and standard deviations for 2012 through 2014 and 2015, and conducted *t*-tests to determine if changes were statistically significant. Note that sugar levels were missing from the IRI data for 2015; thus, we compared results for 2012 to 2014 with those for 2012 to 2015 for other nutrients and found no substantial differences. Averages and standard deviations were also calculated for branded and private-label products separately within each product category for 2014, and *t*-tests were conducted.

Next, we calculated the changes in total nutrients in foods purchased by households with children and adolescents if foods were formulated such that average levels of nutrients in 2014 shifted to the average levels of nutrients in foods meeting the Guiding Stars criteria. Specifically, we calculated the total change in nutrient levels, by demographic category, as

(1) $$\Delta NUT_{j,k}=TS_{j}^{D}\times \left(A\_NUT_{j,k}-GS\_NUT_{j,k}\right),$$

where $A\_NUT_{j,k}$ is the average level of nutrient *k* in product category *j* in 2014 InfoScan data, $GS\_NUT_{j,k}$ is the average level of nutrient *k* in product category *j* in the Guiding Stars database, and $TS_{j}^{D}$, the total servings purchased of product category *j* for demographic category *D*, was calculated as

(2) $$TS_{j}^{D}=\overset{T}{\underset{t=1}{\sum }}\overset{N}{\underset{i=1}{\sum }}\frac{W_{h}^{t}\times Units_{i,t}^{j}\times PKGSIZE_{i}}{STDSIZE_{j}}.$$

$W_{h}^{t}$ is the projection factor (or sample weight) of household *h* taking shopping trip *t* in 2014, $Units_{i,t}^{j}$ is the number of units of barcode *i* in product category *j* purchased on shopping trip *t*, $PKGSIZE_{i}$ is the package size of barcode *i* (e.g., grams), and $STDSIZE_{j}$ is the standard size of a serving for product category *j.* The demographic categories included the following:

- Household income: low (less than 185% of poverty line) versus high (equal to or greater than 185% of the poverty line);
- Race and ethnicity: non-Hispanic white, non-Hispanic black, Hispanic, Asian, and other;
- Locality: urban, part urban/rural, and rural.

We converted the nutrient values to a per-day, per-person equivalent basis assuming a diet of 2000 calories per day.

To assess the relative magnitude of the changes that could result from reformulation in terms of the total diet, we divided the total change in nutrient levels, $\Delta NUT_{j,k}$, by the average daily levels of nutrients consumed by the US population, two years of age or older, in What We Eat in America, 2013–2014 [37]. We used the average intake for all ages because the purchase data used in the calculation are for households that comprise adults and children.

Finally, for food categories listed in the 2011 CFBAI criteria, we identified which products did or did not meet the criteria, calculated the average prices of each group, and conducted *t*-tests to determine if the differences were statistically significant at the *p* = 0.05 level.

This study was deemed not to be human subject research by RTI International’s Institutional Review Board.

## 3. Results

Below, we describe results of tracking changes in nutrient levels for selected product categories, simulating potential improvements in foods and beverages, and comparing average prices for healthier and less healthy products on the market.

### 3.1. Changes in Nutrient Levels for Selected Products over Time

Table 1 shows the average per-serving values by product category, nutrient, and brand type (branded and private label) for product categories that had statistically significant changes in mean nutrient levels (*p* = 0.05) that were a minimum of a 5% change (Table S1 shows detailed results including those that were not statistically significant). Results shown in Table 1 compare 2012 with 2014 values because values for sugar were not available in the dataset for 2015. However, means were not substantially different for calories, saturated fat, sodium, and fiber when comparing 2012 with either 2014 or 2015 values. No changes were statistically significant for frankfurters, fruit drinks, ice cream, RTE macaroni and cheese, pizza, or soft drinks for calories or nutrients examined. For product categories with statistically significant changes, mean saturated fat levels decreased for most products. In addition, dietary fiber levels increased in yogurt products. Mean calories and sugar increased for lunch combinations, but dietary fiber also increased.

Table 2 further examines data for 2014 by comparing mean nutrient levels for branded versus private-label products. Results shown are for statistically significant differences (*p* = 0.05), where differences were at least 5% (with the denominator of the percentage calculated as the average of the branded and private-label products) (Table S2 shows all differences including those that were not statistically significant). The top portion of the table shows the nutrients that had better mean levels for branded products, and the bottom portion shows the nutrients that had better mean levels for private-label products. Overall, branded products had more nutrients and more product categories for which levels were healthier than private-label products. In particular, average sodium levels were lower and dietary fiber levels were higher for branded products, but private-label products had more products with lower saturated fat levels.

### 3.2. Results of Simulations of Effects of Reformulation

**Figure 2:**
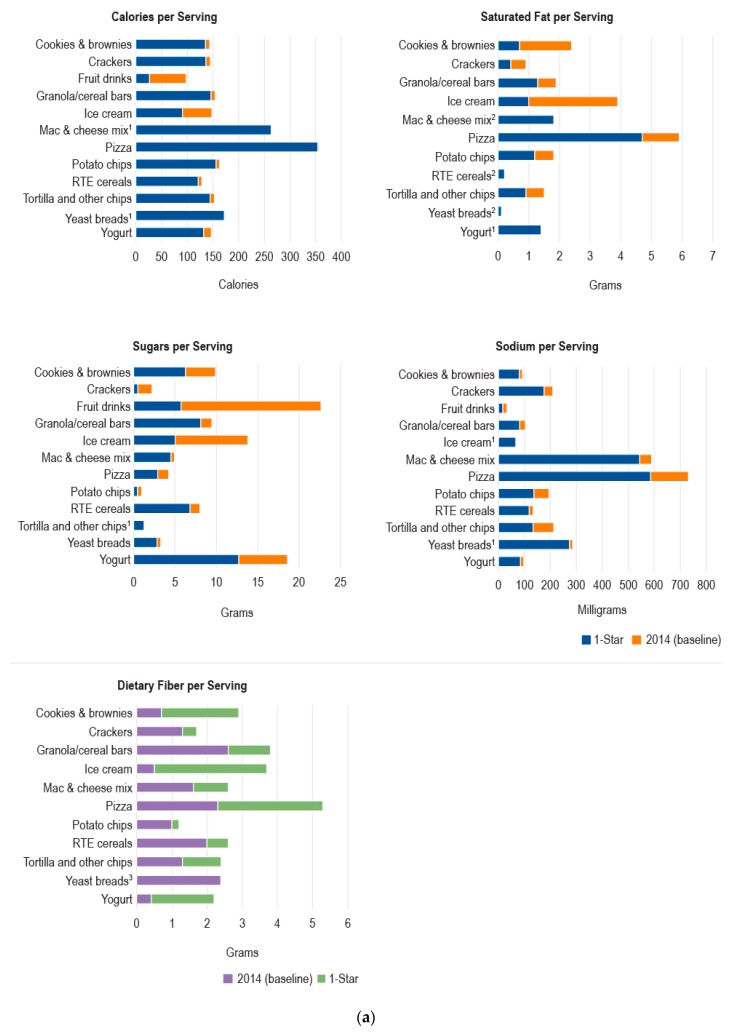

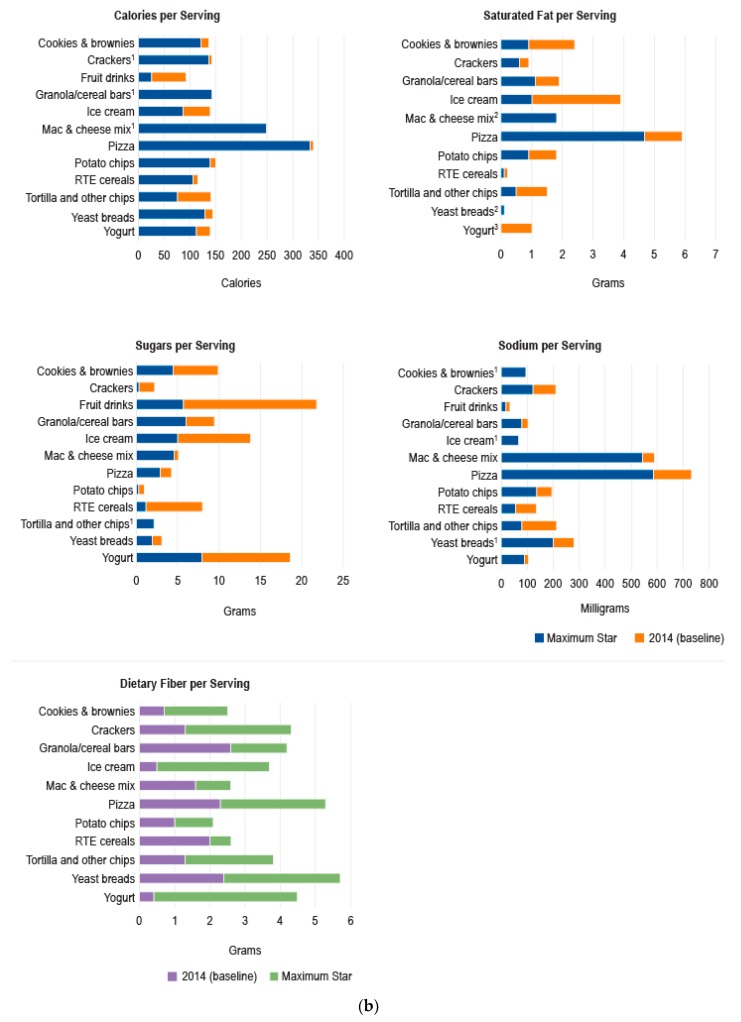
(**a**) Comparison of average calories and nutrient levels in products in 2014 versus one-star products. ^1^ Average 2014 (baseline) value is less than the average one-star value. ^2^ Average 2014 (baseline) value is the same as the average one-star value. ^3^ Average one-star value is less than the average 2014 (baseline) value. (**b**) Comparison of average calories and nutrient levels in products in 2014 versus maximum-star products. ^1^ Average 2014 (baseline) value is less than the average maximum-star value. ^2^ Average 2014 (baseline) value is the same as the average maximum-star value. ^3^ Maximum-star value is zero.

Figure 2a,b compare average nutrient levels in 2014 by product category with those of products that met Guiding Stars criteria for one star or for the maximum number of stars for the product category. No or few (i.e., fewer than five) products met the criteria for at least a one-star rating for frankfurters, fruit drinks, RTE macaroni and cheese, nut butters, and soft drinks, and were, therefore, excluded from the simulation analysis. In Figure 2a, blue bars represent the average one-star product values for calories, saturated fat, sugars, and sodium, and orange bars indicate the decrease in those values that would be required to shift the average values for products in 2014 to those of one-star products. For fiber, purple bars indicate the average values for products in 2014, and green bars represent the increase in fiber that would need to occur to shift to the average fiber values of one-star products. Figure 2b shows the corresponding changes to shift calorie and nutrient values to the average values for maximum-star products. For most products, the largest changes would need to occur for saturated fat, sugars, and fiber. Saturated fat would need to decrease substantially except for macaroni and cheese mix, RTE cereals, yogurt, and yeast breads. Sugars would need to decrease substantially for all products except tortilla and other chips and macaroni and cheese mix. Dietary fiber would need to increase for all products except yeast breads. The average calorie levels are not substantially different except for fruit drinks and ice cream for one-star levels, and for these products and tortilla and other chips for maximum-star products. Required changes for sodium levels are important for some products, such as pizza, macaroni and cheese mix, and potato chips, but are relatively small for most other products. For each nutrient, at least one or two product categories had lower average values for products in 2014 than those of one-star or maximum-star products; however, there was no general pattern for these cases.

Table 3 shows the combined results of simulations for the 12 product categories with enough products on the market that meet at least the one-star criteria (Table S3 shows detailed results by product category and by demographic category). The first row provides the average daily intake for calories and the other nutrients based on 2013–2014 NHANES across males and females, age two or older, and the second row provides the baseline per-person amount in the 12 product categories purchased by households with children in 2014. This means that the 12 product categories represent about 11% of calories, 9% of saturated fat, 11% of sugar, 8% of sodium, and 13% of fiber on average. Shifting the mean level of nutrients in these products to the mean level of products that received a one-star rating has a relatively minor effect on calories but cuts saturated fat in the 12 product categories by more than half (52%), sugar by more than 42%, and sodium by 14%, while it increases dietary fiber by 82%. Relative to average daily intake, the changes for calories and sodium are minimal, but saturated fat consumption would decrease by 4% and sugar consumption by 5%, and dietary fiber consumption would increase by 11%. When we recalculated the changes using the means for the maximum-star-rated products, which could be one, two, or three stars depending on product category, the changes are more substantial. Relative to average daily intake, calories decline by 4%, saturated fat by 6%, sugar by 8%, and sodium by 4%, and dietary fiber increases by 14%.

Table 4 compares the differences in the per-person per-day change in calories and nutrients across demographic groups assuming that mean levels of nutrients shift to those of one-star products and then maximum-star products. The differences shown arise from differences in purchasing patterns of foods across demographic groups, thus affecting both the baseline amount in purchases and the potential change that could be achieved. By income level, all differences are extremely small and exhibit no general patterns. By race and ethnicity, reductions in saturated fat and sodium and increases in dietary fiber are highest for non-Hispanic whites. Other differences are generally mixed depending on which targets are used. However, overall improvements are lowest for Asians and others, and Hispanics are in between other groups; both groups had lower initial levels of calories, saturated fat, sugar, and sodium, but also lower initial levels of fiber for the set of product categories included in the analysis. Results by urban or rural location vary little except for slightly larger reductions in sodium for part urban/rural and rural households compared with urban locations.

### 3.3. Results of Average Price Calculations for Healthier versus Less Healthy Products

Table 5 shows results comparing mean prices between healthier and less healthy versions of foods in six categories using the CFBAI criteria to define healthier (Table S4 shows detailed results by individual product category). Of the 12 product categories included in the simulation analysis, two were omitted from the price comparisons because there are no applicable CFBAI criteria (juice drinks and cookies and brownies), and three were omitted because too few items met the CFBAI criteria for a healthier product (macaroni and cheese mix, pizza, and potato chips). Ice cream was also omitted because the serving size information in our data is provided in gram-weight, but the CFBAI criteria are based on a half-cup serving; therefore, we were unable to determine which products met the criteria. In four categories (crackers, tortilla and other chips, yeast bread, and yogurt), the healthier items cost between 3 and 12 cents more per serving, on average, than less healthy items that do not meet the CFBAI criteria. These healthier items also make up only a small share of the total servings of all items for which we have nutritional information in those categories, except in the case of yogurt, where items that meet the CFBAI criteria comprise more than 60% of the servings sold. On average, healthier cereal bars cost 34 cents less per serving than those that do not meet the CFBAI criteria; however, the opposite is true among only private-label cereal bars, where the healthier items cost 7 cents more per serving. The comparison of prices is also different when looking at all items versus private label only in the RTE cereal category. Although we found no difference in price per serving among healthier and less healthy items, on average, for RTE cereals, healthier private-label RTE cereals cost 3 cents per serving less than less healthy private-label cereals.

## 4. Discussion

Reformulation may be an option to improve child and adolescent dietary quality by changing the nature of the food supply. Instead of focusing on changing consumer behavior, this study focuses on assessing potential effects of changing producer behavior toward producing healthier foods. This study is particularly relevant given increasing interest in voluntary industry initiatives in which food companies committed to improving the healthfulness of foods and beverages purchased by consumers. Examples of recent commitments by food companies that focus on or encourage reformulation include the Healthy Weight Commitment, the Balance Calories Initiative, and CFBAI criteria. In addition, the upcoming changes in the nutrition facts label provide an incentive for reformulation, particularly to reduce added sugars.

Improvements in packaged foods and beverages frequently consumed by children and adolescents over the 2012–2014 time period appeared to focus primarily on saturated fat. Calories and other nutrients had no or very small changes. The observed changes in levels of nutrients were similar across branded and private-label products. Although the results varied across product categories and nutrients, branded products tended to have better nutrient profiles than private-label products based on labeled nutrients. These results are in contrast to Ahuja et al. [38], who found no statistically significant differences in nutrients through chemical analysis of 937 branded and private-label products.

Our analysis focused on calculating changes in average levels of nutrients over time rather than tracking changes in nutrients in specific food products because manufacturers might assign new barcodes to existing products when they are reformulated or relabeled. Martinez and Levin [39] tracked changes in nutritional quality of breakfast cereals, yogurt, snacks, candy, and frozen and refrigerated meals from 2008 through 2012 using IRI store scanner data, and defined new barcodes as new products because of the difficulties in sorting out which products are newly introduced versus reformulated or relabeled versions of current products. Because of how manufacturers assign and reassign barcodes, identifying and tracking reformulations of specific food products would require detailed comparisons of the text of product descriptions and some amount of guesswork.

Results of simulation show that reformulating a few categories of foods and beverages to reach the levels of products meeting an existing standard could improve dietary intake particularly for saturated fat, sugars, and fiber. For calories, the results show relatively modest reductions, which could be because manufacturers balance taste profiles when adjusting fat, sugar, and sodium levels in foods. Interestingly, the results for saturated fat show that substantial improvements could occur due to reformulation even though this was the nutrient for which we observed the greatest changes over the 2012–2014 time period. These results indicate there is still room for improvement in saturated fat levels in packaged foods. Results of simulation for sugar show substantially lower reductions than other studies that focused exclusively on reformulation to reduce sugar [40]. Although we cannot distinguish added from naturally occurring sugars in our data, it is likely that most of the potential improvement in sugars would come from reductions in added sugars given that our analysis focuses on packaged foods. After the new nutrition facts label becomes mandatory, nutrition label data from commercial data suppliers should also include added sugars, thus allowing analyses to distinguish the two. Sodium showed the smallest simulated improvements of the nutrients analyzed. It could be that manufacturers already reformulated foods to lower sodium levels given the emphasis on reducing sodium in recommendations from the Institute of Medicine (IOM) (now National Academies of Sciences, Engineering, and Medicine) in 2010 [41]. Following release of the IOM report, FDA developed draft guidance for the industry to reduce sodium levels in foods on a voluntary basis rather than through the regulatory approach recommended in the IOM report [42]. Food manufacturers might, therefore, have focused on reducing sodium levels to avoid possible regulation. Finally, results of simulation show an almost doubling of dietary fiber levels; however, in some cases, such as yogurt, the fiber levels in foods that meet the existing standard appear to be through adding supplemental fiber.

Results of the simulations suggest that reformulation would improve diet quality more for non-Hispanic white households than for other household types and that differences across income groups were extremely small. However, the results are affected by the extent to which each type of household buys the categories of foods included in the analyses. Simulations using a different set of product categories, particularly those purchased more frequently by lower income, ethnic, and minority populations, could indicate a different pattern of effects.

Some argue that cost is a barrier to a healthy diet because less healthy foods cost less per calorie [43]. In contrast, other research showed that, when measured per serving, prices of healthier foods such as grains, vegetables, fruit, and dairy foods are less expensive than foods with a lot of saturated fat, sodium, and added sugar [44]. Our analysis looked within food categories and found that healthier items are often more expensive per serving, on average, than less healthy items within the same category. If it is more expensive to produce items of higher nutritional quality, then reformulation may result in higher prices for consumers. Higher prices could limit the benefits of reformulation if higher prices push consumers to choose less healthful items.

Analyses in this study were subject to several limitations. Because we used household-based scanner data to measure food purchases, we were unable to track consumption by children and adolescents per se, only purchases by households with children and adolescents. However, no databases are available to track consumption of foods by children and adolescents at the barcode level as is needed for examining reformulation trends. In addition, the IRI household-based scanner data have specific limitations that are documented in Muth et al. [32]. For example, these data represent only foods purchased from grocery stores and similar retail outlets (i.e., not restaurants or cafeterias), while not all barcodes are matched to nutrient data, and some types of households are less well represented in the data. Despite their limitations, IRI scanner data provide an extensive representation of the food supply in the United States. Also, these data are the only data available in the United States that include both sales and nutrients within a single source and, thus, greatly facilitated the analysis. Finally, when using nutrition data based on the labels, it is also important to keep in mind that, based on FDA regulation, the nutrition information on the label is subject to rounding rules [12,45]. Therefore, minor reformulations may be occurring that are not reflected on the label. However, if manufacturers are reformulating foods to improve healthfulness, most changes are likely detectable on the nutrition facts label.

## 5. Conclusions

Except for calories, a substantial improvement in diet quality for households with children and adolescents could occur from reformulating just 12 categories of foods based on current purchasing patterns. However, reformulation could affect sensory characteristics of foods, particularly if products are reformulated to meet nutrient levels consistent with two- and three-star products and, thus, change purchasing patterns. Encouraging manufacturers to pursue at least moderate reformulation of foods commonly purchased by households with children and adolescents could improve health outcomes, but may have a limited effect on total calories consumed and, hence, weight status.

## Figures and Tables

**Table 1:** Statistically significant changes in average nutrient levels for branded and private-label products (minimum 5% change), 2012–2014. RTE—ready-to-eat.

Product ^1^	Nutrient	Brand Type	No. of Barcodes (2012)	Average Per Standardized Serving Values			
				2012	2014	Difference	*p-*Value
Cereal bars	Saturated fat (grams)	Branded	2086	2.14	1.95	−0.19	<0.01
		Private label	539	1.63	1.28	−0.35	<0.01
Cookies and brownies	Saturated fat (grams)	Branded	3530	2.55	2.38	−0.17	<0.01
		Private label	1741	2.44	2.26	−0.18	<0.01
Crackers	Saturated fat (grams)	Branded	1934	1.10	0.95	−0.15	<0.01
		Private label	866	0.94	0.76	−0.18	<0.01
Lunch combinations ^2^	Calories	Branded	95	320.06	346.88	26.82	0.02
		Private label	0	--	--	--	--
	Sugar (grams)	Branded	95	17.35	19.17	1.82	0.05
		Private label	0	--	--	--	--
	Dietary fiber (grams)	Branded	95	1.33	1.44	0.11	0.03
		Private label	0	--	--	--	--
Macaroni and cheese mix	Saturated fat (grams)	Branded	181	2.10	1.91	−0.19	<0.01
		Private label	310	1.82	1.67	−0.15	<0.01
Nut butters	Saturated fat (grams)	Branded	344	2.62	2.42	−0.2	<0.01
		Private label	394	3.17	2.92	−0.25	<0.01
Potato chips	Saturated fat (grams)	Branded	1,745	2.02	1.82	−0.20	<0.01
		Private label	381	2.10	1.93	−0.17	<0.01
RTE cereal	Saturated fat (grams)	Branded	1403	0.33	0.24	−0.09	<0.01
		Private label	1718	0.13	0.09	−0.04	<0.01
Tortilla chips and other chips	Saturated fat (grams)	Branded	1681	1.80	1.56	−0.24	<0.01
		Private label	375	1.33	1.15	−0.18	<0.01
Yeast breads	Saturated fat (grams)	Branded	1939	0.18	0.14	−0.04	<0.01
		Private label	1290	0.16	0.13	−0.03	<0.01
Yogurt	Saturated fat (grams)	Branded	1030	1.46	1.38	−0.08	<0.01
		Private label	998	0.54	0.49	−0.05	<0.01
	Dietary fiber (grams)	Branded	1030	0.30	0.32	0.02	<0.01

^1^ Frankfurters, fruit drinks, ice cream, RTE macaroni and cheese, pizza, and soft drinks had no statistically significant changes of more than 5%. ^2^ Although lunch combinations improved with regard to dietary fiber, average calories and sugar levels increased. Improvements in saturated fat levels were not statistically significant.

**Table 2:** Statistically significant difference in average nutrient levels between branded and private- label products (minimum 5% difference), 2014.

Nutrient	Product ^1^	Average Per Standardized Serving Values			
		Branded	Private Label	Difference (Private Label − Branded)	*p-*Value
**Branded Better than Private Label**					
Calories	Frankfurters	156.42	168.09	11.67	<0.01
	Fruit drinks	92.89	101.68	8.79	<0.01
Saturated fat (grams)	Frankfurters	4.83	5.20	0.37	0.02
	Macaroni and cheese (RTE)	7.06	9.50	2.44	0.04
	Nut butters	2.47	2.89	0.42	<0.01
Sugar (grams)	Cereal bars	9.17	10.52	1.35	<0.01
	Crackers	2.01	2.66	0.65	<0.01
	Frankfurters	1.03	1.49	0.46	<0.01
	Fruit drinks	21.82	25.07	3.25	<0.01
Sodium (milligrams)	Cookies and brownies	88.61	99.66	11.05	<0.01
	Frankfurters	532.70	570.98	38.28	<0.01
	Fruit drinks	32.59	39.71	7.12	<0.01
	Macaroni and cheese mix	572.18	604.93	32.75	<0.01
	Nut butters	88.58	116.08	27.50	<0.01
	RTE cereal	119.20	150.33	31.13	<0.01
	Yeast breads	270.90	290.83	19.93	<0.01
	Yogurt	89.25	96.1	6.85	<0.01
Dietary fiber (grams)	Cereal bars	2.64	2.36	−0.28	<0.01
	Cookies and brownies	0.78	0.58	−0.20	<0.01
	Crackers	1.37	1.00	−0.37	<0.01
	Ice cream	0.63	0.32	−0.31	<0.01
	Nut butters	2.25	2.00	−0.25	<0.01
	Pizza	2.38	2.05	−0.33	<0.01
	Potato chips	1.04	0.98	−0.06	<0.01
	RTE cereal	2.17	1.89	−0.28	<0.01
	Yeast breads	2.64	2.01	−0.63	<0.01
**Private Label Better than Branded**					
Calories	Yogurt	146.7	127.18	−19.52	<0.01
Saturated fat (grams)	Cereal bars	1.96	1.41	−0.55	<0.01
	Crackers	0.99	0.78	−0.21	<0.01
	Macaroni and cheese (mix)	1.88	1.64	−0.24	0.04
	Pizza	6.11	5.52	−0.59	<0.01
	RTE cereal	0.28	0.08	−0.20	<0.01
	Tortilla and other chips	1.52	1.13	−0.39	<0.01
	Yogurt	1.39	0.44	−0.95	<0.01
Sugar (grams)	Macaroni and cheese (mix)	5.04	4.39	−0.65	<0.01
	Pizza	4.46	3.69	−0.77	<0.01
	Potato chips	0.89	0.71	−0.18	<0.01
	Yogurt	19.05	17.82	−1.23	<0.01
Sodium (milligrams)	Cereal bars	105.22	97.88	−7.34	<0.01
	Soft drinks	29.62	21.09	−8.53	<0.01
	Tortilla and other chips	220.86	171.92	−48.94	<0.01

^1^ No private-label lunch combinations appear in the dataset.

**Table 3:** Results of simulating the effects of reformulating 12 food and beverage categories to meet an existing standard (per person-equivalent per day).

	Calories	Saturated Fat (g)	Sugar (g)	Sodium (mg)	Dietary Fiber (g)
NHANES total daily intake ^1^	2079	26.3	112	3409	16.3
Baseline amount in purchases (2014)	220.23	2.25	12.76	265.24	2.20
Average amount in 1-star products	202.35	1.09	7.47	227.89	4.00
Change to average 1-star target	−17.88	−1.16	−5.29	−37.35	1.80
% change relative to baseline purchases	−8.1%	−51.6%	−41.5%	−14.1%	81.8%
% change relative to NHANES daily intake	−0.9%	−4.4%	−4.7%	−1.1%	11.0%
Average amount in maximum-star products	137.41	0.62	3.26	143.01	4.41
Change to average maximum-star target	−82.83	−1.63	−9.49	−122.23	2.21
% change relative to baseline purchases	−37.6%	−72.4%	−74.4%	−46.1%	100.5%
% change relative to NHANES daily intake	−4.0%	−6.2%	−8.5%	−3.6%	13.6%

^1^ NHANES total daily intake represents males and females, age two or older, obtained from What We Eat in America, 2013–2014. https://www.ars.usda.gov/northeast-area/beltsville-md-bhnrc/beltsville-human-nutrition-research-center/food-surveys-research-group/docs/wweia-data-tables/.

**Table 4:** Differences in potential improvements from reformulating 12 food and beverage categories by demographic group (per person-equivalent per day).

Household Demographic	Calories	Saturated Fat (g)	Sugar (g)	Sodium (mg)	Dietary Fiber (g)
	**Change Using Average 1-Star Targets**				
All	−17.9	−1.2	−5.3	−37.4	1.8
Income level:					
Low income	−18.3	−1.2	−5.4	−36.7	1.8
High income	−17.6	−1.2	−5.2	−37.7	1.8
Race and ethnicity:					
Non-Hispanic white	−18.1	−1.3	−5.4	−41.0	2.0
Non-Hispanic black	−21.4	−1.0	−6.1	−32.2	1.5
Hispanic	−16.4	−0.9	−4.9	−31.1	1.5
Asian and other	−14.6	−1.0	−4.3	−28.3	1.5
Urban vs. rural:					
Urban	−18.1	−1.1	−5.3	−36.0	1.8
Part urban/rural	−17.6	−1.2	−5.2	−40.0	1.9
Rural	−17.2	−1.3	−5.2	−40.5	1.9
	**Change Using Maximum-Star Targets**				
All	−82.8	−1.6	−9.5	−122.2	2.2
Income level:					
Low income	−81.1	−1.6	−9.5	−120.4	2.2
High income	−83.9	−1.7	−9.5	−123.4	2.2
Race and ethnicity:					
Non-Hispanic white	−88.6	−1.8	−10.0	−133.4	2.4
Non-Hispanic black	−78.8	−1.4	−9.7	−106.1	1.8
Hispanic	−71.1	−1.3	−8.5	−102.3	1.8
Asian and other	−67.1	−1.3	−7.6	−95.7	1.8
Urban vs. rural:					
Urban	−81.5	−1.6	−9.5	−119.0	2.2
Part urban/rural	−85.2	−1.7	−9.5	−128.4	2.3
Rural	−87.8	−1.8	−9.5	−131.5	2.3

**Table 5:** Comparison of sales volumes and price per serving for healthier versus less healthy products using the Children’s Food and Beverage Advertising Initiative (CFBAI) criteria, 2014.

	Meets CFBAI Criteria			Does Not Meet CFBAI Criteria				*p*-Value
	*N*	% of Servings for Products with Nutritional Information	$/Serving	*N*	% of Servings for Products with Nutritional Information	$/Serving	Difference (Meets − Does Not Meet)	
Cereal bars	166	4.3	$0.62	2292	95.7	$0.96	−$0.34	<0.0001
Private label	35	10.6	$0.44	311	89.4	$0.37	$0.07	0.0007
Crackers	202	8.6	$0.43	2281	91.4	$0.36	$0.07	0.0002
Tortilla and other chips	125	1.5	$0.47	1783	98.5	$0.35	$0.12	<0.0001
RTE cereal	342	13.3	$0.28	2401	86.7	$0.29	−$0.01	0.8199
Private label	122	14.2	$0.18	1121	85.8	$0.21	−$0.03	<0.0001
Yeast bread	558	15.7	$0.28	2521	84.3	$0.25	$0.03	0.0594
Yogurt	1126	60.6	$0.82	797	39.4	$0.76	$0.06	<0.0001

Note: For all categories listed except cereal bars and RTE cereal, price comparisons are qualitatively similar in direction and statistical significance when considering national brand and private-label items separately.

## Supplementary Materials

The following are available online at https://www.mdpi.com/2072-6643/11/3/618/s1: Table S1: Detailed Version of Table 1; Table S2: Detailed Version of Table 2; Table S3: Detailed Version of Table 3 and Table 4; and Table S4: Detailed Version of Table 5.
