# Supplementary material for: How Much Can Product Reformulation Improve Diet Quality in Households with Children and Adolescents?
Source: Nutrients. 2019 Mar 14;11(3):618. doi: 10.3390/nu11030618 (PMC6470779; doi:10.3390/nu11030618)
Supplement: Supplementary file 1 [file nutrients-11-00618-s001.zip › Table S4 Detailed Version of Table 5.pdf]

Number of UPC's, Units and Total Sales of UPC's that Meet CFBAI Criteria (2014): Cereal Bars

Calculations using InfoScan Data for UPCs with positive sales

	Brand Type	N UPCs	% UPCs	N Units	% Units	$ Sales	% Sales	N Servings*	$/Serving
Meets CFBAI criteria	NATIONAL	131	3.1	108,631,565	8.2	337,579,810	8.9	687,014,080	$0.67
	PRIVATE LABEL	35	5.3	2,053,543	3.4	4,406,575	3.7	13,643,576	$0.44
	ALL	166	3.4	110,685,107	8.0	341,986,385	8.7	697,360,656	$0.62
Does not meet CFBAI criteria	NATIONAL	1,981	46.2	1,129,187,543	85.7	3,055,452,778	80.6	5,602,119,366	$1.05
	PRIVATE LABEL	311	46.9	46,318,025	76.6	91,491,427	76.0	303,028,455	$0.37
	ALL	2,292	46.3	1,175,505,568	85.3	3,146,944,205	80.5	5,905,147,821	$0.96
No nutrition	NATIONAL	2,173	50.7	80,265,586	6.1	396,552,429	10.5		
	PRIVATE LABEL	317	47.8	12,078,427	20.0	24,532,550	20.4		
	ALL	2,490	50.3	92,344,013	6.7	421,084,979	10.8		
Total	NATIONAL	4,285	86.6	1,318,084,694	95.6	3,789,585,017	96.9		
	PRIVATE LABEL	663	13.4	60,449,994	4.4	120,430,552	3.1		
	ALL	4,948	100.0	1,378,534,688	100.0	3,910,015,570	100.0		

*Serving size = Labeled serving size (LSS)

Test for Price Differences - Meets vs Does Not Meet (2014): Cereal Bars

	Meets CFBAI criteria		Does not meet CFBAI criteria		Difference	
	N	$/Serving	N	$/Serving	(Meets-Not meet)	P-value
NATIONAL	131	$0.67	1,981	$1.05	-$0.38	<0.0001
PRIVATE LABEL	35	$0.44	311	$0.37	$0.07	0.0007
ALL	166	$0.62	2,292	$0.96	-$0.34	<0.0001

Number of UPC's, Units and Total Sales of UPC's that Meet CFBAI Criteria (2014): Crackers

Calculations using InfoScan Data for UPCs with positive sales

	Brand Type	N UPCs	% UPCs	N Units	% Units	$ Sales	% Sales	N Servings*	$/Serving
Meets CFBAI criteria	NATIONAL	155	3.7	202,660,578	11.4	545,545,282	11.5	1,782,567,619	$0.47
	PRIVATE LABEL	47	3.9	3,192,431	3.5	6,674,379	3.5	25,505,589	$0.31
	ALL	202	3.7	205,853,009	11.0	552,219,661	11.2	1,808,073,207	$0.43
Does not meet CFBAI criteria	NATIONAL	1,746	41.5	1,451,475,273	81.7	3,819,192,089	80.4	18,228,157,454	$0.41
	PRIVATE LABEL	535	44.9	75,003,812	81.1	151,351,495	79.8	1,018,282,855	$0.21
	ALL	2,281	42.2	1,526,479,085	81.7	3,970,543,584	80.4	19,246,440,309	$0.36
No nutrition	NATIONAL	2,310	54.9	122,491,775	6.9	383,182,186	8.1		
	PRIVATE LABEL	609	51.1	14,268,124	15.4	31,742,165	16.7		
	ALL	2,919	54.0	136,759,899	7.3	414,924,351	8.4		
Total	NATIONAL	4,211	78.0	1,776,627,626	95.1	4,747,919,557	96.2		
	PRIVATE LABEL	1,191	22.0	92,464,367	4.9	189,768,039	3.8		
	ALL	5,402	100.0	1,869,091,993	100.0	4,937,687,596	100.0		

*Serving size = Labeled serving size (LSS)

Test for Price Differences - Meets vs Does Not Meet (2014): Crackers

	Meets CFBAI criteria		Does not meet CFBAI criteria		Difference	
	N	$/Serving	N	$/Serving	(Meets-Not meet)	P-value
NATIONAL	155	$0.47	1,746	$0.41	$0.06	0.0063
PRIVATE LABEL	47	$0.31	535	$0.21	$0.10	0.0002
ALL	202	$0.43	2,281	$0.36	$0.07	0.0002

Number of UPC's, Units and Total Sales of UPC's that Meet CFBAI Criteria (2014): Pizza

Calculations using InfoScan Data for UPCs with positive sales

	Brand Type	N UPCs	% UPCs	N Units	% Units	$ Sales	% Sales	N Servings*	$/Serving
Meets CFBAI criteria	NATIONAL	16	0.8	14,885,701	1.7	45,847,025	1.6	18,205,575	$2.72
	PRIVATE LABEL	0	0.0	0	0.0	0	0.0	0	$0.00
	ALL	16	0.6	14,885,701	1.5	45,847,025	1.5	18,205,575	$2.72
Does not meet CFBAI criteria	NATIONAL	921	47.5	848,469,118	95.9	2,678,175,388	94.2	2,804,445,339	$1.43
	PRIVATE LABEL	538	56.2	70,512,411	87.2	232,874,771	87.6	274,781,095	$1.06
	ALL	1,459	50.4	918,981,529	95.2	2,911,050,159	93.6	3,079,226,433	$1.29
No nutrition	NATIONAL	1,000	51.6	21,292,408	2.4	118,695,794	4.2		
	PRIVATE LABEL	419	43.8	10,338,750	12.8	33,102,712	12.4		
	ALL	1,419	49.0	31,631,158	3.3	151,798,506	4.9		
Total	NATIONAL	1,937	66.9	884,647,227	91.6	2,842,718,207	91.4		
	PRIVATE LABEL	957	33.1	80,851,161	8.4	265,977,483	8.6		
	ALL	2,894	100.0	965,498,388	100.0	3,180,695,690	100.0		

*Serving size = Labeled serving size (LSS)

Test for Price Differences - Meets vs Does Not Meet (2014): Pizza

	Meets CFBAI criteria		Does not meet CFBAI criteria		Difference	
	N	$/Serving	N	$/Serving	(Meets-Not meet)	P-value
NATIONAL	16	$2.72	921	$1.43	$1.29	<0.0001
PRIVATE LABEL	0	$0.00	538	$1.06	-$1.06	
ALL	16	$2.72	1,459	$1.29	$1.43	<0.0001

Number of UPC's, Units and Total Sales of UPC's that Meet CFBAI Criteria (2014): Potato Chips

Calculations using InfoScan Data for UPCs with positive sales

	Brand Type	N UPCs	% UPCs	N Units	% Units	$ Sales	% Sales	N Servings*	$/Serving
Meets CFBAI criteria	NATIONAL	3	0.1	236,623	0.02	838,270	0.03	970,661	$0.97
	PRIVATE LABEL	0	0.0	0	0.00	0	0.00	0	$0.00
	ALL	3	0.1	236,623	0.02	838,270	0.02	970,661	$0.97
Does not meet CFBAI criteria	NATIONAL	1,640	54.1	1,276,024,034	96.3	3,083,222,641	95.9	10,288,466,787	$0.40
	PRIVATE LABEL	336	56.4	86,566,526	87.7	163,121,868	89.5	830,548,169	$0.23
	ALL	1,976	54.5	1,362,590,560	95.7	3,246,344,509	95.6	11,119,014,956	$0.37
No nutrition	NATIONAL	1,388	45.8	49,325,945	3.7	130,236,664	4.1		
	PRIVATE LABEL	260	43.6	12,110,183	12.3	19,172,411	10.5		
	ALL	1,648	45.4	61,436,128	4.3	149,409,075	4.4		
Total	NATIONAL	3,031	83.6	1,325,586,602	93.1	3,214,297,575	94.6		
	PRIVATE LABEL	596	16.4	98,676,709	6.9	182,294,279	5.4		
	ALL	3,627	100.0	1,424,263,311	100.0	3,396,591,854	100.0		

*Serving size = Labeled serving size (LSS)

Test for Price Differences - Meets vs Does Not Meet (2014): Potato Chips

	Meets CFBAI criteria		Does not meet CFBAI criteria		Difference	
	N	$/Serving	N	$/Serving	(Meets-Not meet)	P-value
NATIONAL	3	$0.97	1,640	$0.40	$0.57	<0.0001
PRIVATE LABEL	0	$0.00	336	$0.23	-$0.23	
ALL	3	$0.97	1,976	$0.37	$0.60	<0.0001

Number of UPC's, Units and Total Sales of UPC's that Meet CFBAI Criteria (2014): RTE Cereal

Calculations using InfoScan Data for UPCs with positive sales

	Brand Type	N UPCs	% UPCs	N Units	% Units	$ Sales	% Sales	N Servings*	$/Serving
Meets CFBAI criteria	NATIONAL	220	8.7	284,962,491	14.6	956,929,183	15.0	3,853,245,885	$0.34
	PRIVATE LABEL	122	6.5	16,395,921	10.7	38,322,200	10.2	240,788,974	$0.18
	ALL	342	7.8	301,358,412	14.3	995,251,383	14.7	4,094,034,858	$0.28
Does not meet CFBAI criteria	NATIONAL	1,280	50.8	1,599,716,093	81.7	5,185,664,699	81.2	23,221,430,412	$0.35
	PRIVATE LABEL	1,121	59.8	117,768,075	76.6	283,034,187	75.6	1,572,274,018	$0.21
	ALL	2,401	54.7	1,717,484,168	81.4	5,468,698,886	80.9	24,793,704,430	$0.29
No nutrition	NATIONAL	1,019	40.5	72,319,777	3.7	244,072,550	3.8		
	PRIVATE LABEL	631	33.7	19,524,795	12.7	52,910,823	14.1		
	ALL	1,650	37.6	91,844,572	4.4	296,983,373	4.4		
Total	NATIONAL	2,519	57.3	1,956,998,361	92.7	6,386,666,432	94.5		
	PRIVATE LABEL	1,874	42.7	153,688,791	7.3	374,267,210	5.5		
	ALL	4,393	100.0	2,110,687,152	100.0	6,760,933,642	100.0		

*Serving size = Labeled serving size (LSS)

Test for Price Differences - Meets vs Does Not Meet (2014): RTE Cereal

	Meets CFBAI criteria		Does not meet CFBAI criteria		Difference	
	N	$/Serving	N	$/Serving	(Meets-Not meet)	P-value
NATIONAL	220	$0.34	1,280	$0.35	-$0.01	0.6048
PRIVATE LABEL	122	$0.18	1,121	$0.21	-$0.03	<0.0001
ALL	342	$0.28	2,401	$0.29	-$0.01	0.8199

Number of UPC's, Units and Total Sales of UPC's that Meet CFBAI Criteria (2014): Tortilla and Other Chips

Calculations using InfoScan Data for UPCs with positive sales

	Brand Type	N UPCs	% UPCs	N Units	% Units	$ Sales	% Sales	N Servings*	$/Serving
Meets CFBAI criteria	NATIONAL	113	3.1	38,681,738	2.1	100,587,559	2.3	232,769,835	$0.49
	PRIVATE LABEL	12	2.0	691,216	0.9	1,971,137	1.5	8,190,514	$0.29
	ALL	125	3.0	39,372,954	2.0	102,558,696	2.3	240,960,349	$0.47
Does not meet CFBAI criteria	NATIONAL	1,480	41.1	1,708,266,954	91.7	4,045,748,816	91.3	15,605,001,398	$0.37
	PRIVATE LABEL	303	51.2	65,372,537	85.7	113,403,076	84.0	681,416,849	$0.22
	ALL	1,783	42.6	1,773,639,491	91.5	4,159,151,892	91.1	16,286,418,247	$0.35
No nutrition	NATIONAL	2,004	55.7	116,165,708	6.2	283,837,651	6.4		
	PRIVATE LABEL	277	46.8	10,231,609	13.4	19,681,049	14.6		
	ALL	2,281	54.5	126,397,317	6.5	303,518,700	6.6		
Total	NATIONAL	3,597	85.9	1,863,114,400	96.1	4,430,174,026	97.0		
	PRIVATE LABEL	592	14.1	76,295,362	3.9	135,055,262	3.0		
	ALL	4,189	100.0	1,939,409,762	100.0	4,565,229,288	100.0		

*Serving size = Labeled serving size (LSS)

Test for Price Differences - Meets vs Does Not Meet (2014): Tortilla and Other Chips

	Meets CFBAI criteria		Does not meet CFBAI criteria		Difference	
	N	$/Serving	N	$/Serving	(Meets-Not meet)	P-value
NATIONAL	113	$0.49	1,480	$0.37	$0.12	<0.0001
PRIVATE LABEL	12	$0.29	303	$0.22	$0.07	0.0829
ALL	125	$0.47	1,783	$0.35	$0.12	<0.0001

Number of UPC's, Units and Total Sales of UPC's that Meet CFBAI Criteria (2014): Yeast Breads

Calculations using InfoScan Data for UPCs with positive sales

	Brand Type	N UPCs	% UPCs	N Units	% Units	$ Sales	% Sales	N Servings*	$/Serving
Meets CFBAI criteria	NATIONAL	438	7.5	394,838,774	22.1	1,158,479,367	23.3	4,997,857,620	$0.30
	PRIVATE LABEL	120	4.3	31,157,445	5.2	67,194,531	6.6	443,466,407	$0.19
	ALL	558	6.5	425,996,219	17.8	1,225,673,898	20.5	5,441,324,027	$0.28
Does not meet CFBAI criteria	NATIONAL	1,498	25.7	1,308,268,787	73.1	3,550,778,139	71.4	21,509,356,040	$0.28
	PRIVATE LABEL	1,023	36.8	483,938,899	81.0	757,949,617	74.5	7,762,317,034	$0.20
	ALL	2,521	29.3	1,792,207,686	75.1	4,308,727,756	71.9	29,271,673,075	$0.25
No nutrition	NATIONAL	3,895	66.8	87,366,251	4.9	265,212,939	5.3		
	PRIVATE LABEL	1,636	58.9	82,027,744	13.7	192,577,422	18.9		
	ALL	5,531	64.2	169,393,995	7.1	457,790,361	7.6		
Total	NATIONAL	5,831	67.7	1,790,473,812	75.0	4,974,470,445	83.0		
	PRIVATE LABEL	2,779	32.3	597,124,088	25.0	1,017,721,570	17.0		
	ALL	8,610	100.0	2,387,597,900	100.0	5,992,192,015	100.0		

*Serving size = Labeled serving size (LSS)

Test for Price Differences - Meets vs Does Not Meet (2014): Yeast Breads

	Meets CFBAI criteria		Does not meet CFBAI criteria		Difference	
	N	$/Serving	N	$/Serving	(Meets-Not meet)	P-value
NATIONAL	438	$0.30	1,498	$0.28	$0.02	0.2158
PRIVATE LABEL	120	$0.19	1,023	$0.20	-$0.01	0.1613
ALL	558	$0.28	2,521	$0.25	$0.03	0.0594

Number of UPC's, Units and Total Sales of UPC's that Meet CFBAI Criteria (2014): Yogurt

Calculations using InfoScan Data for UPCs with positive sales

	Brand Type	N UPCs	% UPCs	N Units	% Units	$ Sales	% Sales	N Servings*	$/Serving
Meets CFBAI criteria	NATIONAL	631	24.0	1,134,383,289	34.4	1,875,316,188	39.5	1,916,137,956	$0.96
	PRIVATE LABEL	495	31.3	237,374,727	55.6	190,685,135	55.7	326,625,113	$0.65
	ALL	1,126	26.7	1,371,758,016	36.8	2,066,001,323	40.6	2,242,763,069	$0.82
Does not meet CFBAI criteria	NATIONAL	543	20.6	685,899,004	20.8	1,097,545,110	23.1	1,269,843,981	$0.89
	PRIVATE LABEL	254	16.1	105,301,374	24.7	83,269,301	24.3	191,344,519	$0.47
	ALL	797	18.9	791,200,378	21.3	1,180,814,412	23.2	1,461,188,499	$0.76
No nutrition	NATIONAL	1,460	55.4	1,475,709,328	44.8	1,779,725,539	37.4	2,672,167,856	$0.92
	PRIVATE LABEL	831	52.6	84,129,735	19.7	68,364,159	20.0	109,140,850	$0.71
	ALL	2,291	54.4	1,559,839,063	41.9	1,848,089,697	36.3	2,781,308,707	$0.84
Total	NATIONAL	2,634	62.5	3,295,991,621	88.5	4,752,586,837	93.3	5,858,149,793	$0.92
	PRIVATE LABEL	1,580	37.5	426,805,836	11.5	342,318,595	6.7	627,110,482	$0.65
	ALL	4,214	100.0	3,722,797,457	100.0	5,094,905,432	100.0	6,485,260,275	$0.82

*Serving size = 6 ounces (170 grams)

Test for Price Differences - Meets vs Does Not Meet (2014): Yogurt

	Meets CFBAI criteria		Does not meet CFBAI criteria		Difference	
	N	$/Serving	N	$/Serving	(Meets-Not meet)	P-value
NATIONAL	631	$0.96	543	$0.89	$0.07	0.0060
PRIVATE LABEL	495	$0.65	254	$0.47	$0.18	<0.0001
ALL	1,126	$0.82	797	$0.76	$0.06	<0.0001
