# Supplementary material for: How Much Can Product Reformulation Improve Diet Quality in Households with Children and Adolescents?
Source: Nutrients. 2019 Mar 14;11(3):618. doi: 10.3390/nu11030618 (PMC6470779; doi:10.3390/nu11030618)
Supplement: Supplementary file 1 [file nutrients-11-00618-s001.zip › Table S1 Detailed Version of Table 1.pdf]

Products

1	Cereal Bars
2	Cookies
3	Crackers
4	Frankfurters
5	Fruit Drinks
6	Ice Cream
7	Lunch Combinations
8	Macaroni & Cheese (RTE)
9	Macaroni & Cheese (Mixes)
10	Nut Butters
11	Pizza
12	Potato Chips
13	RTE Cereal
14	Soft Drinks
15	Tortilla Chips
16	Yeast Breads
17	Yogurt

Types and Frequencies of Reformulation: Cereal Bars

Calculations using InfoScan Data for UPCs with positive sales

Average per-serving nutrient values*	2012-2014					2012-2015				
	2012	2014	Change	% Change	p-value	2012	2015	Change	% Change	p-value
All UPCs										
**Calories**										
Average per-serving	142.56	142.79	0.23	0.16%	0.0757	143.18	143.35	0.17	0.12%	0.2518
Standard deviation	18.73	18.57				18.78	18.68			
**Saturated fat (grams)**										
Average per-serving	2.05	1.84	-0.22	-10.51%	0.0000	2.07	1.84	-0.22	-10.76%	0.0000
Standard deviation	1.28	1.27				1.29	1.28			
**Sugar (grams)****										
Average per-serving	9.50	9.52	0.02	0.18%	0.3193	9.58				
Standard deviation	3.76	3.77				3.76				
**Sodium (milligrams)**										
Average per-serving	101.27	101.65	0.39	0.38%	0.0021	101.49	101.94	0.44	0.44%	0.0027
Standard deviation	47.08	47.28				47.55	47.82			
**Dietary fiber (grams)**										
Average per-serving	2.51	2.51	0.00	-0.10%	0.4975	2.48	2.47	-0.01	-0.42%	0.0708
Standard deviation	1.94	1.94				1.95	1.93			
**No. of UPCs**	1,880					1,635				
Branded UPCs										
**Calories**										
Average per-serving	142.27	142.57	0.30	0.21%	0.0484	142.99	143.25	0.25	0.18%	0.1587
Standard deviation	18.97	18.76				19.09	18.95			
**Saturated fat (grams)**										
Average per-serving	2.14	1.95	-0.19	-8.91%	0.0000	2.15	1.96	-0.20	-9.09%	0.0000
Standard deviation	1.33	1.31				1.34	1.31			
**Sugar (grams)****										
Average per-serving	9.29	9.31	0.03	0.29%	0.1752	9.37				
Standard deviation	3.99	4.01				4.00				
**Sodium (milligrams)**										
Average per-serving	102.21	102.55	0.34	0.33%	0.0148	102.74	103.12	0.38	0.37%	0.0201
Standard deviation	49.60	49.82				50.21	50.50			
**Dietary fiber (grams)**										
Average per-serving	2.53	2.53	0.00	-0.12%	0.4975	2.51	2.50	-0.01	-0.50%	0.0708
Standard deviation	1.93	1.92				1.96	1.93			
**No. of UPCs**	1,576					1,370				
Private Label UPCs										
**Calories**										
Average per-serving	144.09	143.94	-0.15	-0.10%	0.0956	144.15	143.91	-0.24	-0.16%	0.0346
Standard deviation	17.41	17.56				17.07	17.25			
**Saturated fat (grams)**										
Average per-serving	1.63	1.28	-0.35	-21.35%	0.0000	1.60	1.25	-0.36	-22.35%	0.0000
Standard deviation	0.90	0.87				0.92	0.89			
**Sugar (grams)****										
Average per-serving	10.61	10.58	-0.03	-0.31%	0.2759	10.67				
Standard deviation	1.78	1.85				1.78				
**Sodium (milligrams)**										
Average per-serving	96.35	96.99	0.64	0.67%	0.0338	95.02	95.80	0.78	0.82%	0.0286
Standard deviation	30.40	30.60				29.51	29.71			
**Dietary fiber (grams)**										
Average per-serving	2.41	2.41	0.00	0.00%	0.4975	2.30	2.30	0.00	0.00%	0.0708
Standard deviation	2.03	2.03				1.91	1.91			
**No. of UPCs**	304					265				

*Serving = 35 grams

**Sugar data is unavailable for 2015

Types and Frequencies of Reformulation: **Cookies and Brownies**

Calculations using InfoScan Data for UPCs with positive sales

	2012-2014					2012-2015				
Average per-serving nutrient values*	2012	2014	Change	% Change	p-value	2012	2015	Change	% Change	p-value
All UPCs										
**Calories**										
Average per-serving	136.20	136.24	0.04	0.03%	0.2491	136.17	136.25	0.07	0.05%	0.1758
Standard deviation	18.91	18.80				18.89	18.86			
**Saturated fat (grams)**										
Average per-serving	2.52	2.34	-0.18	-7.00%	0.0000	2.52	2.35	-0.17	-6.90%	0.0000
Standard deviation	1.55	1.54				1.57	1.55			
**Sugar (grams)****										
Average per-serving	9.84	9.84	0.00	-0.02%	0.4224	9.82				
Standard deviation	3.29	3.29				3.29				
**Sodium (milligrams)**										
Average per-serving	92.13	92.12	-0.02	-0.02%	0.6276	92.31	92.29	-0.02	-0.02%	0.7289
Standard deviation	38.72	38.62				38.62	38.49			
**Dietary fiber (grams)**										
Average per-serving	0.71	0.71	0.00	-0.30%	0.0792	0.71	0.71	0.00	-0.28%	0.1932
Standard deviation	0.78	0.77				0.77	0.77			
**No. of UPCs**	3,809					3,360				
Branded UPCs										
**Calories**										
Average per-serving	136.19	136.27	0.08	0.06%	0.0705	136.23	136.34	0.10	0.08%	0.1497
Standard deviation	19.62	19.48				19.45	19.42			
**Saturated fat (grams)**										
Average per-serving	2.55	2.38	-0.17	-6.82%	0.0000	2.55	2.38	-0.17	-6.78%	0.0000
Standard deviation	1.61	1.58				1.63	1.60			
**Sugar (grams)****										
Average per-serving	9.68	9.68	0.00	-0.01%	0.8106	9.67				
Standard deviation	3.44	3.45				3.45				
**Sodium (milligrams)**										
Average per-serving	88.39	88.36	-0.03	-0.04%	0.5231	88.46	88.38	-0.08	-0.09%	0.2517
Standard deviation	38.95	38.80				39.17	38.99			
**Dietary fiber (grams)**										
Average per-serving	0.77	0.77	0.00	-0.29%	0.1695	0.76	0.76	0.00	-0.08%	0.7409
Standard deviation	0.84	0.83				0.83	0.83			
**No. of UPCs**	2,708					2,393				
Private Label UPCs										
**Calories**										
Average per-serving	136.20	136.14	-0.06	-0.04%	0.0811	136.03	136.03	0.01	0.00%	0.9029
Standard deviation	17.03	17.00				17.42	17.41			
**Saturated fat (grams)**										
Average per-serving	2.44	2.26	-0.18	-7.47%	0.0000	2.44	2.26	-0.18	-7.33%	0.0000
Standard deviation	1.42	1.42				1.42	1.42			
**Sugar (grams)****										
Average per-serving	10.26	10.25	-0.01	-0.06%	0.0461	10.21				
Standard deviation	2.81	2.81				2.82				
**Sodium (milligrams)**										
Average per-serving	101.43	101.45	0.02	0.02%	0.7214	101.82	101.92	0.10	0.10%	0.3217
Standard deviation	36.50	36.48				35.51	35.43			
**Dietary fiber (grams)**										
Average per-serving	0.57	0.57	0.00	-0.35%	0.1839	0.57	0.57	-0.01	-0.94%	0.0299
Standard deviation	0.58	0.58				0.58	0.58			
**No. of UPCs**	1,099					965				

*Serving = 30 grams

**Sugar data is unavailable for 2015

Types and Frequencies of Reformulation: Crackers

Calculations using InfoScan Data for UPCs with positive sales

Average per-serving nutrient values*	2012-2014					2012-2015				
	2012	2014	Change	% Change	p-value	2012	2015	Change	% Change	p-value
All UPCs										
**Calories**										
Average per-serving	129.94	129.95	0.01	0.01%	0.8548	129.55	129.50	-0.05	-0.04%	0.4569
Standard deviation	17.98	17.82				17.93	17.71			
**Saturated fat (grams)**										
Average per-serving	1.06	0.90	-0.16	-15.21%	0.0000	1.04	0.89	-0.16	-15.12%	0.0000
Standard deviation	1.19	1.09				1.22	1.12			
**Sugar (grams)****										
Average per-serving	2.12	2.12	0.00	-0.09%	0.3542	2.10				
Standard deviation	2.59	2.59				2.60				
**Sodium (milligrams)**										
Average per-serving	204.79	204.81	0.02	0.01%	0.7911	202.47	202.48	0.01	0.01%	0.9198
Standard deviation	100.72	100.63				101.24	100.98			
**Dietary fiber (grams)**										
Average per-serving	1.32	1.32	-0.01	-0.49%	0.0108	1.32	1.32	-0.01	-0.57%	0.0093
Standard deviation	1.20	1.19				1.18	1.17			
**No. of UPCs**	2,052					1,842				
Branded UPCs										
**Calories**										
Average per-serving	129.55	129.57	0.01	0.01%	0.8548	129.34	129.28	-0.06	-0.05%	0.4570
Standard deviation	18.64	18.44				18.49	18.20			
**Saturated fat (grams)**										
Average per-serving	1.10	0.95	-0.15	-14.00%	0.0000	1.10	0.94	-0.15	-14.04%	0.0000
Standard deviation	1.26	1.16				1.28	1.18			
**Sugar (grams)****										
Average per-serving	1.91	1.91	0.00	-0.13%	0.3542	1.87				
Standard deviation	2.37	2.37				2.36				
**Sodium (milligrams)**										
Average per-serving	202.93	202.96	0.03	0.01%	0.7911	201.48	201.51	0.02	0.01%	0.8869
Standard deviation	99.13	99.02				99.63	99.30			
**Dietary fiber (grams)**										
Average per-serving	1.42	1.41	-0.01	-0.57%	0.0143	1.40	1.39	-0.01	-0.61%	0.0184
Standard deviation	1.24	1.24				1.22	1.21			
**No. of UPCs**	1,550					1,418				
Private Label UPCs										
**Calories**										
Average per-serving	131.27	131.27	0.00	0.00%		130.37	130.37	0.00	0.00%	
Standard deviation	15.53	15.53				15.70	15.70			
**Saturated fat (grams)**										
Average per-serving	0.94	0.76	-0.18	-19.60%	0.0000	0.87	0.70	-0.17	-19.67%	0.0000
Standard deviation	0.94	0.81				0.96	0.83			
**Sugar (grams)****										
Average per-serving	2.78	2.78	0.00	0.00%		2.89				
Standard deviation	3.09	3.09				3.16				
**Sodium (milligrams)**										
Average per-serving	210.64	210.64	0.00	0.00%		205.89	205.87	-0.02	-0.01%	0.3179
Standard deviation	105.42	105.42				106.60	106.58			
**Dietary fiber (grams)**										
Average per-serving	1.04	1.04	0.00	-0.12%	0.3178	1.06	1.06	0.00	-0.36%	0.1703
Standard deviation	0.98	0.98				1.00	1.00			
**No. of UPCs**	501					423				

*Serving = 30 grams

**Sugar data is unavailable for 2015

Types and Frequencies of Reformulation: Frankfurters

Calculations using InfoScan Data for UPCs with positive sales

	2012-2014					2012-2015				
Average per-serving nutrient values*	2012	2014	Change	% Change	p-value	2012	2015	Change	% Change	p-value
All UPCs										
**Calories**										
Average per-serving	157.80	157.79	-0.01	-0.01%	0.3177	157.49	157.23	-0.26	-0.17%	0.2955
Standard deviation	27.06	27.05				27.11	26.48			
**Saturated fat (grams)**										
Average per-serving	4.99	4.84	-0.16	-3.11%	0.0000	4.98	4.81	-0.16	-3.30%	0.0000
Standard deviation	1.36	1.39				1.35	1.37			
**Sugar (grams)****										
Average per-serving	1.11	1.11	0.00	-0.16%	0.3177	1.05				
Standard deviation	1.06	1.05				1.03				
**Sodium (milligrams)**										
Average per-serving	539.03	539.03	0.00	0.00%	0.3177	538.69	538.69	0.00	0.00%	0.3178
Standard deviation	104.33	104.33				106.09	106.09			
**Dietary fiber (grams)**										
Average per-serving	0.04	0.04	0.00	0.00%		0.04	0.04	0.00	0.00%	
Standard deviation	0.27	0.27				0.26	0.26			
**No. of UPCs**	573					498				
Branded UPCs										
**Calories**										
Average per-serving	156.54	156.53	-0.01	-0.01%	0.3178	156.21	155.92	-0.29	-0.19%	0.2955
Standard deviation	27.94	27.93				27.94	27.23			
**Saturated fat (grams)**										
Average per-serving	4.93	4.78	-0.15	-3.05%	0.0000	4.91	4.75	-0.16	-3.30%	0.0000
Standard deviation	1.40	1.42				1.38	1.40			
**Sugar (grams)****										
Average per-serving	1.07	1.07	0.00	-0.19%	0.3178	1.04				
Standard deviation	1.02	1.02				1.00				
**Sodium (milligrams)**										
Average per-serving	535.21	535.20	0.00	0.00%	0.3178	537.74	537.74	0.00	0.00%	0.3179
Standard deviation	103.79	103.78				106.52	106.51			
**Dietary fiber (grams)**										
Average per-serving	0.05	0.05	0.00	0.00%		0.04	0.04	0.00	0.00%	
Standard deviation	0.29	0.29				0.28	0.28			
**No. of UPCs**	497					443				
Private Label UPCs										
**Calories**										
Average per-serving	167.18	167.18	0.00	0.00%		169.54	169.54	0.00	0.00%	
Standard deviation	17.92	17.92				14.62	14.62			
**Saturated fat (grams)**										
Average per-serving	5.44	5.24	-0.19	-3.58%	0.0000	5.60	5.41	-0.19	-3.40%	0.0002
Standard deviation	1.04	1.07				0.89	0.90			
**Sugar (grams)****										
Average per-serving	1.37	1.37	0.00	0.00%		1.17				
Standard deviation	1.26	1.26				1.31				
**Sodium (milligrams)**										
Average per-serving	564.32	564.32	0.00	0.00%		545.67	545.67	0.00	0.00%	
Standard deviation	107.25	107.25				106.12	106.12			
**Dietary fiber (grams)**										
Average per-serving	0.00	0.00	0.00			0.00	0.00	0.00		
Standard deviation	0.00	0.00				0.00	0.00			
**No. of UPCs**	73					52				

*Serving = 56 grams

**Sugar data is unavailable for 2015

Types and Frequencies of Reformulation: Fruit Drinks

Calculations using InfoScan Data for UPCs with positive sales

	2012-2014					2012-2015				
Average per-serving nutrient values*	2012	2014	Change	% Change	p-value	2012	2015	Change	% Change	p-value
All UPCs										
**Calories**										
Average per-serving	98.76	98.80	0.04	0.04%	0.0013	99.02	98.83	-0.19	-0.19%	0.0576
Standard deviation	40.96	40.99				40.31	40.48			
**Saturated fat (grams)**										
Average per-serving										
Standard deviation										
**Sugar (grams)****										
Average per-serving	23.50	23.51	0.01	0.04%	0.0012	23.57				
Standard deviation	10.02	10.03				9.91				
**Sodium (milligrams)**										
Average per-serving	34.37	34.38	0.01	0.04%	0.0013	34.27	34.17	-0.10	-0.29%	0.0493
Standard deviation	35.06	35.08				35.06	35.07			
**Dietary fiber (grams)**										
Average per-serving										
Standard deviation										
**No. of UPCs**	2,116					1,903				
Branded UPCs										
**Calories**										
Average per-serving	97.42	97.47	0.06	0.06%	0.0016	97.92	97.67	-0.25	-0.26%	0.0563
Standard deviation	40.75	40.79				39.69	39.92			
**Saturated fat (grams)**										
Average per-serving										
Standard deviation										
**Sugar (grams)****										
Average per-serving	22.86	22.87	0.01	0.05%	0.0015	23.00				
Standard deviation	9.85	9.86				9.66				
**Sodium (milligrams)**										
Average per-serving	32.48	32.49	0.02	0.06%	0.0015	32.16	32.03	-0.13	-0.41%	0.0487
Standard deviation	37.32	37.34				37.14	37.14			
**Dietary fiber (grams)**										
Average per-serving										
Standard deviation										
**No. of UPCs**	1,590					1,446				
Private Label UPCs										
**Calories**										
Average per-serving	102.81	102.82	0.00	0.00%	0.3178	102.52	102.52	0.00	0.00%	0.3178
Standard deviation	41.37	41.37				42.05	42.05			
**Saturated fat (grams)**										
Average per-serving										
Standard deviation										
**Sugar (grams)****										
Average per-serving	25.43	25.44	0.00	0.00%	0.3178	25.36				
Standard deviation	10.30	10.30				10.47				
**Sodium (milligrams)**										
Average per-serving	40.09	40.10	0.00	0.00%	0.3178	40.95	40.95	0.00	0.00%	0.3178
Standard deviation	26.32	26.32				26.42	26.42			
**Dietary fiber (grams)**										
Average per-serving										
Standard deviation										
**No. of UPCs**	526					457				

*Serving = 240 grams (8 oz)

**Sugar data is unavailable for 2015

Types and Frequencies of Reformulation: Ice Cream

Calculations using InfoScan Data for UPCs with positive sales

	2012-2014					2012-2015				
Average per-serving nutrient values*	2012	2014	Change	% Change	p-value	2012	2015	Change	% Change	p-value
All UPCs										
**Calories**										
Average per-serving	139.99	140.00	0.01	0.01%	0.8696	140.23	140.25	0.02	0.01%	0.7329
Standard deviation	38.73	38.67				38.81	38.75			
**Saturated fat (grams)**										
Average per-serving	4.05	3.92	-0.13	-3.31%	0.0000	4.08	3.94	-0.13	-3.26%	0.0000
Standard deviation	2.26	2.28				2.26	2.29			
**Sugar (grams)****										
Average per-serving	13.74	13.74	0.00	-0.03%	0.2374	13.69				
Standard deviation	3.22	3.21				3.25				
**Sodium (milligrams)**										
Average per-serving	57.99	57.97	-0.02	-0.03%	0.5658	57.88	57.87	-0.01	-0.02%	0.8084
Standard deviation	31.85	31.80				31.64	31.59			
**Dietary fiber (grams)**										
Average per-serving	0.52	0.52	0.00	0.09%	0.4768	0.53	0.53	0.00	0.18%	0.2515
Standard deviation	0.94	0.94				0.95	0.96			
**No. of UPCs**	4,390					3,924				
Branded UPCs										
**Calories**										
Average per-serving	141.40	141.42	0.02	0.01%	0.7074	141.27	141.32	0.04	0.03%	0.4372
Standard deviation	40.56	40.49				40.60	40.54			
**Saturated fat (grams)**										
Average per-serving	4.11	3.98	-0.13	-3.16%	0.0000	4.14	4.01	-0.13	-3.05%	0.0000
Standard deviation	2.38	2.40				2.37	2.40			
**Sugar (grams)****										
Average per-serving	13.66	13.65	0.00	-0.03%	0.4237	13.61				
Standard deviation	3.33	3.32				3.36				
**Sodium (milligrams)**										
Average per-serving	57.38	57.35	-0.03	-0.05%	0.5173	56.90	56.86	-0.03	-0.05%	0.5813
Standard deviation	32.67	32.59				32.12	32.05			
**Dietary fiber (grams)**										
Average per-serving	0.63	0.63	0.00	0.15%	0.3213	0.63	0.63	0.00	0.23%	0.2516
Standard deviation	1.05	1.05				1.06	1.06			
**No. of UPCs**	2,820					2,591				
Private Label UPCs										
**Calories**										
Average per-serving	137.45	137.44	-0.01	-0.01%	0.9251	138.19	138.17	-0.03	-0.02%	0.8291
Standard deviation	35.09	35.03				35.00	34.96			
**Saturated fat (grams)**										
Average per-serving	3.95	3.81	-0.14	-3.59%	0.0000	3.95	3.81	-0.15	-3.69%	0.0000
Standard deviation	2.04	2.06				2.04	2.06			
**Sugar (grams)****										
Average per-serving	13.89	13.89	0.00	-0.03%	0.1574	13.84				
Standard deviation	3.01	3.01				3.02				
**Sodium (milligrams)**										
Average per-serving	59.08	59.09	0.01	0.01%	0.5718	59.79	59.83	0.03	0.05%	0.1344
Standard deviation	30.30	30.30				30.61	30.60			
**Dietary fiber (grams)**										
Average per-serving	0.32	0.32	0.00	-0.14%	0.3175	0.33	0.33	0.00	0.00%	
Standard deviation	0.65	0.65				0.67	0.67			
**No. of UPCs**	1,569					1,332				

*Serving = 65 grams

**Sugar data is unavailable for 2015

Types and Frequencies of Reformulation: Lunch Combinations

Calculations using InfoScan Data for UPCs with positive sales

	2012-2014					2012-2015				
Average per-serving nutrient values*	2012	2014	Change	% Change	p-value	2012	2015	Change	% Change	p-value
All UPCs										
**Calories**										
Average per-serving	320.06	346.88	26.83	8.38%	0.0251	326.77	359.19	32.42	9.92%	0.0248
Standard deviation	116.89	113.99				102.35	95.72			
**Saturated fat (grams)**										
Average per-serving	5.60	5.87	0.28	4.95%	0.2459	6.00	6.40	0.40	6.63%	0.1650
Standard deviation	3.33	3.51				3.03	3.14			
**Sugar (grams)****										
Average per-serving	17.35	19.17	1.83	10.53%	0.0534	16.49				
Standard deviation	10.63	11.86				10.42				
**Sodium (milligrams)**										
Average per-serving	706.12	737.90	31.79	4.50%	0.1702	756.65	795.49	38.84	5.13%	0.1659
Standard deviation	330.20	313.98				311.79	284.50			
**Dietary fiber (grams)**										
Average per-serving	1.33	1.44	0.12	8.70%	0.0325	1.28	1.42	0.14	10.91%	0.0322
Standard deviation	1.00	1.07				0.98	1.07			
**No. of UPCs**	52					43				
Branded UPCs										
**Calories**										
Average per-serving	320.06	346.88	26.83	8.38%	0.0251	326.77	359.19	32.42	9.92%	0.0248
Standard deviation	116.89	113.99				102.35	95.72			
**Saturated fat (grams)**										
Average per-serving	5.60	5.87	0.28	4.95%	0.2459	6.00	6.40	0.40	6.63%	0.1650
Standard deviation	3.33	3.51				3.03	3.14			
**Sugar (grams)****										
Average per-serving	17.35	19.17	1.83	10.53%	0.0534	16.49				
Standard deviation	10.63	11.86				10.42				
**Sodium (milligrams)**										
Average per-serving	706.12	737.90	31.79	4.50%	0.1702	756.65	795.49	38.84	5.13%	0.1659
Standard deviation	330.20	313.98				311.79	284.50			
**Dietary fiber (grams)**										
Average per-serving	1.33	1.44	0.12	8.70%	0.0325	1.28	1.42	0.14	10.91%	0.0322
Standard deviation	1.00	1.07				0.98	1.07			
**No. of UPCs**	52					43				
Private Label UPCs										
**Calories**										
Average per-serving										
Standard deviation										
**Saturated fat (grams)**										
Average per-serving										
Standard deviation										
**Sugar (grams)****										
Average per-serving										
Standard deviation										
**Sodium (milligrams)**										
Average per-serving										
Standard deviation										
**Dietary fiber (grams)**										
Average per-serving										
Standard deviation										
**No. of UPCs**										

*Serving = 128 grams

**Sugar data is unavailable for 2015

Types and Frequencies of Reformulation: **Macaroni & Cheese (RTE)**

Calculations using InfoScan Data for UPCs with positive sales

	2012-2014					2012-2015				
Average per-serving nutrient values*	2012	2014	Change	% Change	p-value	2012	2015	Change	% Change	p-value
All UPCs										
**Calories**										
Average per-serving	337.45	337.19	-0.26	-0.08%	0.3219	337.57	337.28	-0.30	-0.09%	0.3225
Standard deviation	79.40	79.87				82.93	83.44			
**Saturated fat (grams)**										
Average per-serving	6.89	6.83	-0.06	-0.85%	0.1033	7.09	7.02	-0.07	-0.93%	0.1033
Standard deviation	4.21	4.25				4.32	4.37			
**Sugar (grams)****										
Average per-serving	4.28	4.28	0.00	0.00%		4.32				
Standard deviation	2.19	2.19				2.20				
**Sodium (milligrams)**										
Average per-serving	732.81	731.68	-1.13	-0.15%	0.3219	722.36	721.09	-1.28	-0.18%	0.3225
Standard deviation	242.04	239.97				235.94	233.48			
**Dietary fiber (grams)**										
Average per-serving	2.28	2.28	0.00	0.00%		2.26	2.26	0.00	0.00%	
Standard deviation	1.13	1.13				1.09	1.09			
**No. of UPCs**	53					47				
Branded UPCs										
**Calories**										
Average per-serving	329.14	328.82	-0.32	-0.10%	0.3229	329.10	328.74	-0.36	-0.11%	0.3236
Standard deviation	75.51	76.08				78.68	79.30			
**Saturated fat (grams)**										
Average per-serving	6.18	6.11	-0.07	-1.14%	0.1034	6.41	6.33	-0.08	-1.24%	0.1034
Standard deviation	3.43	3.48				3.51	3.57			
**Sugar (grams)****										
Average per-serving	4.14	4.14	0.00	0.00%		4.18				
Standard deviation	2.16	2.16				2.15				
**Sodium (milligrams)**										
Average per-serving	709.02	707.66	-1.36	-0.19%	0.3229	705.33	703.79	-1.54	-0.22%	0.3236
Standard deviation	239.64	236.97				240.71	237.67			
**Dietary fiber (grams)**										
Average per-serving	2.39	2.39	0.00	0.00%		2.36	2.36	0.00	0.00%	
Standard deviation	1.15	1.15				1.09	1.09			
**No. of UPCs**	44					39				
Private Label UPCs										
**Calories**										
Average per-serving	378.11	378.11	0.00	0.00%		378.88	378.88	0.00	0.00%	
Standard deviation	89.93	89.93				96.11	96.11			
**Saturated fat (grams)**										
Average per-serving	10.33	10.33	0.00	0.00%		10.38	10.38	0.00	0.00%	
Standard deviation	5.98	5.98				6.39	6.39			
**Sugar (grams)****										
Average per-serving	5.00	5.00	0.00	0.00%		5.00				
Standard deviation	2.29	2.29				2.45				
**Sodium (milligrams)**										
Average per-serving	849.11	849.11	0.00	0.00%		805.38	805.38	0.00	0.00%	
Standard deviation	231.89	231.89				204.40	204.40			
**Dietary fiber (grams)**										
Average per-serving	1.78	1.78	0.00	0.00%		1.75	1.75	0.00	0.00%	
Standard deviation	0.97	0.97				1.04	1.04			
**No. of UPCs**	9					8				

*Serving =227 grams

**Sugar data is unavailable for 2015

Types and Frequencies of Reformulation: Macaroni and Cheese (Mixes)

Calculations using InfoScan Data for UPCs with positive sales

	2012-2014					2012-2015				
Average per-serving nutrient values*	2012	2014	Change	% Change	p-value	2012	2015	Change	% Change	p-value
All UPCs										
**Calories**										
Average per-serving	243.82	243.95	0.12	0.05%	0.2952	243.83	243.92	0.09	0.04%	0.5111
Standard deviation	25.90	25.83				26.24	26.25			
**Saturated fat (grams)**										
Average per-serving	1.94	1.77	-0.17	-8.72%	0.0000	1.96	1.79	-0.17	-8.83%	0.0000
Standard deviation	1.18	1.18				1.19	1.19			
**Sugar (grams)****										
Average per-serving	4.66	4.67	0.00	0.06%	0.3180	4.67				
Standard deviation	2.32	2.33				2.34				
**Sodium (milligrams)**										
Average per-serving	590.16	590.45	0.29	0.05%	0.2933	588.64	588.90	0.26	0.04%	0.4061
Standard deviation	100.45	100.25				102.31	101.98			
**Dietary fiber (grams)**										
Average per-serving	1.61	1.62	0.00	0.18%	0.3180	1.61	1.61	0.00	0.19%	0.3181
Standard deviation	0.89	0.90				0.86	0.88			
**No. of UPCs**	345					319				
Branded UPCs										
**Calories**										
Average per-serving	243.41	243.69	0.28	0.12%	0.2961	243.44	243.71	0.26	0.11%	0.3597
Standard deviation	33.51	33.38				34.32	34.37			
**Saturated fat (grams)**										
Average per-serving	2.10	1.91	-0.19	-9.07%	0.0000	2.12	1.92	-0.19	-9.15%	0.0000
Standard deviation	0.98	0.90				0.99	0.92			
**Sugar (grams)****										
Average per-serving	5.05	5.06	0.01	0.13%	0.3189	5.05				
Standard deviation	2.13	2.14				2.13				
**Sodium (milligrams)**										
Average per-serving	570.11	570.76	0.65	0.11%	0.2941	567.08	567.86	0.78	0.14%	0.2361
Standard deviation	113.45	113.17				114.70	114.12			
**Dietary fiber (grams)**										
Average per-serving	1.67	1.68	0.01	0.39%	0.3189	1.65	1.66	0.01	0.42%	0.3190
Standard deviation	1.16	1.18				1.10	1.12			
**No. of UPCs**	153					144				
Private Label UPCs										
**Calories**										
Average per-serving	244.15	244.15	0.00	0.00%		244.15	244.10	-0.06	-0.02%	0.3187
Standard deviation	17.74	17.74				17.02	16.97			
**Saturated fat (grams)**										
Average per-serving	1.82	1.67	-0.15	-8.40%	0.0000	1.83	1.68	-0.16	-8.54%	0.0000
Standard deviation	1.31	1.36				1.32	1.37			
**Sugar (grams)****										
Average per-serving	4.35	4.35	0.00	0.00%		4.37				
Standard deviation	2.43	2.43				2.47				
**Sodium (milligrams)**										
Average per-serving	606.14	606.14	0.00	0.00%		606.38	606.21	-0.17	-0.03%	0.3187
Standard deviation	85.73	85.73				87.26	87.38			
**Dietary fiber (grams)**										
Average per-serving	1.57	1.57	0.00	0.00%		1.57	1.57	0.00	0.00%	
Standard deviation	0.59	0.59				0.60	0.60			
**No. of UPCs**	192					175				

*Serving = 70 grams

**Sugar data is unavailable for 2015

Types and Frequencies of Reformulation: Nut Butters

Calculations using InfoScan Data for UPCs with positive sales

	2012-2014					2012-2015				
Average per-serving nutrient values*	2012	2014	Change	% Change	p-value	2012	2015	Change	% Change	p-value
All UPCs										
**Calories**										
Average per-serving	188.62	188.72	0.10	0.05%	0.4673	188.60	188.62	0.03	0.01%	0.8695
Standard deviation	20.28	19.80				19.62	19.04			
**Saturated fat (grams)**										
Average per-serving	2.87	2.65	-0.22	-7.70%	0.0000	2.88	2.66	-0.23	-7.86%	0.0000
Standard deviation	0.87	0.78				0.86	0.78			
**Sugar (grams)****										
Average per-serving	3.65	3.65	0.00	0.08%	0.1639	3.64				
Standard deviation	3.59	3.59				3.56				
**Sodium (milligrams)**										
Average per-serving	100.87	100.87	0.00	0.00%	0.5828	101.24	101.23	-0.01	-0.01%	0.7068
Standard deviation	54.33	54.32				54.46	54.45			
**Dietary fiber (grams)**										
Average per-serving	2.17	2.17	0.00	-0.03%	0.7940	2.17	2.17	0.00	0.13%	0.3461
Standard deviation	0.78	0.78				0.79	0.78			
**No. of UPCs**	565					524				
Branded UPCs										
**Calories**										
Average per-serving	186.50	186.68	0.19	0.10%	0.4677	186.45	186.67	0.22	0.12%	0.4225
Standard deviation	22.91	22.16				21.85	20.96			
**Saturated fat (grams)**										
Average per-serving	2.62	2.42	-0.20	-7.68%	0.0000	2.64	2.43	-0.21	-7.91%	0.0000
Standard deviation	0.82	0.75				0.82	0.76			
**Sugar (grams)****										
Average per-serving	3.59	3.60	0.01	0.15%	0.1641	3.58				
Standard deviation	3.69	3.70				3.58				
**Sodium (milligrams)**										
Average per-serving	86.25	86.25	0.01	0.01%	0.5832	87.85	87.87	0.01	0.01%	0.4174
Standard deviation	56.17	56.15				56.23	56.21			
**Dietary fiber (grams)**										
Average per-serving	2.29	2.29	0.00	-0.06%	0.7942	2.28	2.28	0.00	0.08%	0.6868
Standard deviation	0.86	0.86				0.87	0.86			
**No. of UPCs**	311					288				
Private Label UPCs										
**Calories**										
Average per-serving	191.22	191.22	0.00	0.00%		191.16	190.95	-0.21	-0.11%	0.0957
Standard deviation	16.16	16.16				16.17	16.14			
**Saturated fat (grams)**										
Average per-serving	3.17	2.92	-0.24	-7.73%	0.0000	3.17	2.92	-0.25	-7.81%	0.0000
Standard deviation	0.83	0.72				0.82	0.73			
**Sugar (grams)****										
Average per-serving	3.72	3.72	0.00	0.00%		3.73				
Standard deviation	3.46	3.46				3.55				
**Sodium (milligrams)**										
Average per-serving	118.77	118.77	0.00	0.00%		117.82	117.78	-0.04	-0.04%	0.5648
Standard deviation	46.15	46.15				47.41	47.42			
**Dietary fiber (grams)**										
Average per-serving	2.02	2.02	0.00	0.00%		2.03	2.03	0.00	0.21%	0.3183
Standard deviation	0.63	0.63				0.65	0.64			
**No. of UPCs**	254					235				

*Serving = 32 grams

**Sugar data is unavailable for 2015

Types and Frequencies of Reformulation: Pizza

Calculations using InfoScan Data for UPCs with positive sales

	2012-2014					2012-2015				
Average per-serving nutrient values*	2012	2014	Change	% Change	p-value	2012	2015	Change	% Change	p-value
All UPCs										
**Calories**										
Average per-serving	337.43	337.05	-0.38	-0.11%	0.0373	337.91	337.46	-0.45	-0.13%	0.0336
Standard deviation	39.33	39.60				39.25	39.58			
**Saturated fat (grams)**										
Average per-serving	6.00	5.89	-0.11	-1.87%	0.0000	6.00	5.89	-0.11	-1.90%	0.0000
Standard deviation	2.10	2.15				2.01	2.06			
**Sugar (grams)****										
Average per-serving	4.20	4.20	-0.01	-0.19%	0.3322	4.20				
Standard deviation	2.64	2.62				2.52				
**Sodium (milligrams)**										
Average per-serving	731.16	731.36	0.20	0.03%	0.5526	737.02	737.23	0.21	0.03%	0.5862
Standard deviation	175.92	176.23				172.43	172.82			
**Dietary fiber (grams)**										
Average per-serving	2.31	2.31	0.00	-0.08%	0.5702	2.30	2.30	0.00	-0.10%	0.5274
Standard deviation	1.18	1.18				1.13	1.12			
**No. of UPCs**	990					852				
Branded UPCs										
**Calories**										
Average per-serving	340.83	340.83	0.00	0.00%	0.1575	341.42	341.41	-0.01	0.00%	0.4606
Standard deviation	40.51	40.51				40.24	40.26			
**Saturated fat (grams)**										
Average per-serving	6.18	6.09	-0.09	-1.39%	0.0000	6.18	6.09	-0.09	-1.44%	0.0000
Standard deviation	1.98	2.06				1.97	2.05			
**Sugar (grams)****										
Average per-serving	4.46	4.46	0.00	0.00%		4.38				
Standard deviation	2.81	2.81				2.58				
**Sodium (milligrams)**										
Average per-serving	739.40	739.41	0.00	0.00%	0.1799	740.35	740.32	-0.02	0.00%	0.4215
Standard deviation	176.02	176.01				174.78	174.82			
**Dietary fiber (grams)**										
Average per-serving	2.42	2.42	0.00	0.01%	0.3177	2.39	2.39	0.00	0.00%	
Standard deviation	1.32	1.32				1.24	1.24			
**No. of UPCs**	672					600				
Private Label UPCs										
**Calories**										
Average per-serving	330.26	329.08	-1.18	-0.36%	0.0361	329.55	328.06	-1.49	-0.45%	0.0361
Standard deviation	35.74	36.40				35.47	36.27			
**Saturated fat (grams)**										
Average per-serving	5.64	5.47	-0.17	-2.99%	0.0000	5.58	5.41	-0.18	-3.13%	0.0000
Standard deviation	2.29	2.29				2.04	2.01			
**Sugar (grams)****										
Average per-serving	3.67	3.65	-0.03	-0.68%	0.3328	3.76				
Standard deviation	2.14	2.07				2.30				
**Sodium (milligrams)**										
Average per-serving	713.75	714.35	0.60	0.08%	0.5593	729.09	729.85	0.76	0.10%	0.5595
Standard deviation	174.71	175.75				166.79	168.09			
**Dietary fiber (grams)**										
Average per-serving	2.08	2.07	-0.01	-0.30%	0.5279	2.09	2.08	-0.01	-0.38%	0.5282
Standard deviation	0.78	0.76				0.75	0.73			
**No. of UPCs**	318					252				

*Serving = 142 grams

**Sugar data is unavailable for 2015

Types and Frequencies of Reformulation: Potato Chips

Calculations using InfoScan Data for UPCs with positive sales

	2012-2014					2012-2015				
Average per-serving nutrient values*	2012	2014	Change	% Change	p-value	2012	2015	Change	% Change	p-value
All UPCs										
**Calories**										
Average per-serving	149.25	149.25	0.00	0.00%	0.9185	149.33	149.34	0.01	0.00%	0.7126
Standard deviation	12.21	12.23				11.65	11.68			
**Saturated fat (grams)**										
Average per-serving	2.03	1.84	-0.19	-9.47%	0.0000	2.06	1.86	-0.19	-9.43%	0.0000
Standard deviation	0.91	0.87				0.91	0.87			
**Sugar (grams)****										
Average per-serving	0.83	0.83	0.00	-0.15%	0.1574	0.82				
Standard deviation	0.91	0.91				0.91				
**Sodium (milligrams)**										
Average per-serving	195.90	196.02	0.12	0.06%	0.2090	194.38	194.49	0.11	0.05%	0.4129
Standard deviation	87.79	87.78				87.64	87.62			
**Dietary fiber (grams)**										
Average per-serving	1.03	1.03	0.00	-0.06%	0.1574	1.03	1.03	0.00	-0.07%	0.1574
Standard deviation	0.33	0.33				0.33	0.33			
**No. of UPCs**	1,610					1,473				
Branded UPCs										
**Calories**										
Average per-serving	149.11	149.11	0.00	0.00%	0.9592	149.11	149.11	0.01	0.00%	0.7401
Standard deviation	12.52	12.53				11.79	11.83			
**Saturated fat (grams)**										
Average per-serving	2.02	1.82	-0.20	-9.80%	0.0000	2.06	1.86	-0.20	-9.63%	0.0000
Standard deviation	0.91	0.86				0.91	0.86			
**Sugar (grams)****										
Average per-serving	0.87	0.87	0.00	-0.17%	0.1574	0.85				
Standard deviation	0.94	0.94				0.93				
**Sodium (milligrams)**										
Average per-serving	195.58	195.72	0.15	0.07%	0.2090	195.27	195.40	0.12	0.06%	0.4129
Standard deviation	88.62	88.61				88.85	88.82			
**Dietary fiber (grams)**										
Average per-serving	1.04	1.04	0.00	-0.07%	0.1574	1.04	1.04	0.00	-0.08%	0.1574
Standard deviation	0.30	0.30				0.31	0.31			
**No. of UPCs**	1,362					1,265				
Private Label UPCs										
**Calories**										
Average per-serving	150.04	150.05	0.00	0.00%	0.3183	150.69	150.69	0.00	0.00%	0.3185
Standard deviation	10.37	10.35				10.67	10.65			
**Saturated fat (grams)**										
Average per-serving	2.10	1.93	-0.16	-7.77%	0.0000	2.08	1.91	-0.17	-8.18%	0.0000
Standard deviation	0.95	0.93				0.97	0.95			
**Sugar (grams)****										
Average per-serving	0.65	0.65	0.00	0.00%		0.61				
Standard deviation	0.72	0.72				0.71				
**Sodium (milligrams)**										
Average per-serving	197.67	197.67	0.00	0.00%		188.96	188.96	0.00	0.00%	
Standard deviation	83.20	83.20				79.89	79.89			
**Dietary fiber (grams)**										
Average per-serving	0.97	0.97	0.00	0.00%		0.94	0.94	0.00	0.00%	
Standard deviation	0.44	0.44				0.44	0.44			
**No. of UPCs**	248					208				

*Serving = 28 grams

**Sugar data is unavailable for 2015

Types and Frequencies of Reformulation: RTE Cereal

Calculations using InfoScan Data for UPCs with positive sales

	2012-2014					2012-2015				
Average per-serving nutrient values*	2012	2014	Change	% Change	p-value	2012	2015	Change	% Change	p-value
All UPCs										
**Calories**										
Average per-serving	114.47	114.48	0.01	0.01%	0.5272	114.38	114.41	0.03	0.02%	0.1986
Standard deviation	11.56	11.55				11.69	11.68			
**Saturated fat (grams)**										
Average per-serving	0.24	0.17	-0.07	-30.51%	0.0000	0.25	0.17	-0.07	-30.14%	0.0000
Standard deviation	0.46	0.36				0.47	0.37			
**Sugar (grams)****										
Average per-serving	7.80	7.80	0.00	0.02%	0.5638	7.59				
Standard deviation	4.09	4.09				4.06				
**Sodium (milligrams)**										
Average per-serving	134.03	134.01	-0.02	-0.02%	0.6013	131.12	131.01	-0.12	-0.09%	0.2145
Standard deviation	76.70	76.68				77.88	77.82			
**Dietary fiber (grams)**										
Average per-serving	2.07	2.07	0.00	-0.13%	0.3305	2.12	2.11	0.00	-0.16%	0.3305
Standard deviation	1.68	1.68				1.63	1.63			
**No. of UPCs**	2,149					1,797				
Branded UPCs										
**Calories**										
Average per-serving	115.30	115.33	0.02	0.02%	0.4065	115.35	115.38	0.03	0.03%	0.3534
Standard deviation	11.44	11.44				11.68	11.65			
**Saturated fat (grams)**										
Average per-serving	0.33	0.24	-0.10	-28.81%	0.0000	0.35	0.25	-0.10	-28.37%	0.0000
Standard deviation	0.51	0.41				0.52	0.42			
**Sugar (grams)****										
Average per-serving	7.88	7.88	0.00	0.03%	0.5639	7.80				
Standard deviation	3.82	3.82				3.83				
**Sodium (milligrams)**										
Average per-serving	117.46	117.37	-0.10	-0.08%	0.1116	115.39	115.29	-0.10	-0.09%	0.1542
Standard deviation	75.28	75.20				74.81	74.73			
**Dietary fiber (grams)**										
Average per-serving	2.24	2.23	-0.01	-0.28%	0.2500	2.26	2.25	-0.01	-0.32%	0.2500
Standard deviation	1.57	1.56				1.55	1.55			
**No. of UPCs**	1,102					968				
Private Label UPCs										
**Calories**										
Average per-serving	113.54	113.53	-0.01	-0.01%	0.1798	113.21	113.22	0.02	0.02%	0.2651
Standard deviation	11.55	11.54				11.53	11.55			
**Saturated fat (grams)**										
Average per-serving	0.13	0.09	-0.05	-35.68%	0.0000	0.13	0.08	-0.05	-36.38%	0.0000
Standard deviation	0.37	0.28				0.36	0.26			
**Sugar (grams)****										
Average per-serving	7.72	7.72	0.00	0.00%		7.36				
Standard deviation	4.35	4.35				4.31				
**Sodium (milligrams)**										
Average per-serving	151.68	151.73	0.06	0.04%	0.3590	149.67	149.53	-0.14	-0.10%	0.4561
Standard deviation	74.22	74.23				77.32	77.29			
**Dietary fiber (grams)**										
Average per-serving	1.89	1.90	0.00	0.05%	0.3175	1.95	1.95	0.00	0.06%	0.3176
Standard deviation	1.78	1.78				1.70	1.70			
**No. of UPCs**	1,045					828				

*Serving = 30 grams

**Sugar data is unavailable for 2015

Types and Frequencies of Reformulation: Soft Drinks

Calculations using InfoScan Data for UPCs with positive sales

	2012-2014					2012-2015				
Average per-serving nutrient values*	2012	2014	Change	% Change	p-value	2012	2015	Change	% Change	p-value
All UPCs										
**Calories**										
Average per-serving	110.92	110.92	-0.01	0.00%	0.7416	111.01	110.95	-0.06	-0.05%	0.1403
Standard deviation	22.97	22.99				23.13	23.23			
**Saturated fat (grams)**										
Average per-serving										
Standard deviation										
**Sugar (grams)****										
Average per-serving	28.63	28.63	0.00	0.00%	0.9114	28.65				
Standard deviation	6.18	6.19				6.27				
**Sodium (milligrams)**										
Average per-serving	27.27	27.27	0.00	-0.01%	0.5159	27.23	27.21	-0.02	-0.08%	0.1025
Standard deviation	16.80	16.80				16.96	16.96			
**Dietary fiber (grams)**										
Average per-serving										
Standard deviation										
**No. of UPCs**	2,276					2,182				
Branded UPCs										
**Calories**										
Average per-serving	109.82	109.82	-0.01	-0.01%	0.7417	109.93	109.85	-0.08	-0.07%	0.1404
Standard deviation	23.39	23.43				23.47	23.60			
**Saturated fat (grams)**										
Average per-serving										
Standard deviation										
**Sugar (grams)****										
Average per-serving	28.36	28.36	0.00	0.00%	0.9114	28.39				
Standard deviation	6.33	6.34				6.40				
**Sodium (milligrams)**										
Average per-serving	29.62	29.61	0.00	-0.02%	0.5159	29.52	29.49	-0.03	-0.11%	0.1025
Standard deviation	16.81	16.81				16.93	16.94			
**Dietary fiber (grams)**										
Average per-serving										
Standard deviation										
**No. of UPCs**	1,662					1,603				
Private Label UPCs										
**Calories**										
Average per-serving	114.06	114.06	0.00	0.00%		114.17	114.17	0.00	0.00%	
Standard deviation	21.64	21.64				22.02	22.02			
**Saturated fat (grams)**										
Average per-serving										
Standard deviation										
**Sugar (grams)****										
Average per-serving	29.41	29.41	0.00	0.00%		29.40				
Standard deviation	5.71	5.71				5.83				
**Sodium (milligrams)**										
Average per-serving	20.81	20.81	0.00	0.00%		20.79	20.79	0.00	0.00%	
Standard deviation	15.27	15.27				15.59	15.59			
**Dietary fiber (grams)**										
Average per-serving										
Standard deviation										
**No. of UPCs**	596					561				

*Serving = 240 grams

**Sugar data is unavailable for 2015

Types and Frequencies of Reformulation: Tortilla & Other Chips

Calculations using InfoScan Data for UPCs with positive sales

	2012-2014					2012-2015				
Average per-serving nutrient values*	2012	2014	Change	% Change	p-value	2012	2015	Change	% Change	p-value
All UPCs										
**Calories**										
Average per-serving	141.84	141.82	-0.02	-0.01%	0.4497	142.02	142.01	-0.01	-0.01%	0.6900
Standard deviation	16.05	16.04				16.21	16.19			
**Saturated fat (grams)**										
Average per-serving	1.72	1.49	-0.23	-13.33%	0.0000	1.76	1.52	-0.24	-13.50%	0.0000
Standard deviation	1.36	1.26				1.39	1.28			
**Sugar (grams)****										
Average per-serving	0.74	0.74	0.00	0.00%	1.0000	0.73				
Standard deviation	1.20	1.20				1.22				
**Sodium (milligrams)**										
Average per-serving	215.46	215.52	0.07	0.03%	0.4344	222.27	222.47	0.20	0.09%	0.0991
Standard deviation	185.51	185.45				195.00	194.85			
**Dietary fiber (grams)**										
Average per-serving	1.35	1.34	-0.01	-0.54%	0.0001	1.33	1.32	-0.01	-0.44%	0.0190
Standard deviation	0.92	0.91				0.94	0.93			
**No. of UPCs**	1,476					1,235				
Branded UPCs										
**Calories**										
Average per-serving	142.02	142.01	-0.01	-0.01%	0.6911	142.29	142.29	-0.01	0.00%	0.8290
Standard deviation	16.41	16.39				16.55	16.53			
**Saturated fat (grams)**										
Average per-serving	1.80	1.56	-0.24	-13.27%	0.0000	1.83	1.59	-0.25	-13.42%	0.0000
Standard deviation	1.39	1.30				1.41	1.32			
**Sugar (grams)****										
Average per-serving	0.74	0.74	0.00	0.00%	1.0000	0.72				
Standard deviation	1.12	1.12				1.13				
**Sodium (milligrams)**										
Average per-serving	223.29	223.38	0.09	0.04%	0.3851	227.79	227.89	0.11	0.05%	0.3804
Standard deviation	192.06	191.98				200.87	200.77			
**Dietary fiber (grams)**										
Average per-serving	1.36	1.35	-0.01	-0.49%	0.0012	1.34	1.33	-0.01	-0.65%	0.0007
Standard deviation	0.92	0.92				0.94	0.94			
**No. of UPCs**	1,225					1,057				
Private Label UPCs										
**Calories**										
Average per-serving	140.97	140.92	-0.05	-0.03%	0.0140	140.42	140.38	-0.03	-0.02%	0.0833
Standard deviation	14.21	14.26				13.94	13.95			
**Saturated fat (grams)**										
Average per-serving	1.33	1.15	-0.18	-13.68%	0.0000	1.35	1.16	-0.19	-14.15%	0.0000
Standard deviation	1.11	0.99				1.13	0.99			
**Sugar (grams)****										
Average per-serving	0.76	0.76	0.00	0.00%		0.82				
Standard deviation	1.53	1.53				1.66				
**Sodium (milligrams)**										
Average per-serving	177.21	177.17	-0.04	-0.02%	0.0275	189.52	190.26	0.74	0.39%	0.0784
Standard deviation	143.81	143.81				152.10	151.58			
**Dietary fiber (grams)**										
Average per-serving	1.32	1.31	-0.01	-0.75%	0.0251	1.26	1.27	0.01	0.89%	0.1579
Standard deviation	0.88	0.87				0.92	0.92			
**No. of UPCs**	251					178				

*Serving = 28 grams

**Sugar data is unavailable for 2015

Types and Frequencies of Reformulation: Yeast Breads

Calculations using InfoScan Data for UPCs with positive sales

Average per-serving nutrient values*	2012-2014					2012-2015				
	2012	2014	Change	% Change	p-value	2012	2015	Change	% Change	p-value
All UPCs										
**Calories**										
Average per-serving	144.94	144.96	0.02	0.01%	0.3584	145.09	145.13	0.04	0.03%	0.1417
Standard deviation	20.33	20.31				20.35	20.32			
**Saturated fat (grams)**										
Average per-serving	0.17	0.14	-0.04	-21.15%	0.0000	0.17	0.14	-0.04	-20.75%	0.0000
Standard deviation	0.69	0.63				0.70	0.64			
**Sugar (grams)****										
Average per-serving	3.04	3.04	0.00	-0.02%	0.6875	3.01				
Standard deviation	2.72	2.72				2.73				
**Sodium (milligrams)**										
Average per-serving	281.08	281.03	-0.04	-0.02%	0.3010	281.74	281.70	-0.04	-0.01%	0.4602
Standard deviation	68.44	68.41				69.40	69.39			
**Dietary fiber (grams)**										
Average per-serving	2.37	2.37	0.00	-0.17%	0.0088	2.37	2.37	-0.01	-0.21%	0.0143
Standard deviation	1.86	1.86				1.86	1.86			
**No. of UPCs**	2,683					2,473				
Branded UPCs										
**Calories**										
Average per-serving	144.31	144.34	0.03	0.02%	0.1526	144.53	144.57	0.04	0.03%	0.0945
Standard deviation	21.44	21.43				21.33	21.32			
**Saturated fat (grams)**										
Average per-serving	0.18	0.14	-0.04	-20.00%	0.0000	0.17	0.14	-0.03	-19.49%	0.0000
Standard deviation	0.63	0.56				0.63	0.57			
**Sugar (grams)****										
Average per-serving	3.08	3.08	0.00	-0.03%	0.4633	3.04				
Standard deviation	2.71	2.71				2.69				
**Sodium (milligrams)**										
Average per-serving	273.88	273.88	-0.01	0.00%	0.9220	274.88	274.89	0.01	0.00%	0.8236
Standard deviation	73.18	73.14				73.95	73.90			
**Dietary fiber (grams)**										
Average per-serving	2.58	2.58	-0.01	-0.20%	0.0144	2.57	2.57	0.00	-0.19%	0.0380
Standard deviation	1.94	1.94				1.92	1.92			
**No. of UPCs**	1,680					1,562				
Private Label UPCs										
**Calories**										
Average per-serving	146.01	146.01	0.00	0.00%	0.9823	146.05	146.09	0.03	0.02%	0.5596
Standard deviation	18.29	18.24				18.52	18.47			
**Saturated fat (grams)**										
Average per-serving	0.16	0.13	-0.04	-23.21%	0.0000	0.17	0.13	-0.04	-22.97%	0.0000
Standard deviation	0.79	0.73				0.82	0.76			
**Sugar (grams)****										
Average per-serving	2.96	2.96	0.00	0.00%	1.0000	2.95				
Standard deviation	2.74	2.73				2.78				
**Sodium (milligrams)**										
Average per-serving	293.12	293.01	-0.11	-0.04%	0.1081	293.49	293.36	-0.13	-0.05%	0.2157
Standard deviation	57.73	57.75				59.04	59.15			
**Dietary fiber (grams)**										
Average per-serving	2.02	2.01	0.00	-0.10%	0.3176	2.03	2.03	-0.01	-0.27%	0.1656
Standard deviation	1.65	1.65				1.69	1.69			
**No. of UPCs**	1,003					910				

*Serving = 57 grams

**Sugar data is unavailable for 2015

Types and Frequencies of Reformulation: Yogurt

Calculations using InfoScan Data for UPCs with positive sales

	2012-2014					2012-2015				
Average per-serving nutrient values*	2012	2014	Change	% Change	p-value	2012	2015	Change	% Change	p-value
All UPCs										
**Calories**										
Average per-serving	137.06	137.27	0.21	0.15%	0.0203	137.45	137.64	0.19	0.14%	0.2313
Standard deviation	42.60	42.56				43.88	44.02			
**Saturated fat (grams)**										
Average per-serving	1.09	1.02	-0.07	-6.09%	0.0000	1.16	1.09	-0.07	-6.11%	0.0000
Standard deviation	1.80	1.75				1.87	1.82			
**Sugar (grams)****										
Average per-serving	18.93	18.95	0.02	0.09%	0.3062	18.91				
Standard deviation	8.33	8.35				8.51				
**Sodium (milligrams)**										
Average per-serving	96.94	97.00	0.06	0.06%	0.0769	97.28	97.39	0.11	0.11%	0.0415
Standard deviation	24.23	24.26				24.96	24.98			
**Dietary fiber (grams)**										
Average per-serving	0.31	0.32	0.01	3.22%	0.0005	0.30	0.31	0.01	3.63%	0.0016
Standard deviation	0.76	0.77				0.76	0.76			
**No. of UPCs**	1,385					1,183				
Branded UPCs										
**Calories**										
Average per-serving	144.06	144.41	0.35	0.25%	0.0203	144.60	144.83	0.23	0.16%	0.3551
Standard deviation	42.71	42.59				44.15	44.31			
**Saturated fat (grams)**										
Average per-serving	1.46	1.38	-0.08	-5.41%	0.0000	1.55	1.47	-0.08	-5.41%	0.0000
Standard deviation	2.19	2.14				2.28	2.23			
**Sugar (grams)****										
Average per-serving	19.59	19.62	0.03	0.14%	0.3064	19.48				
Standard deviation	7.80	7.83				8.04				
**Sodium (milligrams)**										
Average per-serving	94.06	94.17	0.10	0.11%	0.0769	93.84	93.97	0.13	0.14%	0.0738
Standard deviation	24.19	24.25				25.10	25.19			
**Dietary fiber (grams)**										
Average per-serving	0.30	0.32	0.02	5.67%	0.0004	0.29	0.31	0.02	6.40%	0.0016
Standard deviation	0.79	0.79				0.75	0.76			
**No. of UPCs**	817					701				
Private Label UPCs										
**Calories**										
Average per-serving	127.00	127.00	0.00	0.00%		127.06	127.18	0.12	0.10%	0.3178
Standard deviation	40.41	40.41				41.39	41.46			
**Saturated fat (grams)**										
Average per-serving	0.54	0.49	-0.05	-8.73%	0.0000	0.60	0.55	-0.05	-8.75%	0.0000
Standard deviation	0.72	0.63				0.74	0.64			
**Sugar (grams)****										
Average per-serving	17.99	17.99	0.00	0.00%		18.09				
Standard deviation	8.96	8.96				9.10				
**Sodium (milligrams)**										
Average per-serving	101.07	101.07	0.00	0.00%		102.29	102.37	0.08	0.08%	0.3178
Standard deviation	23.70	23.70				23.91	23.83			
**Dietary fiber (grams)**										
Average per-serving	0.33	0.33	0.00	0.00%		0.32	0.32	0.00	0.00%	
Standard deviation	0.73	0.73				0.76	0.76			
**No. of UPCs**	568					482				

*Serving = 170 grams

**Sugar data is unavailable for 2015
