# Supplementary material for: How Much Can Product Reformulation Improve Diet Quality in Households with Children and Adolescents?
Source: Nutrients. 2019 Mar 14;11(3):618. doi: 10.3390/nu11030618 (PMC6470779; doi:10.3390/nu11030618)
Supplement: Supplementary file 1 [file nutrients-11-00618-s001.zip › Table S2 Detailed Version of Table 2.pdf]

Products

1. Cereal Bars
2. Cookies
3. Crackers
4. Frankfurters
5. Fruit Drinks
6. Ice Cream
7. Lunch Combinations
8. Macaroni & Cheese (RTE)
9. Macaroni & Cheese (Mixes)
10. Nut Butters
11. Pizza
12. Potato Chips
13. RTE Cereal
14. Soft Drinks
15. Tortilla Chips
16. Yeast Breads
17. Yogurt

Types and Frequencies of Reformulation: Cereal Bars

Calculations using InfoScan Data for UPCs with positive sales

Average per-serving nutrient values*	National	Private Label	2014 Difference	% Difference	p-value
All UPCs					
**Calories**					
Average per-serving	142.73	144.13	1.40	0.97%	0.1969
Standard deviation	18.83	17.43			
**Saturated fat (grams)**					
Average per-serving	1.96	1.41	-0.55	-32.46%	0.0000
Standard deviation	1.28	1.54			
**Sugar (grams)**					
Average per-serving	9.17	10.52	1.35	13.70%	0.0000
Standard deviation	4.04	2.17			
**Sodium (milligrams)**					
Average per-serving	105.22	97.88	-7.33	-7.22%	0.0074
Standard deviation	49.22	32.03			
**Dietary fiber (grams)**					
Average per-serving	2.64	2.36	-0.28	-11.19%	0.0185
Standard deviation	2.06	1.98			
**No. of UPCs**	2,112	346			

*Serving = 35 grams

Types and Frequencies of Reformulation: Cookies and Brownies

Calculations using InfoScan Data for UPCs with positive sales

Average per-serving nutrient values*	National	Private Label	2014 Difference	% Difference	p-value
All UPCs					
**Calories**					
Average per-serving	136.39	136.05	-0.34	-0.25%	0.5680
Standard deviation	19.03	16.78			
**Saturated fat (grams)**					
Average per-serving	2.42	2.32	-0.09	-3.98%	0.0562
Standard deviation	1.56	1.44			
**Sugar (grams)**					
Average per-serving	9.77	10.21	0.44	4.39%	0.0000
Standard deviation	3.38	2.80			
**Sodium (milligrams)**					
Average per-serving	88.61	99.66	11.05	11.74%	0.0000
Standard deviation	38.29	37.11			
**Dietary fiber (grams)**					
Average per-serving	0.78	0.58	-0.20	-29.06%	0.0000
Standard deviation	0.87	0.71			
**No. of UPCs**	3,269	1,344			

*Serving = 30 grams

Types and Frequencies of Reformulation: Crackers

Calculations using InfoScan Data for UPCs with positive sales

Average per-serving nutrient values*	National	Private Label	2014 Difference	% Difference	p-value
All UPCs					
**Calories**					
Average per-serving	131.34	131.47	0.13	0.10%	0.8782
Standard deviation	18.46	17.05			
**Saturated fat (grams)**					
Average per-serving	0.99	0.78	-0.22	-24.51%	0.0000
Standard deviation	1.18	0.89			
**Sugar (grams)**					
Average per-serving	2.01	2.66	0.65	27.79%	0.0000
Standard deviation	2.41	3.03			
**Sodium (milligrams)**					
Average per-serving	207.59	214.35	6.76	3.20%	0.1581
Standard deviation	99.33	106.30			
**Dietary fiber (grams)**					
Average per-serving	1.37	1.00	-0.37	-31.04%	0.0000
Standard deviation	1.17	0.98			
**No. of UPCs**	1,901	582			

*Serving = 30 grams

Types and Frequencies of Reformulation: Frankfurters

Calculations using InfoScan Data for UPCs with positive sales

Average per-serving nutrient values*	National	Private Label	2014 Difference	% Difference	p-value
All UPCs					
**Calories**					
Average per-serving	156.42	168.09	11.67	7.19%	0.0002
Standard deviation	28.42	19.47			
**Saturated fat (grams)**					
Average per-serving	4.83	5.20	0.38	7.53%	0.0220
Standard deviation	1.46	1.15			
**Sugar (grams)**					
Average per-serving	1.03	1.49	0.46	36.73%	0.0001
Standard deviation	0.98	1.32			
**Sodium (milligrams)**					
Average per-serving	532.70	570.98	38.28	6.94%	0.0012
Standard deviation	100.05	115.80			
**Dietary fiber (grams)**					
Average per-serving	0.05	0.00	-0.05	-200.00%	0.1217
Standard deviation	0.27	0.00			
**No. of UPCs**	604	86			

*Serving = 56 grams

Types and Frequencies of Reformulation: Fruit Drinks

Calculations using InfoScan Data for UPCs with positive sales

Average per-serving nutrient values*	National	Private Label	2014 Difference	% Difference	p-value
All UPCs					
**Calories**					
Average per-serving	92.89	101.68	8.79	9.04%	0.0000
Standard deviation	41.00	40.82			
**Saturated fat (grams)**					
Average per-serving					
Standard deviation					
**Sugar (grams)**					
Average per-serving	21.82	25.07	3.25	13.87%	0.0000
Standard deviation	9.92	10.15			
**Sodium (milligrams)**					
Average per-serving	32.59	39.71	7.12	19.69%	0.0000
Standard deviation	37.92	26.60			
**Dietary fiber (grams)**					
Average per-serving					
Standard deviation					
**No. of UPCs**	2,059	630			

*Serving = 240 grams (8 oz)

Types and Frequencies of Reformulation: Ice Cream

Calculations using InfoScan Data for UPCs with positive sales

Average per-serving nutrient values*	National	Private Label	2014 Difference	% Difference	p-value
All UPCs					
**Calories**					
Average per-serving	141.48	137.84	-3.64	-2.61%	0.0014
Standard deviation	40.83	35.06			
**Saturated fat (grams)**					
Average per-serving	3.98	3.84	-0.14	-3.54%	0.0412
Standard deviation	2.43	2.09			
**Sugar (grams)**					
Average per-serving	13.77	13.92	0.15	1.11%	0.1030
Standard deviation	3.34	2.98			
**Sodium (milligrams)**					
Average per-serving	57.24	59.20	1.96	3.37%	0.0340
Standard deviation	32.45	30.08			
**Dietary fiber (grams)**					
Average per-serving	0.63	0.32	-0.31	-64.80%	0.0000
Standard deviation	1.03	0.64			
**No. of UPCs**	3,345	1,802			

*Serving = 65 grams

Types and Frequencies of Reformulation: Lunch Combinations

Calculations using InfoScan Data for UPCs with positive sales

Average per-serving nutrient values*	National	Private Label	2014<br>Difference	% Difference	p-value
All UPCs					
**Calories**					
Average per-serving	No private label products				
Standard deviation					
**Saturated fat (grams)**					
Average per-serving					
Standard deviation					
**Sugar (grams)**					
Average per-serving					
Standard deviation					
**Sodium (milligrams)**					
Average per-serving					
Standard deviation					
**Dietary fiber (grams)**					
Average per-serving					
Standard deviation					
**No. of UPCs**					

*Serving = 128 grams

Types and Frequencies of Reformulation: Macaroni & Cheese (RTE)

Calculations using InfoScan Data for UPCs with positive sales

Average per-serving nutrient values*	National	Private Label	2014 Difference	% Difference	p-value
All UPCs					
**Calories**					
Average per-serving	334.99	360.43	25.44	7.32%	0.2202
Standard deviation	68.09	79.97			
**Saturated fat (grams)**					
Average per-serving	7.06	9.50	2.44	29.51%	0.0424
Standard deviation	3.80	5.06			
**Sugar (grams)**					
Average per-serving	4.28	4.71	0.43	9.67%	0.4798
Standard deviation	2.06	2.23			
**Sodium (milligrams)**					
Average per-serving	733.15	821.93	88.78	11.42%	0.1654
Standard deviation	218.16	205.20			
**Dietary fiber (grams)**					
Average per-serving	2.24	1.79	-0.45	-22.36%	0.1923
Standard deviation	1.22	0.80			
**No. of UPCs**	68	14			

*Serving =227 grams

Types and Frequencies of Reformulation: **Macaroni and Cheese (Mixes)**

Calculations using InfoScan Data for UPCs with positive sales

Average per-serving nutrient values*	National	Private Label	2014 Difference	% Difference	p-value
All UPCs					
**Calories**					
Average per-serving	245.92	244.68	-1.24	-0.50%	0.6123
Standard deviation	30.77	17.49			
**Saturated fat (grams)**					
Average per-serving	1.88	1.64	-0.24	-13.76%	0.0400
Standard deviation	0.99	1.35			
**Sugar (grams)**					
Average per-serving	5.04	4.39	-0.64	-13.62%	0.0036
Standard deviation	2.01	2.39			
**Sodium (milligrams)**					
Average per-serving	572.18	604.93	32.75	5.56%	0.0005
Standard deviation	104.47	84.68			
**Dietary fiber (grams)**					
Average per-serving	1.65	1.56	-0.09	-5.38%	0.3069
Standard deviation	1.08	0.58			
**No. of UPCs**	195	216			

*Serving = 70 grams

Types and Frequencies of Reformulation: Nut Butters

Calculations using InfoScan Data for UPCs with positive sales

Average per-serving nutrient values*	National	Private Label	2014 Difference	% Difference	p-value
All UPCs					
**Calories**					
Average per-serving	186.16	190.07	3.91	2.08%	0.0080
Standard deviation	21.98	16.22			
**Saturated fat (grams)**					
Average per-serving	2.47	2.89	0.42	15.58%	0.0000
Standard deviation	0.83	0.73			
**Sugar (grams)**					
Average per-serving	4.03	3.88	-0.16	-3.94%	0.5928
Standard deviation	4.04	3.71			
**Sodium (milligrams)**					
Average per-serving	88.58	116.08	27.49	26.87%	0.0000
Standard deviation	56.88	47.62			
**Dietary fiber (grams)**					
Average per-serving	2.25	2.00	-0.25	-11.87%	0.0000
Standard deviation	0.89	0.64			
**No. of UPCs**	406	319			

*Serving = 32 grams

Types and Frequencies of Reformulation: Pizza

Calculations using InfoScan Data for UPCs with positive sales

Average per-serving nutrient values*	National	Private Label	2014 Difference	% Difference	p-value
All UPCs					
**Calories**					
Average per-serving	340.77	330.37	-10.40	-3.10%	0.0000
Standard deviation	42.56	34.70			
**Saturated fat (grams)**					
Average per-serving	6.11	5.52	-0.59	-10.15%	0.0000
Standard deviation	2.02	2.06			
**Sugar (grams)**					
Average per-serving	4.46	3.69	-0.77	-18.78%	0.0000
Standard deviation	2.73	2.24			
**Sodium (milligrams)**					
Average per-serving	733.87	733.05	-0.82	-0.11%	0.9289
Standard deviation	173.15	164.39			
**Dietary fiber (grams)**					
Average per-serving	2.38	2.05	-0.33	-15.07%	0.0000
Standard deviation	1.29	0.75			
**No. of UPCs**	937	538			

*Serving = 142 grams

Types and Frequencies of Reformulation: Potato Chips

Calculations using InfoScan Data for UPCs with positive sales

			2014		
Average per-serving nutrient values*	National	Private Label	Difference	% Difference	p-value
All UPCs					
**Calories**					
Average per-serving	149.64	151.04	1.40	0.93%	0.0516
Standard deviation	12.20	10.78			
**Saturated fat (grams)**					
Average per-serving	1.76	1.84	0.08	4.33%	0.1248
Standard deviation	0.84	0.89			
**Sugar (grams)**					
Average per-serving	0.89	0.71	-0.18	-22.10%	0.0008
Standard deviation	0.91	0.72			
**Sodium (milligrams)**					
Average per-serving	192.68	194.62	1.94	1.00%	0.6998
Standard deviation	85.15	78.79			
**Dietary fiber (grams)**					
Average per-serving	1.04	0.98	-0.06	-5.69%	0.0022
Standard deviation	0.29	0.39			
**No. of UPCs**	1,643	336			

*Serving = 28 grams

Types and Frequencies of Reformulation: RTE Cereal

Calculations using InfoScan Data for UPCs with positive sales

			2014		
Average per-serving nutrient values*	National	Private Label	Difference	% Difference	p-value
All UPCs					
**Calories**					
Average per-serving	116.18	113.70	-2.48	-2.16%	0.0000
Standard deviation	12.13	11.22			
**Saturated fat (grams)**					
Average per-serving	0.28	0.08	-0.19	-108.41%	0.0000
Standard deviation	0.49	0.27			
**Sugar (grams)**					
Average per-serving	8.09	7.87	-0.22	-2.72%	0.1585
Standard deviation	3.74	4.33			
**Sodium (milligrams)**					
Average per-serving	119.20	150.33	31.13	23.10%	0.0000
Standard deviation	72.31	72.65			
**Dietary fiber (grams)**					
Average per-serving	2.17	1.89	-0.28	-13.63%	0.0000
Standard deviation	1.52	1.74			
**No. of UPCs**	1,500	1,243			

*Serving = 30 grams

Types and Frequencies of Reformulation: Soft Drinks

Calculations using InfoScan Data for UPCs with positive sales

Average per-serving nutrient values*	National	Private Label	2014 Difference	% Difference	p-value
All UPCs					
**Calories**					
Average per-serving	108.83	113.71	4.88	4.39%	0.0000
Standard deviation	24.19	20.94			
**Saturated fat (grams)**					
Average per-serving					
Standard deviation					
**Sugar (grams)**					
Average per-serving	28.12	29.35	1.24	4.30%	0.0000
Standard deviation	6.52	5.51			
**Sodium (milligrams)**					
Average per-serving	29.62	21.09	-8.53	-33.62%	0.0000
Standard deviation	16.81	14.75			
**Dietary fiber (grams)**					
Average per-serving					
Standard deviation					
**No. of UPCs**	1,844	667			

*Serving = 240 grams (8 oz)

Types and Frequencies of Reformulation: Tortilla & Other Chips

Calculations using InfoScan Data for UPCs with positive sales

Average per-serving nutrient values*	National	Private Label	2014 Difference	% Difference	p-value
All UPCs					
**Calories**					
Average per-serving	141.49	141.11	-0.38	-0.27%	0.7032
Standard deviation	16.44	15.37			
**Saturated fat (grams)**					
Average per-serving	1.52	1.13	-0.38	-28.84%	0.0000
Standard deviation	1.27	0.96			
**Sugar (grams)**					
Average per-serving	0.76	0.70	-0.07	-9.26%	0.3447
Standard deviation	1.10	1.40			
**Sodium (milligrams)**					
Average per-serving	220.86	171.92	-48.94	-24.92%	0.0000
Standard deviation	186.26	134.44			
**Dietary fiber (grams)**					
Average per-serving	1.34	1.33	-0.01	-0.99%	0.8110
Standard deviation	0.91	0.83			
**No. of UPCs**	1,593	315			

*Serving = 28 grams

Types and Frequencies of Reformulation: Yeast Breads

Calculations using InfoScan Data for UPCs with positive sales

Average per-serving nutrient values*	National	Private Label	2014 Difference	% Difference	p-value
All UPCs					
**Calories**					
Average per-serving	144.12	146.17	2.05	1.41%	0.0085
Standard deviation	22.09	18.56			
**Saturated fat (grams)**					
Average per-serving	0.15	0.13	-0.02	-13.60%	0.4069
Standard deviation	0.56	0.70			
**Sugar (grams)**					
Average per-serving	3.15	3.00	-0.15	-4.88%	0.1436
Standard deviation	2.75	2.75			
**Sodium (milligrams)**					
Average per-serving	270.90	290.83	19.93	7.10%	0.0000
Standard deviation	73.92	58.78			
**Dietary fiber (grams)**					
Average per-serving	2.64	2.01	-0.62	-26.89%	0.0000
Standard deviation	2.04	1.62			
**No. of UPCs**	1,936	1,143			

*Serving = 57 grams

Types and Frequencies of Reformulation: Yogurt

Calculations using InfoScan Data for UPCs with positive sales

Average per-serving nutrient values*	National	Private Label	2014 Difference	% Difference	p-value
All UPCs					
**Calories**					
Average per-serving	146.70	127.18	-19.52	-14.26%	0.0000
Standard deviation	43.19	38.27			
**Saturated fat (grams)**					
Average per-serving	1.39	0.44	-0.95	-103.11%	0.0000
Standard deviation	2.11	0.66			
**Sugar (grams)**					
Average per-serving	19.05	17.82	-1.24	-6.70%	0.0010
Standard deviation	7.55	8.69			
**Sodium (milligrams)**					
Average per-serving	89.25	96.10	6.85	7.39%	0.0000
Standard deviation	25.98	24.58			
**Dietary fiber (grams)**					
Average per-serving	0.43	0.41	-0.02	-3.66%	0.7982
Standard deviation	1.25	1.34			
**No. of UPCs**	1,174	749			

*Serving = 170 grams
