# Supplementary material for: How Much Can Product Reformulation Improve Diet Quality in Households with Children and Adolescents?
Source: Nutrients. 2019 Mar 14;11(3):618. doi: 10.3390/nu11030618 (PMC6470779; doi:10.3390/nu11030618)
Supplement: Supplementary file 1 [file nutrients-11-00618-s001.zip › Table S3 Detailed Version of Tables 3 and 4.pdf]

Comparison of Average Nutrient Values for Products in 2014 versus 1-star, 2-star, and 3-star Products

			1-star target			2-star target (if available)			3-star target (if available)		
Nutrient	Baseline (2014 Average)	Baseline (2014 St. Dev.)	Healthier Product Values	Change for Average Product	% Change Relative to Baseline	Healthier Product Values	Change for Average Product	% Change Relative to Baseline	Healthier Product Values	Change for Average Product	% Change Relative to Baseline
**Cereal bars**											
Calories	142.9	18.6	138.4	-4.5	-3.1%	143.3	0.4	0.3%			
Saturated Fat (g)	1.9	1.3	1.3	-0.6	-31.6%	1.1	-0.8	-42.1%			
Sugars (g)	9.4	3.9	8.1	-1.3	-13.8%	5.9	-3.5	-37.2%			
Sodium (mg)	104.2	47.2	80.5	-23.7	-22.7%	80.3	-23.9	-22.9%			
Dietary Fiber (g)	2.6	2.0	3.8	1.2	46.2%	4.2	1.6	61.5%			
**Cookies & brownies**											
Calories	136.3	18.4	127.8	-8.5	-6.2%	121.6	-14.7	-10.8%			
Saturated Fat (g)	2.4	1.5	0.7	-1.7	-70.8%	0.9	-1.5	-62.5%			
Sugars (g)	9.9	3.2	6.2	-3.7	-37.4%	4.4	-5.5	-55.6%			
Sodium (mg)	91.8	38.3	80.9	-10.9	-11.9%	92.7	0.9	1.0%			
Dietary Fiber (g)	0.7	0.8	2.9	2.2	314.3%	2.5	1.8	257.1%			
**Crackers**											
Calories	131.4	18.1	129.2	-2.2	-1.7%	131.6	0.2	0.2%	137.2	5.8	4.4%
Saturated Fat (g)	0.9	1.1	0.4	-0.5	-55.6%	0.4	-0.5	-55.6%	0.6	-0.3	-33.3%
Sugars (g)	2.2	2.6	0.5	-1.7	-77.3%	0.3	-1.9	-86.4%	0.2	-2.0	-90.9%
Sodium (mg)	209.2	101	175.1	-34.1	-16.3%	137	-72.2	-34.5%	122.0	-87.2	-41.7%
Dietary Fiber (g)	1.3	1.1	1.7	0.4	30.8%	3.2	1.9	146.2%	4.3	3.0	230.8%
**Fruit Drinks**											
Calories	94.9	41.1	25.8	-69.1	-72.8%						
Saturated Fat (g)											
Sugars (g)	22.6	10.1	5.7	-16.9	-74.8%						
Sodium (mg)	34.3	35.7	16.6	-17.7	-51.6%						
Dietary Fiber (g)											
**Ice Cream**											
Calories	140.2	38.9	85.9	-54.3	-38.7%						
Saturated Fat (g)	3.9	2.3	1.0	-2.9	-74.4%						
Sugars (g)	13.8	3.2	5.0	-8.8	-63.8%						
Sodium (mg)	57.9	31.6	66.7	8.8	15.2%						
Dietary Fiber (g)	0.5	0.9	3.7	3.2	640.0%						
**Macaroni & cheese mix**											
Calories	245.3	24.7	248.7	3.4	1.4%						
Saturated Fat (g)	1.8	1.2	1.8	0.0	0.0%						
Sugars (g)	4.7	2.2	4.6	-0.1	-2.1%						
Sodium (mg)	589.4	95.9	544.1	-45.3	-7.7%						
Dietary Fiber (g)	1.6	0.9	2.6	1.0	62.5%						

Comparison of Average Nutrient Values for Products in 2014 versus 1-star, 2-star, and 3-star Products

			1-star target			2-star target (if available)			3-star target (if available)		
Nutrient	Baseline (2014 Average)	Baseline (2014 St. Dev.)	Healthier Product Values	Change for Average Product	% Change Relative to Baseline	Healthier Product Values	Change for Average Product	% Change Relative to Baseline	Healthier Product Values	Change for Average Product	% Change Relative to Baseline
**Pizza**											
Calories	337.0	40.2	334.6	-2.4	-0.7%						
Saturated Fat (g)	5.9	2.1	4.7	-1.2	-20.3%						
Sugars (g)	4.2	2.6	2.9	-1.3	-31.0%						
Sodium (mg)	733.6	170.0	585.4	-148.2	-20.2%						
Dietary Fiber (g)	2.3	1.1	5.3	3.0	130.4%						
**Potato Chips**											
Calories	149.9	12.0	147.5	-2.4	-1.6%	140.4	-9.5	-6.3%			
Saturated Fat (g)	1.8	0.8	1.2	-0.6	-33.3%	0.9	-0.9	-50.0%			
Sugars (g)	0.9	0.9	0.5	-0.4	-44.4%	0.3	-0.6	-66.7%			
Sodium (mg)	193.0	84.1	135.1	-57.9	-30.0%	136.4	-56.6	-29.3%			
Dietary Fiber (g)	1.0	0.3	1.2	0.2	20.0%	2.1	1.1	110.0%			
**RTE cereal**											
Calories	115.1	11.8	114.6	-0.5	-0.4%	115.5	0.4	0.3%	106.5	-8.6	-7.5%
Saturated Fat (g)	0.2	0.4	0.2	0.0	0.0%	0.2	0.0	0.0%	0.1	-0.1	-50.0%
Sugars (g)	8.0	4.0	6.8	-1.2	-15.0%	5.4	-2.6	-32.5%	1.1	-6.9	-86.3%
Sodium (mg)	133.3	74.1	119.1	-14.2	-10.7%	74.5	-58.8	-44.1%	54.4	-78.9	-59.2%
Dietary Fiber (g)	2.0	1.6	2.6	0.6	30.0%	3.5	1.5	75.0%	2.6	0.6	30.0%
**Tortilla Chips and Other Chips**											
Calories	141.4	16.3	136.7	-4.7	-3.3%	130.7	-10.7	-7.6%	76.1	-65.3	-46.2%
Saturated Fat (g)	1.5	1.2	0.9	-0.6	-40.0%	0.6	-0.9	-60.0%	0.5	-1.0	-66.7%
Sugars (g)	0.8	1.2	1.2	0.4	50.0%	1.2	0.4	50.0%	2.1	1.3	162.5%
Sodium (mg)	212.8	179.6	133.1	-79.7	-37.5%	128.9	-83.9	-39.4%	79.3	-133.5	-62.7%
Dietary Fiber (g)	1.3	0.9	2.4	1.1	84.6%	3	1.7	130.8%	3.8	2.5	192.3%
**Yeast Breads**											
Calories	144.9	20.9	163	18.1	12.5%	123.1	-21.8	-15.0%	128.8	-16.1	-11.1%
Saturated Fat (g)	0.1	0.6	0.1	0.0	0.0%	0.1	0.0	0.0%	0.1	0.0	0.0%
Sugars (g)	3.1	2.8	2.8	-0.3	-9.7%	2.1	-1.0	-32.3%	1.9	-1.2	-38.7%
Sodium (mg)	278.3	69.4	274.5	-3.8	-1.4%	223.8	-54.5	-19.6%	201.9	-76.4	-27.5%
Dietary Fiber (g)	2.4	1.9	2.4	0.0	0.0%	4.6	2.2	91.7%	5.7	3.3	137.5%
**Yogurt**											
Calories	139.1	42.4	125.1	-14.0	-10.1%	94.4	-44.7	-32.1%	112.9	-26.2	-18.8%
Saturated Fat (g)	1.0	1.8	1.4	0.4	40.0%	0.6	-0.4	-40.0%	0	-1.0	-100.0%
Sugars (g)	18.6	8.0	12.7	-5.9	-31.7%	8.3	-10.3	-55.4%	7.9	-10.7	-57.5%
Sodium (mg)	91.9	25.7	85.1	-6.8	-7.4%	91.1	-0.8	-0.9%	86.9	-5.0	-5.4%
Dietary Fiber (g)	0.4	1.3	2.2	1.8	450.0%	0.8	0.4	100.0%	4.5	4.1	1025.0%

Note:

Sugars is total sugars, including added sugars.

Total Values for Annual Dietary Guidelines Targets by Demographic Group for Consumer Network Households with Children (Weighted), 2014
(units for nutrients = billions)

	All Households	Low Income (<185% of Poverty Line)	High Income (185% of Poverty Line)	Non-Hispanic White	Non-Hispanic Black	Hispanic	Asian and Other	Urban	Part Urban /Part Rural	Rural	Unspecified
**Annual Dietary Guidelines**											
Calories	95,320	59,298	36,023	61,302	9,758	17,305	6,955	60,205	24,302	5,398	5415
Saturated Fat (g)	1,059	659	400	681	108	192	77	669	270	60	60
Sugars (g)	2,383	1,483	900	1,533	244	433	174	1,505	608	135	135
Sodium (mg)	115,697	71,635	44,062	74,404	11,897	20,935	8,461	73,043	29,535	6,545	6574
Dietary Fiber (g)	1,334	830	504	858	137	242	97	843	340	75	76
**NHANES Nutrient Intakes**											
Calories	108,361	41,070	67,291	69,743	10,985	19,734	7,899	68,406	27,710	6,103	6,142
Saturated Fat (g)	1,375	522	853	885	140	250	100	868	352	77	78
Sugars (g)	6,084	2,320	3,763	3,919	614	1,109	442	3,836	1,559	343	346
Sodium (mg)	176,270	66,710	109,561	113,395	17,917	32,103	12,855	111,312	45,037	9,933	9,988
Dietary Fiber (g)	828	313	515	533	84	150	61	523	212	47	47
No. of households	36,059,594	13,308,488	22,751,106	23,265,029	3,787,819	6,338,461	2,668,285	22,903,936	9,131,670	1,983,848	2,040,140
No. of household members	145,420,117	55,617,187	89,802,930	93,696,943	14,783,685	26,325,781	10,613,708	91,691,941	37,260,768	8,205,123	8,262,285
No. of adult equivalent members	130,575,618	49,345,831	81,229,787	83,974,862	13,367,645	23,704,935	9,528,176	82,472,930	33,290,416	7,394,920	7,417,352
Avg. no. of members per HH	4.0	4.2	3.9	4.0	3.9	4.2	4.0	4.0	4.1	4.1	4.0
Avg. no. of adult equivalents per HH	3.6	3.7	3.6	3.6	3.5	3.7	3.6	3.6	3.6	3.7	3.6

Poverty line information: https://aspe.hhs.gov/2014-poverty-guidelines#tresholds; assigned households based on value of the low end of income range
Dietary Guidelines targets: https://health.gov/dietaryguidelines/2015/resources/2015-2020_Dietary_Guidelines.pdf

Annual targets calculated for 365 days of the year based on household composition in 2014.

No. of Servings Purchased by Demographic Group for Consumer Network Households with Children (Weighted), 2014

	Number of Households (unweighted)	All Households	Low Income (<185% of Poverty Line)	High Income (> 185% of Poverty Line)	Non-Hispanic White	Non-Hispanic Black	Hispanic	Asian and Other	Urban	Part Urban /Part Rural	Rural	Unspecified
Cereal Bars	12,033	3,900,324,546	1,094,145,575	2,806,178,970	2,858,861,477	250,014,011	556,544,634	234,904,424	2,479,040,492	990,442,622	205,095,190	225,746,241
Cookies and Brownies	13,527	9,214,308,144	3,519,230,675	5,695,077,469	6,264,746,689	925,099,566	1,485,350,202	539,111,687	5,715,315,420	2,398,751,124	583,245,575	516,996,025
Crackers	13,543	7,678,637,914	2,533,934,561	5,144,703,354	5,601,517,625	581,218,301	1,059,050,082	436,851,906	4,811,602,462	2,023,114,328	443,526,413	400,394,711
Fruit Drinks	12,461	4,777,911,066	2,004,096,826	2,773,814,240	2,593,355,892	916,523,232	993,032,961	274,998,981	3,202,210,937	1,093,740,577	228,409,556	253,549,996
Ice Cream	13,137	9,072,321,584	3,482,500,346	5,589,821,238	6,483,084,283	735,257,439	1,286,589,384	567,390,478	5,623,743,226	2,397,040,460	542,215,425	509,322,473
Macaroni & Cheese Mix	10,775	1,395,934,942	581,935,487	813,999,455	1,041,127,970	91,705,407	188,809,906	74,291,659	844,273,743	386,579,822	83,729,458	81,351,919
Frozen Pizza	11,009	1,599,660,363	667,529,057	932,131,306	1,170,538,921	131,831,366	219,415,310	77,874,765	907,394,864	478,108,611	114,876,695	99,280,193
Potato Chips	12,341	3,839,433,960	1,507,160,540	2,332,273,420	2,700,193,025	391,652,088	518,731,092	228,857,755	2,297,739,828	1,065,657,422	257,658,565	218,378,145
RTE Cereal	13,518	16,120,096,372	6,239,455,021	9,880,641,351	11,292,272,213	1,428,294,084	2,527,081,378	872,448,697	10,012,900,372	4,266,074,866	928,779,552	912,341,582
Tortilla Chips and Other Chips	13,427	6,465,872,608	2,316,545,535	4,149,327,073	4,579,693,812	509,220,311	1,024,280,007	352,678,478	3,877,617,170	1,817,127,378	395,807,049	375,321,011
Yeast Breads	13,669	9,278,137,478	3,707,640,784	5,570,496,694	6,352,847,137	856,090,922	1,478,847,907	590,351,512	5,737,669,856	2,422,862,471	584,576,636	533,028,515
Yogurt	12,619	2,715,118,283	811,924,345	1,903,193,938	1,954,198,924	178,803,309	406,920,271	175,195,780	1,751,040,505	697,946,634	135,359,596	130,771,548

Potential Improvements from Reformulation of All 12 "1-Star" Products by Demographic Group for Consumer Network Households with Children (Weighted), 2014

	All Households	Low Income (<185% of Poverty Line)	High Income (>185% of Poverty Line)	Non-Hispanic White	Non-Hispanic Black	Hispanic	Asian and Other	Urban	Part Urban /Part Rural	Rural	Unspecified
**Calories**											
Baseline (2014 average product)	10,496,414,932,914	3,936,944,951,955	6,559,469,980,947	7,347,778,684,405	939,201,976,028	1,600,365,052,262	609,069,220,022	6,490,897,175,344	2,785,999,161,798	629,057,133,321	590,461,462,308
Reformulated healthier product	9,644,033,468,524	3,607,443,512,090	6,036,589,956,425	6,792,814,883,071	834,618,607,089	1,458,287,842,272	558,312,135,883	5,945,217,967,629	2,571,983,517,730	582,727,176,967	544,104,806,059
Total change	-852,381,464,390	-329,501,439,865	-522,880,024,522	-554,963,801,334	-104,583,368,939	-142,077,209,990	-50,757,084,139	-545,679,207,715	-214,015,644,068	-46,329,956,354	-46,356,656,249
Baseline per AE per day	220.23	218.58	221.24	239.73	192.49	184.96	175.13	215.63	229.28	233.06	218.1
Reformulated per AE per day	202.35	200.29	203.6	221.62	171.06	168.54	160.54	197.5	211.67	215.89	200.97
Change per AE per day	-17.88	-18.29	-17.64	-18.11	-21.43	-16.42	-14.59	-18.13	-17.61	-17.16	-17.12
Percent change per AE per day	-8.12%	-8.37%	-7.97%	-7.55%	-11.13%	-8.88%	-8.33%	-8.41%	-7.68%	-7.36%	-7.85%
**Saturated Fat (g)**											
Baseline (2014 average product)	107,245,204,866	39,991,514,682	67,253,690,183	76,150,659,649	9,047,616,297	15,757,884,434	6,289,044,480	65,843,042,185	28,762,111,134	6,568,838,856	6,071,212,689
Reformulated healthier product	52,074,905,680	19,215,628,130	32,859,277,549	37,192,670,921	4,274,883,150	7,611,891,133	2,995,460,473	31,831,253,256	14,102,771,592	3,184,288,761	2,956,592,069
Total change	-55,170,299,186	-20,775,886,552	-34,394,412,634	-38,957,988,729	-4,772,733,147	-8,145,993,301	-3,293,584,007	-34,011,788,929	-14,659,339,542	-3,384,550,095	-3,114,620,620
Baseline per AE per day	2.25	2.22	2.27	2.48	1.85	1.82	1.81	2.19	2.37	2.43	2.24
Reformulated per AE per day	1.09	1.07	1.11	1.21	0.88	0.88	0.86	1.06	1.16	1.18	1.09
Change per AE per day	-1.16	-1.15	-1.16	-1.27	-0.98	-0.94	-0.95	-1.13	-1.21	-1.25	-1.15
Percent change per AE per day	-51.56%	-51.80%	-51.10%	-51.21%	-52.97%	-51.65%	-52.49%	-51.60%	-51.05%	-51.44%	-51.34%
**Sugars (g)**											
Baseline (2014 average product)	608,088,386,344	229,310,620,322	378,777,766,015	411,577,632,659	62,797,821,726	97,928,904,583	35,784,027,391	383,856,289,919	156,165,398,577	34,507,074,310	33,559,623,529
Reformulated healthier product	355,972,881,626	132,051,930,198	223,920,951,420	246,419,746,234	32,943,112,194	55,826,089,099	20,783,934,109	223,000,830,672	92,661,332,937	20,505,846,472	19,804,871,537
Total change	-252,115,504,717	-97,258,690,124	-154,856,814,594	-165,157,886,425	-29,854,709,532	-42,102,815,483	-15,000,093,282	-160,855,459,247	-63,504,065,640	-14,001,227,838	-13,754,751,992
Baseline per AE per day	12.76	12.73	12.78	13.43	12.87	11.32	10.29	12.75	12.85	12.78	12.40
Reformulated per AE per day	7.47	7.33	7.55	8.04	6.75	6.45	5.98	7.41	7.63	7.60	7.32
Change per AE per day	-5.29	-5.40	-5.22	-5.39	-6.12	-4.87	-4.31	-5.34	-5.23	-5.19	-5.08
Percent change per AE per day	-41.46%	-42.42%	-40.85%	-40.13%	-47.55%	-43.02%	-41.89%	-41.88%	-40.70%	-40.61%	-40.97%
**Sodium (mg)**											
Baseline (2014 average product)	12,641,485,345,295	4,792,258,344,319	7,849,227,001,081	8,930,049,686,503	1,086,361,942,485	1,900,603,741,825	724,469,973,841	7,749,354,077,654	3,400,987,849,821	773,440,377,083	717,703,040,633
Reformulated healthier product	10,861,473,658,955	4,131,340,660,492	6,730,132,998,558	7,674,386,546,652	929,077,323,290	1,631,843,692,245	626,166,096,268	6,666,337,892,703	2,914,688,965,233	664,214,210,755	616,232,590,184
Total change	-1,780,011,686,340	-660,917,683,828	-1,119,094,002,523	-1,255,663,139,851	-157,284,619,195	-268,760,049,580	-98,303,877,573	-1,083,016,184,951	-486,298,884,588	-109,226,166,328	-101,470,450,449
Baseline per AE per day	265.24	266.07	264.74	291.35	222.65	219.66	208.31	257.43	279.89	286.55	265.10
Reformulated per AE per day	227.89	229.38	226.99	250.38	190.42	188.60	180.05	221.45	239.87	246.08	227.62
Change per AE per day	-37.35	-36.69	-37.74	-40.97	-32.24	-31.06	-28.27	-35.98	-40.02	-40.47	-37.48
Percent change per AE per day	-14.08%	-13.79%	-14.26%	-14.06%	-14.48%	-14.14%	-13.57%	-13.98%	-14.30%	-14.12%	-14.14%
**Dietary Fiber (g)**											
Baseline (2014 average product)	104,860,802,698	39,030,706,077	65,830,096,619	73,966,773,952	8,907,125,625	15,930,032,993	6,056,870,125	64,786,293,812	27,855,144,310	6,274,289,575	5,945,074,998
Reformulated healthier product	190,550,087,728	70,882,854,872	119,667,232,854	134,589,695,118	16,132,167,280	28,732,578,437	11,095,646,890	117,706,453,954	50,649,529,249	11,432,238,007	10,761,866,515
Total change	85,689,285,031	31,852,148,795	53,837,136,235	60,622,921,166	7,225,041,655	12,802,545,444	5,038,776,765	52,920,160,142	22,794,384,938	5,157,948,432	4,816,791,517
Baseline per AE per day	2.20	2.17	2.22	2.41	1.83	1.84	1.74	2.15	2.29	2.32	2.20
Reformulated per AE per day	4.00	3.94	4.04	4.39	3.31	3.32	3.19	3.91	4.17	4.24	3.98
Change per AE per day	1.80	1.77	1.82	1.98	1.48	1.48	1.45	1.76	1.88	1.91	1.78
Percent change per AE per day	81.82%	81.57%	81.98%	82.16%	80.87%	80.43%	83.33%	81.86%	82.10%	82.33%	80.91%

Potential Improvements from Reformulation of All 12 "Max-Star" Products by Demographic Group for Consumer Network Households with Children (Weighted), 2014

	All Households	Low Income (<185% of Poverty Line)	High Income (>185% of Poverty Line)	Non-Hispanic White	Non-Hispanic Black	Hispanic	Asian and Other	Urban	Part Urban /Part Rural	Rural	Unspecified
**Calories**											
Baseline (2014 average product)	10,496,414,932,914	3,936,944,951,955	6,559,469,980,947	7,347,778,684,405	939,201,976,028	1,600,365,052,262	609,069,220,022	6,490,897,175,344	2,785,999,161,798	629,057,133,321	590,461,462,308
Reformulated healthier product	6,548,911,159,308	2,476,592,344,170	4,072,318,815,275	4,632,942,048,722	554,782,364,030	985,312,120,385	375,874,625,949	4,037,598,551,175	1,751,296,339,112	392,193,861,590	367,822,407,431
Total change	-3,947,503,773,606	-1,460,352,607,785	-2,487,151,165,672	-2,714,836,635,683	-384,419,611,997	-615,052,931,877	-233,194,594,072	-2,453,298,624,169	-1,034,702,822,686	-236,863,271,731	-222,639,054,877
Baseline per AE per day	220.23	218.58	221.24	239.73	192.49	184.96	175.13	215.63	229.28	233.06	218.10
Reformulated per AE per day	137.41	137.50	137.35	151.15	113.70	113.88	108.08	134.13	144.13	145.30	135.86
Change per AE per day	-82.83	-81.08	-83.89	-88.57	-78.79	-71.09	-67.05	-81.50	-85.15	-87.75	-82.24
Percent change per AE per day	-37.61%	-37.09%	-37.92%	-36.95%	-40.93%	-38.44%	-38.29%	-37.80%	-37.14%	-37.65%	-37.71%
**Saturated Fat (g)**											
Baseline (2014 average product)	107,245,204,866	39,991,514,682	67,253,690,183	76,150,659,649	9,047,616,297	15,757,884,434	6,289,044,480	65,843,042,185	28,762,111,134	6,568,838,856	6,071,212,689
Reformulated healthier product	29,483,350,623	11,340,713,875	18,142,636,749	21,273,916,974	2,351,714,229	4,205,862,153	1,651,857,263	17,809,018,909	8,131,320,631	1,848,203,907	1,694,807,176
Total change	-77,761,854,243	-28,650,800,807	-49,111,053,434	-54,876,742,676	-6,695,902,069	-11,552,022,281	-4,637,187,217	-48,034,023,276	-20,630,790,503	-4,720,634,949	-4,376,405,513
Baseline per AE per day	2.25	2.22	2.27	2.48	1.85	1.82	1.81	2.19	2.37	2.43	2.24
Reformulated per AE per day	0.62	0.63	0.61	0.69	0.48	0.49	0.47	0.59	0.67	0.68	0.63
Change per AE per day	-1.63	-1.59	-1.66	-1.79	-1.37	-1.34	-1.33	-1.60	-1.70	-1.75	-1.62
Percent change per AE per day	-72.44%	-71.62%	-73.13%	-72.18%	-74.05%	-73.63%	-73.48%	-73.06%	-71.73%	-72.02%	-72.32%
**Sugars (g)**											
Baseline (2014 average product)	608,088,386,344	229,310,620,322	378,777,766,015	411,577,632,659	62,797,821,726	97,928,904,583	35,784,027,391	383,856,289,919	156,165,398,577	34,507,074,310	33,559,623,529
Reformulated healthier product	155,580,078,495	59,142,244,018	96,437,834,477	106,049,042,557	15,500,474,150	24,765,133,475	9,265,428,318	97,740,722,469	40,414,795,558	8,852,903,526	8,571,656,943
Total change	-452,508,307,849	-170,168,376,304	-282,339,931,537	-305,528,590,102	-47,297,347,577	-73,163,771,107	-26,518,599,072	-286,115,567,450	-115,750,603,019	-25,654,170,784	-24,987,966,586
Baseline per AE per day	12.76	12.73	12.78	13.43	12.87	11.32	10.29	12.75	12.85	12.78	12.40
Reformulated per AE per day	3.26	3.28	3.25	3.46	3.18	2.86	2.66	3.25	3.33	3.28	3.17
Change per AE per day	-9.49	-9.45	-9.52	-9.97	-9.69	-8.46	-7.63	-9.50	-9.53	-9.50	-9.23
Percent change per AE per day	-74.37%	-74.23%	-74.49%	-74.24%	-75.29%	-74.73%	-74.15%	-74.51%	-74.16%	-74.33%	-74.44%
**Sodium (mg)**											
Baseline (2014 average product)	12,641,485,345,295	4,792,258,344,319	7,849,227,001,081	8,930,049,686,503	1,086,361,942,485	1,900,603,741,825	724,469,973,841	7,749,354,077,654	3,400,987,849,821	773,440,377,083	717,703,040,633
Reformulated healthier product	6,816,077,053,351	2,624,350,719,214	4,191,726,334,259	4,840,496,843,698	568,698,716,695	1,015,320,556,890	391,560,935,569	4,168,631,957,673	1,841,081,671,481	418,575,483,529	387,787,940,668
Total change	-5,825,408,291,944	-2,167,907,625,105	-3,657,500,666,822	-4,089,552,842,804	-517,663,225,790	-885,283,184,936	-332,909,038,272	-3,580,722,119,981	-1,559,906,178,340	-354,864,893,554	-329,915,099,966
Baseline per AE per day	265.24	266.07	264.74	291.35	222.65	219.66	208.31	257.43	279.89	286.55	265.10
Reformulated per AE per day	143.01	145.71	141.38	157.92	116.56	117.35	112.59	138.48	151.52	155.08	143.24
Change per AE per day	-122.23	-120.36	-123.36	-133.42	-106.10	-102.32	-95.72	-118.95	-128.38	-131.47	-121.86
Percent change per AE per day	-46.08%	-45.24%	-46.60%	-45.79%	-47.65%	-46.58%	-45.95%	-46.21%	-45.87%	-45.88%	-45.97%
**Dietary Fiber (g)**											
Baseline (2014 average product)	104,860,802,698	39,030,706,077	65,830,096,619	73,966,773,952	8,907,125,625	15,930,032,993	6,056,870,125	64,786,293,812	27,855,144,310	6,274,289,575	5,945,074,998
Reformulated healthier product	210,279,346,040	78,644,774,270	131,634,571,774	148,752,594,716	17,489,766,463	31,691,352,871	12,345,631,989	129,854,931,699	56,055,479,114	12,600,002,351	11,768,932,876
Total change	105,418,543,342	39,614,068,193	65,804,475,155	74,785,820,764	8,582,640,837	15,761,319,878	6,288,761,864	65,068,637,887	28,200,334,803	6,325,712,777	5,823,857,879
Baseline per AE per day	2.20	2.17	2.22	2.41	1.83	1.84	1.74	2.15	2.29	2.32	2.20
Reformulated per AE per day	4.41	4.37	4.44	4.85	3.58	3.66	3.55	4.31	4.61	4.67	4.35
Change per AE per day	2.21	2.20	2.22	2.44	1.76	1.82	1.81	2.16	2.32	2.34	2.15
Percent change per AE per day	100.45%	101.38%	100.00%	101.24%	96.17%	98.91%	104.02%	100.47%	101.31%	100.86%	97.73%

Potential Improvements from Reformulation of Cereal/Granola Bars by Demographic Group for Consumer Network Households with Children (Weighted), 2014

	All Households	Low Income (<185% of Poverty Line)	High Income (>185% of Poverty Line)	Non-Hispanic White	Non-Hispanic Black	Hispanic	Asian and Other	Urban	Part Urban /Part Rural	Rural	Unspecified
**1-Star Targets**											
**Calories**											
Baseline (2014 average product)	557,356,377,623	156,353,402,668	401,002,974,813	408,531,305,063	35,727,002,172	79,530,228,199	33,567,842,190	354,254,886,307	141,534,250,684	29,308,102,651	32,259,137,839
Reformulated healthier product	539,804,917,166	151,429,747,580	388,375,169,448	395,666,428,417	34,601,939,122	77,025,777,346	32,510,772,282	343,099,204,093	137,077,258,885	28,385,174,296	31,243,279,754
Total change	-17,551,460,457	-4,923,655,088	-12,627,805,365	-12,864,876,647	-1,125,063,050	-2,504,450,853	-1,057,069,908	-11,155,682,214	-4,456,991,799	-922,928,355	-1,015,858,085
Baseline per AE per day	11.69	8.68	13.53	13.33	7.32	9.19	9.65	11.77	11.65	10.86	11.92
Reformulated per AE per day	11.33	8.41	13.10	12.91	7.09	8.90	9.35	11.40	11.28	10.52	11.54
Change per AE per day	-0.37	-0.27	-0.43	-0.42	-0.23	-0.29	-0.30	-0.37	-0.37	-0.34	-0.38
Percent change per AE per day	-3.17%	-3.11%	-3.18%	-3.15%	-3.14%	-3.16%	-3.11%	-3.14%	-3.18%	-3.13%	-3.19%
**Saturated Fat (g)**											
Baseline (2014 average product)	7,410,616,637	2,078,876,593	5,331,740,043	5,431,836,806	475,026,621	1,057,434,805	446,318,406	4,710,176,935	1,881,840,982	389,680,861	428,917,858
Reformulated healthier product	5,070,421,910	1,422,389,248	3,648,032,661	3,716,519,920	325,018,214	723,508,024	305,375,751	3,222,752,640	1,287,575,409	266,623,747	293,470,113
Total change	-2,340,194,728	-656,487,345	-1,683,707,382	-1,715,316,886	-150,008,407	-333,926,780	-140,942,654	-1,487,424,295	-594,265,573	-123,057,114	-135,447,745
Baseline per AE per day	0.16	0.12	0.18	0.18	0.10	0.12	0.13	0.16	0.15	0.14	0.16
Reformulated per AE per day	0.11	0.08	0.12	0.12	0.07	0.08	0.09	0.11	0.11	0.10	0.11
Change per AE per day	-0.05	-0.04	-0.06	-0.06	-0.03	-0.04	-0.04	-0.05	-0.05	-0.05	-0.05
Percent change per AE per day	-31.25%	-33.33%	-33.33%	-33.33%	-30.00%	-33.33%	-30.77%	-31.25%	-33.33%	-35.71%	-31.25%
**Sugars (g)**											
Baseline (2014 average product)	36,663,050,732	10,284,968,405	26,378,082,318	26,873,297,884	2,350,131,703	5,231,519,560	2,208,101,586	23,302,980,625	9,310,160,647	1,927,894,786	2,122,014,665
Reformulated healthier product	31,592,628,823	8,862,579,158	22,730,049,657	23,156,777,964	2,025,113,489	4,508,011,535	1,902,725,834	20,080,227,985	8,022,585,238	1,661,271,039	1,828,544,552
Total change	-5,070,421,910	-1,422,389,248	-3,648,032,661	-3,716,519,920	-325,018,214	-723,508,024	-305,375,751	-3,222,752,640	-1,287,575,409	-266,623,747	-293,470,113
Baseline per AE per day	0.77	0.57	0.89	0.88	0.48	0.60	0.63	0.77	0.77	0.71	0.78
Reformulated per AE per day	0.66	0.49	0.77	0.76	0.42	0.52	0.55	0.67	0.66	0.62	0.68
Change per AE per day	-0.11	-0.08	-0.12	-0.12	-0.07	-0.08	-0.09	-0.11	-0.11	-0.10	-0.11
Percent change per AE per day	-14.29%	-14.04%	-13.48%	-13.64%	-14.58%	-13.33%	-14.29%	-14.29%	-14.29%	-14.08%	-14.10%
**Sodium (mg)**											
Baseline (2014 average product)	406,413,817,693	114,009,968,915	292,403,848,674	297,893,365,903	26,051,459,946	57,991,950,863	24,477,040,981	258,316,019,266	103,204,121,212	21,370,918,798	23,522,758,312
Reformulated healthier product	313,976,125,953	88,078,718,788	225,897,407,085	230,138,348,899	20,126,127,886	44,801,843,037	18,909,806,132	199,562,759,606	79,730,631,071	16,510,162,795	18,172,572,401
Total change	-92,437,691,740	-25,931,250,128	-66,506,441,589	-67,755,017,005	-5,925,332,061	-13,190,107,826	-5,567,234,849	-58,753,259,660	-23,473,490,141	-4,860,756,003	-5,350,185,912
Baseline per AE per day	8.53	6.33	9.86	9.72	5.34	6.70	7.04	8.58	8.49	7.92	8.69
Reformulated per AE per day	6.59	4.89	7.62	7.51	4.12	5.18	5.44	6.63	6.56	6.12	6.71
Change per AE per day	-1.94	-1.44	-2.24	-2.21	-1.21	-1.52	-1.60	-1.95	-1.93	-1.80	-1.98
Percent change per AE per day	-22.74%	-22.75%	-22.72%	-22.74%	-22.66%	-22.69%	-22.73%	-22.73%	-22.73%	-22.73%	-22.78%
**Dietary Fiber (g)**											
Baseline (2014 average product)	10,140,843,820	2,844,778,495	7,296,065,322	7,433,039,840	650,036,429	1,447,016,048	610,751,502	6,445,505,279	2,575,150,817	533,247,494	586,940,227
Reformulated healthier product	14,821,233,275	4,157,753,185	10,663,480,086	10,863,673,613	950,053,242	2,114,869,609	892,636,811	9,420,353,870	3,763,681,964	779,361,722	857,835,716
Total change	4,680,389,455	1,312,974,690	3,367,414,764	3,430,633,772	300,016,813	667,853,561	281,885,309	2,974,848,590	1,188,531,146	246,114,228	270,895,489
Baseline per AE per day	0.21	0.16	0.25	0.24	0.13	0.17	0.18	0.21	0.21	0.20	0.22
Reformulated per AE per day	0.31	0.23	0.36	0.35	0.19	0.24	0.26	0.31	0.31	0.29	0.32
Change per AE per day	0.10	0.07	0.11	0.11	0.06	0.08	0.08	0.10	0.10	0.09	0.10
Percent change per AE per day	47.62%	43.75%	44.00%	45.83%	46.15%	47.06%	44.44%	47.62%	47.62%	45.00%	45.45%

Potential Improvements from Reformulation of Cereal/Granola Bars by Demographic Group for Consumer Network Households with Children (Weighted), 2014

	All Households	Low Income (<185% of Poverty Line)	High Income (>185% of Poverty Line)	Non-Hispanic White	Non-Hispanic Black	Hispanic	Asian and Other	Urban	Part Urban /Part Rural	Rural	Unspecified
**2-Star Targets**											
**Calories**											
Baseline (2014 average product)	557,356,377,623	156,353,402,668	401,002,974,813	408,531,305,063	35,727,002,172	79,530,228,199	33,567,842,190	354,254,886,307	141,534,250,684	29,308,102,651	32,259,137,839
Reformulated healthier product	558,916,507,442	156,791,060,898	402,125,446,401	409,674,849,654	35,827,007,776	79,752,846,052	33,661,803,959	355,246,502,504	141,930,427,733	29,390,140,727	32,349,436,335
Total change	1,560,129,818	437,658,230	1,122,471,588	1,143,544,591	100,005,604	222,617,854	93,961,770	991,616,197	396,177,049	82,038,076	90,298,496
Baseline per AE per day	11.69	8.68	13.53	13.33	7.32	9.19	9.65	11.77	11.65	10.86	11.92
Reformulated per AE per day	11.73	8.71	13.56	13.37	7.34	9.22	9.68	11.80	11.68	10.89	11.95
Change per AE per day	0.03	0.02	0.04	0.04	0.02	0.03	0.03	0.03	0.03	0.03	0.03
Percent change per AE per day	0.26%	0.23%	0.30%	0.30%	0.27%	0.33%	0.31%	0.25%	0.26%	0.28%	0.25%
**Saturated Fat (g)**											
Baseline (2014 average product)	7,410,616,637	2,078,876,593	5,331,740,043	5,431,836,806	475,026,621	1,057,434,805	446,318,406	4,710,176,935	1,881,840,982	389,680,861	428,917,858
Reformulated healthier product	4,290,357,001	1,203,560,133	3,086,796,867	3,144,747,625	275,015,412	612,199,097	258,394,866	2,726,944,541	1,089,486,884	225,604,709	248,320,865
Total change	-3,120,259,637	-875,316,460	-2,244,943,176	-2,287,089,182	-200,011,209	-445,235,707	-187,923,539	-1,983,232,394	-792,354,098	-164,076,152	-180,596,993
Baseline per AE per day	0.16	0.12	0.18	0.18	0.10	0.12	0.13	0.16	0.15	0.14	0.16
Reformulated per AE per day	0.09	0.07	0.10	0.10	0.06	0.07	0.07	0.09	0.09	0.08	0.09
Change per AE per day	-0.07	-0.05	-0.08	-0.07	-0.04	-0.05	-0.05	-0.07	-0.07	-0.06	-0.07
Percent change per AE per day	-43.75%	-41.67%	-44.44%	-38.89%	-40.00%	-41.67%	-38.46%	-43.75%	-46.67%	-42.86%	-43.75%
**Sugars (g)**											
Baseline (2014 average product)	36,663,050,732	10,284,968,405	26,378,082,318	26,873,297,884	2,350,131,703	5,231,519,560	2,208,101,586	23,302,980,625	9,310,160,647	1,927,894,786	2,122,014,665
Reformulated healthier product	23,011,914,821	6,455,458,893	16,556,455,923	16,867,282,714	1,475,082,665	3,283,613,341	1,385,936,102	14,626,338,903	5,843,611,470	1,210,061,621	1,331,902,822
Total change	-13,651,135,911	-3,829,509,513	-9,821,626,395	-10,006,015,170	-875,049,039	-1,947,906,219	-822,165,484	-8,676,641,722	-3,466,549,177	-717,833,165	-790,111,844
Baseline per AE per day	0.77	0.57	0.89	0.88	0.48	0.60	0.63	0.77	0.77	0.71	0.78
Reformulated per AE per day	0.48	0.36	0.56	0.55	0.30	0.38	0.40	0.49	0.48	0.45	0.49
Change per AE per day	-0.29	-0.21	-0.33	-0.33	-0.18	-0.23	-0.24	-0.29	-0.29	-0.27	-0.29
Percent change per AE per day	-37.66%	-36.84%	-37.08%	-37.50%	-37.50%	-38.33%	-38.10%	-37.66%	-37.66%	-38.03%	-37.18%
**Sodium (mg)**											
Baseline (2014 average product)	406,413,817,693	114,009,968,915	292,403,848,674	297,893,365,903	26,051,459,946	57,991,950,863	24,477,040,981	258,316,019,266	103,204,121,212	21,370,918,798	23,522,758,312
Reformulated healthier product	313,196,061,044	87,859,889,673	225,336,171,291	229,566,576,603	20,076,125,083	44,690,534,110	18,862,825,247	199,066,951,508	79,532,542,547	16,469,143,757	18,127,423,152
Total change	-93,217,756,649	-26,150,079,243	-67,067,677,383	-68,326,789,300	-5,975,334,863	-13,301,416,753	-5,614,215,734	-59,249,067,759	-23,671,578,666	-4,901,775,041	-5,395,335,160
Baseline per AE per day	8.53	6.33	9.86	9.72	5.34	6.70	7.04	8.58	8.49	7.92	8.69
Reformulated per AE per day	6.57	4.88	7.60	7.49	4.11	5.17	5.42	6.61	6.55	6.10	6.70
Change per AE per day	-1.96	-1.45	-2.26	-2.23	-1.22	-1.54	-1.61	-1.97	-1.95	-1.82	-1.99
Percent change per AE per day	-22.98%	-22.91%	-22.92%	-22.94%	-22.85%	-22.99%	-22.87%	-22.96%	-22.97%	-22.98%	-22.90%
**Dietary Fiber (g)**											
Baseline (2014 average product)	10,140,843,820	2,844,778,495	7,296,065,322	7,433,039,840	650,036,429	1,447,016,048	610,751,502	6,445,505,279	2,575,150,817	533,247,494	586,940,227
Reformulated healthier product	16,381,363,093	4,595,411,415	11,785,951,674	12,007,218,203	1,050,058,846	2,337,487,463	986,598,581	10,411,970,066	4,159,859,012	861,399,798	948,134,212
Total change	6,240,519,274	1,750,632,920	4,489,886,352	4,574,178,363	400,022,418	890,471,414	375,847,078	3,966,464,787	1,584,708,195	328,152,304	361,193,986
Baseline per AE per day	0.21	0.16	0.25	0.24	0.13	0.17	0.18	0.21	0.21	0.20	0.22
Reformulated per AE per day	0.34	0.26	0.40	0.39	0.22	0.27	0.28	0.35	0.34	0.32	0.35
Change per AE per day	0.13	0.10	0.15	0.15	0.08	0.10	0.11	0.13	0.13	0.12	0.13
Percent change per AE per day	61.90%	62.50%	60.00%	62.50%	61.54%	58.82%	61.11%	61.90%	61.90%	60.00%	59.09%

Potential Improvements from Reformulation of Cookies & Brownies by Demographic Group for Consumer Network Households with Children (Weighted), 2014

	All Households	Low Income (<185% of Poverty Line)	High Income (>185% of Poverty Line)	Non-Hispanic White	Non-Hispanic Black	Hispanic	Asian and Other	Urban	Part Urban /Part Rural	Rural	Unspecified
**1-Star Targets**											
**Calories**											
Baseline (2014 average product)	1,255,910,200,027	479,671,141,003	776,239,059,025	853,884,973,711	126,091,070,846	202,453,232,533	73,480,922,938	778,997,491,746	326,949,778,201	79,496,371,873	70,466,558,208
Reformulated healthier product	1,177,588,580,803	449,757,680,265	727,830,900,538	800,634,626,854	118,227,724,535	189,827,755,816	68,898,473,599	730,417,310,676	306,560,393,647	74,538,784,485	66,072,091,995
Total change	-78,321,619,224	-29,913,460,738	-48,408,158,487	-53,250,346,857	-7,863,346,311	-12,625,476,717	-4,582,449,340	-48,580,181,070	-20,389,384,554	-4,957,587,388	-4,394,466,213
Baseline per AE per day	26.35	26.63	26.18	27.86	25.84	23.40	21.13	25.88	26.91	29.45	26.03
Reformulated per AE per day	24.71	24.97	24.55	26.12	24.23	21.94	19.81	24.26	25.23	27.62	24.40
Change per AE per day	-1.64	-1.66	-1.63	-1.74	-1.61	-1.46	-1.32	-1.61	-1.68	-1.84	-1.62
Percent change per AE per day	-6.22%	-6.23%	-6.23%	-6.25%	-6.23%	-6.24%	-6.25%	-6.22%	-6.24%	-6.25%	-6.22%
**Saturated Fat (g)**											
Baseline (2014 average product)	22,114,339,546	8,446,153,620	13,668,185,926	15,035,392,054	2,220,238,958	3,564,840,485	1,293,868,049	13,716,757,008	5,757,002,698	1,399,789,380	1,240,790,460
Reformulated healthier product	6,450,015,701	2,463,461,473	3,986,554,228	4,385,322,682	647,569,696	1,039,745,141	377,378,181	4,000,720,794	1,679,125,787	408,271,903	361,897,218
Total change	-15,664,323,845	-5,982,692,148	-9,681,631,697	-10,650,069,371	-1,572,669,262	-2,525,095,343	-916,489,868	-9,716,036,214	-4,077,876,911	-991,517,478	-878,893,243
Baseline per AE per day	0.46	0.47	0.46	0.49	0.46	0.41	0.37	0.46	0.47	0.52	0.46
Reformulated per AE per day	0.14	0.14	0.13	0.14	0.13	0.12	0.11	0.13	0.14	0.15	0.13
Change per AE per day	-0.33	-0.33	-0.33	-0.35	-0.32	-0.29	-0.26	-0.32	-0.34	-0.37	-0.32
Percent change per AE per day	-71.74%	-70.21%	-71.74%	-71.43%	-69.57%	-70.73%	-70.27%	-69.57%	-72.34%	-71.15%	-69.57%
**Sugars (g)**											
Baseline (2014 average product)	91,221,650,626	34,840,383,683	56,381,266,943	62,020,992,221	9,158,485,703	14,704,967,000	5,337,205,701	56,581,622,658	23,747,636,128	5,774,131,193	5,118,260,648
Reformulated healthier product	57,128,710,493	21,819,230,185	35,309,480,308	38,841,429,472	5,735,617,309	9,209,171,252	3,342,492,459	35,434,955,604	14,872,256,969	3,616,122,565	3,205,375,355
Total change	-34,092,940,133	-13,021,153,498	-21,071,786,635	-23,179,562,749	-3,422,868,394	-5,495,795,747	-1,994,713,242	-21,146,667,054	-8,875,379,159	-2,158,008,628	-1,912,885,293
Baseline per AE per day	1.91	1.93	1.90	2.02	1.88	1.70	1.53	1.88	1.95	2.14	1.89
Reformulated per AE per day	1.20	1.21	1.19	1.27	1.18	1.06	0.96	1.18	1.22	1.34	1.18
Change per AE per day	-0.72	-0.72	-0.71	-0.76	-0.70	-0.64	-0.57	-0.70	-0.73	-0.80	-0.71
Percent change per AE per day	-37.70%	-37.31%	-37.37%	-37.62%	-37.23%	-37.65%	-37.25%	-37.23%	-37.44%	-37.38%	-37.57%
**Sodium (mg)**											
Baseline (2014 average product)	845,873,487,619	323,065,375,965	522,808,111,654	575,103,746,050	84,924,140,159	136,355,148,544	49,490,452,867	524,665,955,556	220,205,353,183	53,541,943,785	47,460,235,095
Reformulated healthier product	745,437,528,850	284,705,761,608	460,731,767,242	506,818,007,140	74,840,554,889	120,164,831,342	43,614,135,478	462,369,017,478	194,058,965,932	47,184,567,018	41,824,978,423
Total change	-100,435,958,770	-38,359,614,358	-62,076,344,412	-68,285,738,910	-10,083,585,269	-16,190,317,202	-5,876,317,388	-62,296,938,078	-26,146,387,252	-6,357,376,768	-5,635,256,673
Baseline per AE per day	17.75	17.94	17.63	18.76	17.41	15.76	14.23	17.43	18.12	19.84	17.53
Reformulated per AE per day	15.64	15.81	15.54	16.54	15.34	13.89	12.54	15.36	15.97	17.48	15.45
Change per AE per day	-2.11	-2.13	-2.09	-2.23	-2.07	-1.87	-1.69	-2.07	-2.15	-2.36	-2.08
Percent change per AE per day	-11.89%	-11.87%	-11.85%	-11.89%	-11.89%	-11.87%	-11.88%	-11.88%	-11.87%	-11.90%	-11.87%
**Dietary Fiber (g)**											
Baseline (2014 average product)	6,450,015,701	2,463,461,473	3,986,554,228	4,385,322,682	647,569,696	1,039,745,141	377,378,181	4,000,720,794	1,679,125,787	408,271,903	361,897,218
Reformulated healthier product	26,721,493,618	10,205,768,958	16,515,724,660	18,167,765,398	2,682,788,741	4,307,515,586	1,563,423,892	16,574,414,718	6,956,378,260	1,691,412,168	1,499,288,473
Total change	20,271,477,917	7,742,307,485	12,529,170,432	13,782,442,716	2,035,219,045	3,267,770,444	1,186,045,711	12,573,693,924	5,277,252,473	1,283,140,265	1,137,391,255
Baseline per AE per day	0.14	0.14	0.13	0.14	0.13	0.12	0.11	0.13	0.14	0.15	0.13
Reformulated per AE per day	0.56	0.57	0.56	0.59	0.55	0.50	0.45	0.55	0.57	0.63	0.55
Change per AE per day	0.43	0.43	0.42	0.45	0.42	0.38	0.34	0.42	0.43	0.48	0.42
Percent change per AE per day	307.14%	307.14%	323.08%	321.43%	323.08%	316.67%	309.09%	323.08%	307.14%	320.00%	323.08%

Potential Improvements from Reformulation of Cookies & Brownies by Demographic Group for Consumer Network Households with Children (Weighted), 2014

	All Households	Low Income (<185% of Poverty Line)	High Income (>185% of Poverty Line)	Non-Hispanic White	Non-Hispanic Black	Hispanic	Asian and Other	Urban	Part Urban /Part Rural	Rural	Unspecified
**2-Star Targets**											
**Calories**											
Baseline (2014 average product)	1,255,910,200,027	479,671,141,003	776,239,059,025	853,884,973,711	126,091,070,846	202,453,232,533	73,480,922,938	778,997,491,746	326,949,778,201	79,496,371,873	70,466,558,208
Reformulated healthier product	1,120,459,870,310	427,938,450,080	692,521,420,230	761,793,197,382	112,492,107,226	180,618,584,563	65,555,981,139	694,982,355,072	291,688,136,678	70,922,661,920	62,866,716,640
Total change	-135,450,329,717	-51,732,690,923	-83,717,638,794	-92,091,776,328	-13,598,963,620	-21,834,647,969	-7,924,941,799	-84,015,136,674	-35,261,641,523	-8,573,709,953	-7,599,841,568
Baseline per AE per day	26.35	26.63	26.18	27.86	25.84	23.40	21.13	25.88	26.91	29.45	26.03
Reformulated per AE per day	23.51	23.76	23.36	24.85	23.06	20.88	18.85	23.09	24.01	26.28	23.22
Change per AE per day	-2.84	-2.87	-2.82	-3.00	-2.79	-2.52	-2.28	-2.79	-2.90	-3.18	-2.81
Percent change per AE per day	-10.78%	-10.78%	-10.77%	-10.77%	-10.80%	-10.77%	-10.79%	-10.78%	-10.78%	-10.80%	-10.80%
**Saturated Fat (g)**											
Baseline (2014 average product)	22,114,339,546	8,446,153,620	13,668,185,926	15,035,392,054	2,220,238,958	3,564,840,485	1,293,868,049	13,716,757,008	5,757,002,698	1,399,789,380	1,240,790,460
Reformulated healthier product	8,292,877,330	3,167,307,608	5,125,569,722	5,638,272,020	832,589,609	1,336,815,182	485,200,518	5,143,783,878	2,158,876,012	524,921,018	465,296,423
Total change	-13,821,462,216	-5,278,846,013	-8,542,616,204	-9,397,120,034	-1,387,649,349	-2,228,025,303	-808,667,531	-8,572,973,130	-3,598,126,686	-874,868,363	-775,494,038
Baseline per AE per day	0.46	0.47	0.46	0.49	0.46	0.41	0.37	0.46	0.47	0.52	0.46
Reformulated per AE per day	0.17	0.18	0.17	0.18	0.17	0.15	0.14	0.17	0.18	0.19	0.17
Change per AE per day	-0.29	-0.29	-0.29	-0.31	-0.28	-0.26	-0.23	-0.28	-0.30	-0.32	-0.29
Percent change per AE per day	-63.04%	-61.70%	-63.04%	-63.27%	-60.87%	-63.41%	-62.16%	-60.87%	-63.83%	-61.54%	-63.04%
**Sugars (g)**											
Baseline (2014 average product)	91,221,650,626	34,840,383,683	56,381,266,943	62,020,992,221	9,158,485,703	14,704,967,000	5,337,205,701	56,581,622,658	23,747,636,128	5,774,131,193	5,118,260,648
Reformulated healthier product	40,542,955,834	15,484,614,970	25,058,340,864	27,564,885,432	4,070,438,090	6,535,540,889	2,372,091,423	25,147,387,848	10,554,504,946	2,566,280,530	2,274,782,510
Total change	-50,678,694,792	-19,355,768,713	-31,322,926,080	-34,456,106,790	-5,088,047,613	-8,169,426,111	-2,965,114,279	-31,434,234,810	-13,193,131,182	-3,207,850,663	-2,843,478,138
Baseline per AE per day	1.91	1.93	1.90	2.02	1.88	1.70	1.53	1.88	1.95	2.14	1.89
Reformulated per AE per day	0.85	0.86	0.85	0.90	0.83	0.76	0.68	0.84	0.87	0.95	0.84
Change per AE per day	-1.06	-1.07	-1.06	-1.12	-1.04	-0.94	-0.85	-1.04	-1.09	-1.19	-1.05
Percent change per AE per day	-55.50%	-55.44%	-55.79%	-55.45%	-55.32%	-55.29%	-55.56%	-55.32%	-55.90%	-55.61%	-55.56%
**Sodium (mg)**											
Baseline (2014 average product)	845,873,487,619	323,065,375,965	522,808,111,654	575,103,746,050	84,924,140,159	136,355,148,544	49,490,452,867	524,665,955,556	220,205,353,183	53,541,943,785	47,460,235,095
Reformulated healthier product	854,166,364,949	326,232,683,573	527,933,681,376	580,742,018,070	85,756,729,768	137,691,963,725	49,975,653,385	529,809,739,434	222,364,229,195	54,066,864,803	47,925,531,518
Total change	8,292,877,330	3,167,307,608	5,125,569,722	5,638,272,020	832,589,609	1,336,815,182	485,200,518	5,143,783,878	2,158,876,012	524,921,018	465,296,423
Baseline per AE per day	17.75	17.94	17.63	18.76	17.41	15.76	14.23	17.43	18.12	19.84	17.53
Reformulated per AE per day	17.92	18.11	17.81	18.95	17.58	15.91	14.37	17.60	18.30	20.03	17.70
Change per AE per day	0.17	0.18	0.17	0.18	0.17	0.15	0.14	0.17	0.18	0.19	0.17
Percent change per AE per day	0.96%	1.00%	0.96%	0.96%	0.98%	0.95%	0.98%	0.98%	0.99%	0.96%	0.97%
**Dietary Fiber (g)**											
Baseline (2014 average product)	6,450,015,701	2,463,461,473	3,986,554,228	4,385,322,682	647,569,696	1,039,745,141	377,378,181	4,000,720,794	1,679,125,787	408,271,903	361,897,218
Reformulated healthier product	23,035,770,360	8,798,076,688	14,237,693,673	15,661,866,723	2,312,748,915	3,713,375,505	1,347,779,218	14,288,288,550	5,996,877,810	1,458,113,938	1,292,490,063
Total change	16,585,754,659	6,334,615,215	10,251,139,444	11,276,544,040	1,665,179,219	2,673,630,364	970,401,037	10,287,567,756	4,317,752,023	1,049,842,035	930,592,845
Baseline per AE per day	0.14	0.14	0.13	0.14	0.13	0.12	0.11	0.13	0.14	0.15	0.13
Reformulated per AE per day	0.48	0.49	0.48	0.51	0.47	0.43	0.39	0.47	0.49	0.54	0.48
Change per AE per day	0.35	0.35	0.35	0.37	0.34	0.31	0.28	0.34	0.36	0.39	0.34
Percent change per AE per day	250.00%	250.00%	269.23%	264.29%	261.54%	258.33%	254.55%	261.54%	257.14%	260.00%	261.54%

Potential Improvements from Reformulation of Crackers by Demographic Group for Consumer Network Households with Children (Weighted), 2014

	All Households	Low Income (<185% of Poverty Line)	High Income (>185% of Poverty Line)	Non-Hispanic White	Non-Hispanic Black	Hispanic	Asian and Other	Urban	Part Urban /Part Rural	Rural	Unspecified
**1-Star Targets**											
**Calories**											
Baseline (2014 average product)	1,008,973,021,900	332,959,001,315	676,014,020,716	736,039,415,925	76,372,084,751	139,159,180,775	57,402,340,448	632,244,563,507	265,837,222,699	58,279,370,668	52,611,865,025
Reformulated healthier product	992,080,018,489	327,384,345,281	664,695,673,337	723,716,077,150	75,093,404,489	136,829,270,594	56,441,266,255	621,659,038,090	261,386,371,178	57,303,612,560	51,730,996,661
Total change	-16,893,003,411	-5,574,656,034	-11,318,347,379	-12,323,338,775	-1,278,680,262	-2,329,910,180	-961,074,193	-10,585,525,416	-4,450,851,522	-975,758,109	-880,868,364
Baseline per AE per day	21.17	18.49	22.80	24.01	15.65	16.08	16.51	21.00	21.88	21.59	19.43
Reformulated per AE per day	20.82	18.18	22.42	23.61	15.39	15.81	16.23	20.65	21.51	21.23	19.11
Change per AE per day	-0.35	-0.31	-0.38	-0.40	-0.26	-0.27	-0.28	-0.35	-0.37	-0.36	-0.33
Percent change per AE per day	-1.65%	-1.68%	-1.67%	-1.67%	-1.66%	-1.68%	-1.70%	-1.67%	-1.69%	-1.67%	-1.70%
**Saturated Fat (g)**											
Baseline (2014 average product)	6,910,774,123	2,280,541,105	4,630,233,019	5,041,365,863	523,096,471	953,145,074	393,166,715	4,330,442,216	1,820,802,895	399,173,772	360,355,240
Reformulated healthier product	3,071,455,166	1,013,573,824	2,057,881,342	2,240,607,050	232,487,320	423,620,033	174,740,762	1,924,640,985	809,245,731	177,410,565	160,157,884
Total change	-3,839,318,957	-1,266,967,281	-2,572,351,677	-2,800,758,813	-290,609,151	-529,525,041	-218,425,953	-2,405,801,231	-1,011,557,164	-221,763,207	-200,197,356
Baseline per AE per day	0.15	0.13	0.16	0.16	0.11	0.11	0.11	0.14	0.15	0.15	0.13
Reformulated per AE per day	0.06	0.06	0.07	0.07	0.05	0.05	0.05	0.06	0.07	0.07	0.06
Change per AE per day	-0.08	-0.07	-0.09	-0.09	-0.06	-0.06	-0.06	-0.08	-0.08	-0.08	-0.07
Percent change per AE per day	-53.33%	-53.85%	-56.25%	-56.25%	-54.55%	-54.55%	-54.55%	-57.14%	-53.33%	-53.33%	-53.85%
**Sugars (g)**											
Baseline (2014 average product)	16,893,003,411	5,574,656,034	11,318,347,379	12,323,338,775	1,278,680,262	2,329,910,180	961,074,193	10,585,525,416	4,450,851,522	975,758,109	880,868,364
Reformulated healthier product	3,839,318,957	1,266,967,281	2,572,351,677	2,800,758,813	290,609,151	529,525,041	218,425,953	2,405,801,231	1,011,557,164	221,763,207	200,197,356
Total change	-13,053,684,454	-4,307,688,754	-8,745,995,702	-9,522,579,963	-988,071,112	-1,800,385,139	-742,648,240	-8,179,724,185	-3,439,294,358	-753,994,902	-680,671,009
Baseline per AE per day	0.35	0.31	0.38	0.40	0.26	0.27	0.28	0.35	0.37	0.36	0.33
Reformulated per AE per day	0.08	0.07	0.09	0.09	0.06	0.06	0.06	0.08	0.08	0.08	0.07
Change per AE per day	-0.27	-0.24	-0.29	-0.31	-0.20	-0.21	-0.21	-0.27	-0.28	-0.28	-0.25
Percent change per AE per day	-77.14%	-77.42%	-76.32%	-77.50%	-76.92%	-77.78%	-75.00%	-77.14%	-75.68%	-77.78%	-75.76%
**Sodium (mg)**											
Baseline (2014 average product)	1,606,371,051,609	530,099,110,161	1,076,271,941,657	1,171,837,487,150	121,590,868,569	221,553,277,154	91,389,418,735	1,006,587,235,050	423,235,517,418	92,785,725,600	83,762,573,541
Reformulated healthier product	1,344,529,498,741	443,691,941,631	900,837,557,285	980,825,736,138	101,771,324,505	185,439,669,358	76,492,768,741	842,511,591,096	354,247,318,833	77,661,474,916	70,109,113,896
Total change	-261,841,552,867	-86,407,168,530	-175,434,384,371	-191,011,751,013	-19,819,544,064	-36,113,607,796	-14,896,649,995	-164,075,643,954	-68,988,198,585	-15,124,250,683	-13,653,459,645
Baseline per AE per day	33.70	29.43	36.30	38.23	24.92	25.61	26.28	33.44	34.83	34.38	30.94
Reformulated per AE per day	28.21	24.63	30.38	32.00	20.86	21.43	21.99	27.99	29.15	28.77	25.90
Change per AE per day	-5.49	-4.80	-5.92	-6.23	-4.06	-4.17	-4.28	-5.45	-5.68	-5.60	-5.04
Percent change per AE per day	-16.29%	-16.31%	-16.31%	-16.30%	-16.29%	-16.28%	-16.29%	-16.30%	-16.31%	-16.29%	-16.29%
**Dietary Fiber (g)**											
Baseline (2014 average product)	9,982,229,288	3,294,114,929	6,688,114,360	7,281,972,913	755,583,791	1,376,765,107	567,907,478	6,255,083,201	2,630,048,626	576,584,337	520,513,124
Reformulated healthier product	13,053,684,454	4,307,688,754	8,745,995,702	9,522,579,963	988,071,112	1,800,385,139	742,648,240	8,179,724,185	3,439,294,358	753,994,902	680,671,009
Total change	3,071,455,166	1,013,573,824	2,057,881,342	2,240,607,050	232,487,320	423,620,033	174,740,762	1,924,640,985	809,245,731	177,410,565	160,157,884
Baseline per AE per day	0.21	0.18	0.23	0.24	0.15	0.16	0.16	0.21	0.22	0.21	0.19
Reformulated per AE per day	0.27	0.24	0.29	0.31	0.20	0.21	0.21	0.27	0.28	0.28	0.25
Change per AE per day	0.06	0.06	0.07	0.07	0.05	0.05	0.05	0.06	0.07	0.07	0.06
Percent change per AE per day	28.57%	33.33%	30.43%	29.17%	33.33%	31.25%	31.25%	28.57%	31.82%	33.33%	31.58%

Potential Improvements from Reformulation of Crackers by Demographic Group for Consumer Network Households with Children (Weighted), 2014

	All Households	Low Income (<185% of Poverty Line)	High Income (>185% of Poverty Line)	Non-Hispanic White	Non-Hispanic Black	Hispanic	Asian and Other	Urban	Part Urban /Part Rural	Rural	Unspecified
**2-Star Targets**											
**Calories**											
Baseline (2014 average product)	1,008,973,021,900	332,959,001,315	676,014,020,716	736,039,415,925	76,372,084,751	139,159,180,775	57,402,340,448	632,244,563,507	265,837,222,699	58,279,370,668	52,611,865,025
Reformulated healthier product	1,010,508,749,482	333,465,788,228	677,042,961,386	737,159,719,450	76,488,328,412	139,370,990,791	57,489,710,830	633,206,883,999	266,241,845,565	58,368,075,951	52,691,943,968
Total change	1,535,727,583	506,786,912	1,028,940,671	1,120,303,525	116,243,660	211,810,016	87,370,381	962,320,492	404,622,866	88,705,283	80,078,942
Baseline per AE per day	21.17	18.49	22.80	24.01	15.65	16.08	16.51	21.00	21.88	21.59	19.43
Reformulated per AE per day	21.20	18.51	22.84	24.05	15.68	16.11	16.53	21.03	21.91	21.62	19.46
Change per AE per day	0.03	0.03	0.03	0.04	0.02	0.02	0.03	0.03	0.03	0.03	0.03
Percent change per AE per day	0.14%	0.16%	0.13%	0.17%	0.13%	0.12%	0.18%	0.14%	0.14%	0.14%	0.15%
**Saturated Fat (g)**											
Baseline (2014 average product)	6,910,774,123	2,280,541,105	4,630,233,019	5,041,365,863	523,096,471	953,145,074	393,166,715	4,330,442,216	1,820,802,895	399,173,772	360,355,240
Reformulated healthier product	3,071,455,166	1,013,573,824	2,057,881,342	2,240,607,050	232,487,320	423,620,033	174,740,762	1,924,640,985	809,245,731	177,410,565	160,157,884
Total change	-3,839,318,957	-1,266,967,281	-2,572,351,677	-2,800,758,813	-290,609,151	-529,525,041	-218,425,953	-2,405,801,231	-1,011,557,164	-221,763,207	-200,197,356
Baseline per AE per day	0.15	0.13	0.16	0.16	0.11	0.11	0.11	0.14	0.15	0.15	0.13
Reformulated per AE per day	0.06	0.06	0.07	0.07	0.05	0.05	0.05	0.06	0.07	0.07	0.06
Change per AE per day	-0.08	-0.07	-0.09	-0.09	-0.06	-0.06	-0.06	-0.08	-0.08	-0.08	-0.07
Percent change per AE per day	-53.33%	-53.85%	-56.25%	-56.25%	-54.55%	-54.55%	-54.55%	-57.14%	-53.33%	-53.33%	-53.85%
**Sugars (g)**											
Baseline (2014 average product)	16,893,003,411	5,574,656,034	11,318,347,379	12,323,338,775	1,278,680,262	2,329,910,180	961,074,193	10,585,525,416	4,450,851,522	975,758,109	880,868,364
Reformulated healthier product	2,303,591,374	760,180,368	1,543,411,006	1,680,455,288	174,365,490	317,715,025	131,055,572	1,443,480,739	606,934,298	133,057,924	120,118,413
Total change	-14,589,412,037	-4,814,475,666	-9,774,936,373	-10,642,883,488	-1,104,314,772	-2,012,195,156	-830,018,621	-9,142,044,678	-3,843,917,223	-842,700,185	-760,749,951
Baseline per AE per day	0.35	0.31	0.38	0.40	0.26	0.27	0.28	0.35	0.37	0.36	0.33
Reformulated per AE per day	0.05	0.04	0.05	0.05	0.04	0.04	0.04	0.05	0.05	0.05	0.04
Change per AE per day	-0.31	-0.27	-0.33	-0.35	-0.23	-0.23	-0.24	-0.30	-0.32	-0.31	-0.28
Percent change per AE per day	-88.57%	-87.10%	-86.84%	-87.50%	-88.46%	-85.19%	-85.71%	-85.71%	-86.49%	-86.11%	-84.85%
**Sodium (mg)**											
Baseline (2014 average product)	1,606,371,051,609	530,099,110,161	1,076,271,941,657	1,171,837,487,150	121,590,868,569	221,553,277,154	91,389,418,735	1,006,587,235,050	423,235,517,418	92,785,725,600	83,762,573,541
Reformulated healthier product	1,051,973,394,218	347,149,034,857	704,824,359,498	767,407,914,625	79,626,907,237	145,089,861,234	59,848,711,122	659,189,537,294	277,166,662,936	60,763,118,581	54,854,075,407
Total change	-554,397,657,391	-182,950,075,304	-371,447,582,159	-404,429,572,525	-41,963,961,332	-76,463,415,920	-31,540,707,613	-347,397,697,756	-146,068,854,482	-32,022,607,019	-28,908,498,134
Baseline per AE per day	33.70	29.43	36.30	38.23	24.92	25.61	26.28	33.44	34.83	34.38	30.94
Reformulated per AE per day	22.07	19.27	23.77	25.04	16.32	16.77	17.21	21.90	22.81	22.51	20.26
Change per AE per day	-11.63	-10.16	-12.53	-13.19	-8.60	-8.84	-9.07	-11.54	-12.02	-11.86	-10.68
Percent change per AE per day	-34.51%	-34.52%	-34.52%	-34.50%	-34.51%	-34.52%	-34.51%	-34.51%	-34.51%	-34.50%	-34.52%
**Dietary Fiber (g)**											
Baseline (2014 average product)	9,982,229,288	3,294,114,929	6,688,114,360	7,281,972,913	755,583,791	1,376,765,107	567,907,478	6,255,083,201	2,630,048,626	576,584,337	520,513,124
Reformulated healthier product	24,571,641,325	8,108,590,595	16,463,050,733	17,924,856,400	1,859,898,563	3,388,960,262	1,397,926,099	15,397,127,878	6,473,965,850	1,419,284,522	1,281,263,075
Total change	14,589,412,037	4,814,475,666	9,774,936,373	10,642,883,488	1,104,314,772	2,012,195,156	830,018,621	9,142,044,678	3,843,917,223	842,700,185	760,749,951
Baseline per AE per day	0.21	0.18	0.23	0.24	0.15	0.16	0.16	0.21	0.22	0.21	0.19
Reformulated per AE per day	0.52	0.45	0.56	0.58	0.38	0.39	0.40	0.51	0.53	0.53	0.47
Change per AE per day	0.31	0.27	0.33	0.35	0.23	0.23	0.24	0.30	0.32	0.31	0.28
Percent change per AE per day	147.62%	150.00%	143.48%	145.83%	153.33%	143.75%	150.00%	142.86%	145.45%	147.62%	147.37%

Potential Improvements from Reformulation of Crackers by Demographic Group for Consumer Network Households with Children (Weighted), 2014

	All Households	Low Income (<185% of Poverty Line)	High Income (>185% of Poverty Line)	Non-Hispanic White	Non-Hispanic Black	Hispanic	Asian and Other	Urban	Part Urban /Part Rural	Rural	Unspecified
**3-Star Targets**											
**Calories**											
Baseline (2014 average product)	1,008,973,021,900	332,959,001,315	676,014,020,716	736,039,415,925	76,372,084,751	139,159,180,775	57,402,340,448	632,244,563,507	265,837,222,699	58,279,370,668	52,611,865,025
Reformulated healthier product	1,053,509,121,801	347,655,821,769	705,853,300,169	768,528,218,150	79,743,150,897	145,301,671,250	59,936,081,503	660,151,857,786	277,571,285,802	60,851,823,864	54,934,154,349
Total change	44,536,099,901	14,696,820,454	29,839,279,453	32,488,802,225	3,371,066,146	6,142,490,476	2,533,741,055	27,907,294,280	11,734,063,102	2,572,453,195	2,322,289,324
Baseline per AE per day	21.17	18.49	22.80	24.01	15.65	16.08	16.51	21.00	21.88	21.59	19.43
Reformulated per AE per day	22.10	19.30	23.81	25.07	16.34	16.79	17.23	21.93	22.84	22.54	20.29
Change per AE per day	0.93	0.82	1.01	1.06	0.69	0.71	0.73	0.93	0.97	0.95	0.86
Percent change per AE per day	4.39%	4.43%	4.43%	4.41%	4.41%	4.42%	4.42%	4.43%	4.43%	4.40%	4.43%
**Saturated Fat (g)**											
Baseline (2014 average product)	6,910,774,123	2,280,541,105	4,630,233,019	5,041,365,863	523,096,471	953,145,074	393,166,715	4,330,442,216	1,820,802,895	399,173,772	360,355,240
Reformulated healthier product	4,607,182,748	1,520,360,737	3,086,822,012	3,360,910,575	348,730,981	635,430,049	262,111,144	2,886,961,477	1,213,868,597	266,115,848	240,236,827
Total change	-2,303,591,374	-760,180,368	-1,543,411,006	-1,680,455,288	-174,365,490	-317,715,025	-131,055,572	-1,443,480,739	-606,934,298	-133,057,924	-120,118,413
Baseline per AE per day	0.15	0.13	0.16	0.16	0.11	0.11	0.11	0.14	0.15	0.15	0.13
Reformulated per AE per day	0.10	0.08	0.10	0.11	0.07	0.07	0.08	0.10	0.10	0.10	0.09
Change per AE per day	-0.05	-0.04	-0.05	-0.05	-0.04	-0.04	-0.04	-0.05	-0.05	-0.05	-0.04
Percent change per AE per day	-33.33%	-30.77%	-31.25%	-31.25%	-36.36%	-36.36%	-36.36%	-35.71%	-33.33%	-33.33%	-30.77%
**Sugars (g)**											
Baseline (2014 average product)	16,893,003,411	5,574,656,034	11,318,347,379	12,323,338,775	1,278,680,262	2,329,910,180	961,074,193	10,585,525,416	4,450,851,522	975,758,109	880,868,364
Reformulated healthier product	1,535,727,583	506,786,912	1,028,940,671	1,120,303,525	116,243,660	211,810,016	87,370,381	962,320,492	404,622,866	88,705,283	80,078,942
Total change	-15,357,275,828	-5,067,869,122	-10,289,406,708	-11,203,035,250	-1,162,436,602	-2,118,100,164	-873,703,812	-9,623,204,924	-4,046,228,656	-887,052,826	-800,789,422
Baseline per AE per day	0.35	0.31	0.38	0.40	0.26	0.27	0.28	0.35	0.37	0.36	0.33
Reformulated per AE per day	0.03	0.03	0.03	0.04	0.02	0.02	0.03	0.03	0.03	0.03	0.03
Change per AE per day	-0.32	-0.28	-0.35	-0.37	-0.24	-0.24	-0.25	-0.32	-0.33	-0.33	-0.30
Percent change per AE per day	-91.43%	-90.32%	-92.11%	-92.50%	-92.31%	-88.89%	-89.29%	-91.43%	-89.19%	-91.67%	-90.91%
**Sodium (mg)**											
Baseline (2014 average product)	1,606,371,051,609	530,099,110,161	1,076,271,941,657	1,171,837,487,150	121,590,868,569	221,553,277,154	91,389,418,735	1,006,587,235,050	423,235,517,418	92,785,725,600	83,762,573,541
Reformulated healthier product	936,793,825,508	309,140,016,442	627,653,809,188	683,385,150,250	70,908,632,722	129,204,110,004	53,295,932,532	587,015,500,364	246,819,948,016	54,110,222,386	48,848,154,742
Total change	-669,577,226,101	-220,959,093,719	-448,618,132,469	-488,452,336,900	-50,682,235,847	-92,349,167,150	-38,093,486,203	-419,571,734,686	-176,415,569,402	-38,675,503,214	-34,914,418,799
Baseline per AE per day	33.70	29.43	36.30	38.23	24.92	25.61	26.28	33.44	34.83	34.38	30.94
Reformulated per AE per day	19.66	17.16	21.17	22.30	14.53	14.93	15.32	19.50	20.31	20.05	18.04
Change per AE per day	-14.05	-12.27	-15.13	-15.94	-10.39	-10.67	-10.95	-13.94	-14.52	-14.33	-12.90
Percent change per AE per day	-41.69%	-41.69%	-41.68%	-41.70%	-41.69%	-41.66%	-41.67%	-41.69%	-41.69%	-41.68%	-41.69%
**Dietary Fiber (g)**											
Baseline (2014 average product)	9,982,229,288	3,294,114,929	6,688,114,360	7,281,972,913	755,583,791	1,376,765,107	567,907,478	6,255,083,201	2,630,048,626	576,584,337	520,513,124
Reformulated healthier product	33,018,143,030	10,895,918,612	22,122,224,422	24,086,525,788	2,499,238,694	4,553,915,353	1,878,463,196	20,689,890,587	8,699,391,610	1,907,163,576	1,721,697,257
Total change	23,035,913,742	7,601,803,683	15,434,110,062	16,804,552,875	1,743,654,903	3,177,150,246	1,310,555,718	14,434,807,386	6,069,342,984	1,330,579,239	1,201,184,133
Baseline per AE per day	0.21	0.18	0.23	0.24	0.15	0.16	0.16	0.21	0.22	0.21	0.19
Reformulated per AE per day	0.69	0.60	0.75	0.79	0.51	0.53	0.54	0.69	0.72	0.71	0.64
Change per AE per day	0.48	0.42	0.52	0.55	0.36	0.37	0.38	0.48	0.50	0.49	0.44
Percent change per AE per day	228.57%	233.33%	226.09%	229.17%	240.00%	231.25%	237.50%	228.57%	227.27%	233.33%	231.58%

Potential Improvements from Reformulation of Fruit Drinks by Demographic Group for Consumer Network Households with Children (Weighted), 2014

	All Households	Low Income (<185% of Poverty Line)	High Income (>185% of Poverty Line)	Non-Hispanic White	Non-Hispanic Black	Hispanic	Asian and Other	Urban	Part Urban /Part Rural	Rural	Unspecified
**1-Star Targets**											
**Calories**											
Baseline (2014 average product)	453,423,760,163	190,188,788,787	263,234,971,376	246,109,474,151	86,978,054,717	94,238,827,999	26,097,403,297	303,889,817,921	103,795,980,757	21,676,066,864	24,061,894,620
Reformulated healthier product	123,270,105,503	51,705,698,111	71,564,407,392	66,908,582,014	23,646,299,386	25,620,250,394	7,094,973,710	82,617,042,175	28,218,506,887	5,892,966,545	6,541,589,897
Total change	-330,153,654,661	-138,483,090,677	-191,670,563,984	-179,200,892,137	-63,331,755,331	-68,618,577,605	-19,002,429,587	-221,272,775,747	-75,577,473,871	-15,783,100,320	-17,520,304,724
Baseline per AE per day	9.51	10.56	8.88	8.03	17.83	10.89	7.50	10.10	8.54	8.03	8.89
Reformulated per AE per day	2.59	2.87	2.41	2.18	4.85	2.96	2.04	2.74	2.32	2.18	2.42
Change per AE per day	-6.93	-7.69	-6.46	-5.85	-12.98	-7.93	-5.46	-7.35	-6.22	-5.85	-6.47
Percent change per AE per day	-72.87%	-72.82%	-72.75%	-72.85%	-72.80%	-72.82%	-72.80%	-72.77%	-72.83%	-72.85%	-72.78%
**Saturated Fat (g)**											
Baseline (2014 average product)											
Reformulated healthier product											
Total change											
Baseline per AE per day											
Reformulated per AE per day											
Change per AE per day											
Percent change per AE per day											
**Sugars (g)**											
Baseline (2014 average product)	107,980,790,092	45,292,588,268	62,688,201,824	58,609,843,159	20,713,425,043	22,442,544,919	6,214,976,971	72,369,967,176	24,718,537,040	5,162,055,966	5,730,229,910
Reformulated healthier product	27,234,093,076	11,423,351,908	15,810,741,168	14,782,128,584	5,224,182,422	5,660,287,878	1,567,494,192	18,252,602,341	6,234,321,289	1,301,934,469	1,445,234,977
Total change	-80,746,697,015	-33,869,236,359	-46,877,460,656	-43,827,714,575	-15,489,242,621	-16,782,257,041	-4,647,482,779	-54,117,364,835	-18,484,215,751	-3,860,121,496	-4,284,994,932
Baseline per AE per day	2.27	2.51	2.11	1.91	4.25	2.59	1.79	2.40	2.03	1.91	2.12
Reformulated per AE per day	0.57	0.63	0.53	0.48	1.07	0.65	0.45	0.61	0.51	0.48	0.53
Change per AE per day	-1.69	-1.88	-1.58	-1.43	-3.17	-1.94	-1.34	-1.80	-1.52	-1.43	-1.58
Percent change per AE per day	-74.45%	-74.90%	-74.88%	-74.87%	-74.59%	-74.90%	-74.86%	-75.00%	-74.88%	-74.87%	-74.53%
**Sodium (mg)**											
Baseline (2014 average product)	163,882,349,564	68,740,521,132	95,141,828,432	88,952,107,096	31,436,746,858	34,061,030,562	9,432,465,048	109,835,835,139	37,515,301,791	7,834,447,771	8,696,764,863
Reformulated healthier product	79,313,323,696	33,268,007,312	46,045,316,384	43,049,707,807	15,214,285,651	16,484,347,153	4,564,983,085	53,156,701,554	18,156,093,578	3,791,598,630	4,208,929,934
Total change	-84,569,025,868	-35,472,513,820	-49,096,512,048	-45,902,399,288	-16,222,461,206	-17,576,683,410	-4,867,481,964	-56,679,133,585	-19,359,208,213	-4,042,849,141	-4,487,834,929
Baseline per AE per day	3.44	3.82	3.21	2.90	6.44	3.94	2.71	3.65	3.09	2.90	3.21
Reformulated per AE per day	1.66	1.85	1.55	1.40	3.12	1.91	1.31	1.77	1.49	1.40	1.55
Change per AE per day	-1.77	-1.97	-1.66	-1.50	-3.32	-2.03	-1.40	-1.88	-1.59	-1.50	-1.66
Percent change per AE per day	-51.45%	-51.57%	-51.71%	-51.72%	-51.55%	-51.52%	-51.66%	-51.51%	-51.46%	-51.72%	-51.71%
**Dietary Fiber (g)**											
Baseline (2014 average product)											
Reformulated healthier product											
Total change											
Baseline per AE per day											
Reformulated per AE per day											
Change per AE per day											
Percent change per AE per day											

Potential Improvements from Reformulation of Ice Cream by Demographic Group for Consumer Network Households with Children (Weighted), 2014

	All Households	Low Income (<185% of Poverty Line)	High Income (>185% of Poverty Line)	Non-Hispanic White	Non-Hispanic Black	Hispanic	Asian and Other	Urban	Part Urban /Part Rural	Rural	Unspecified
**1-Star Targets**											
**Calories**											
Baseline (2014 average product)	1,271,939,486,077	488,246,548,509	783,692,937,568	908,928,416,477	103,083,092,948	180,379,831,637	79,548,145,016	788,448,800,285	336,065,072,492	76,018,602,585	71,407,010,715
Reformulated healthier product	779,312,424,066	299,146,779,721	480,165,644,344	556,896,939,910	63,158,614,010	110,518,028,086	48,738,842,060	483,079,543,113	205,905,775,514	46,576,305,008	43,750,800,431
Total change	-492,627,062,011	-189,099,768,788	-303,527,293,223	-352,031,476,567	-39,924,478,938	-69,861,803,551	-30,809,302,955	-305,369,257,172	-130,159,296,978	-29,442,297,578	-27,656,210,284
Baseline per AE per day	26.69	27.11	26.43	29.65	21.13	20.85	22.87	26.19	27.66	28.16	26.38
Reformulated per AE per day	16.35	16.61	16.20	18.17	12.94	12.77	14.01	16.05	16.95	17.26	16.16
Change per AE per day	-10.34	-10.50	-10.24	-11.49	-8.18	-8.07	-8.86	-10.14	-10.71	-10.91	-10.22
Percent change per AE per day	-38.74%	-38.73%	-38.74%	-38.75%	-38.71%	-38.71%	-38.74%	-38.72%	-38.72%	-38.74%	-38.74%
**Saturated Fat (g)**											
Baseline (2014 average product)	35,382,054,178	13,581,751,349	21,800,302,828	25,284,028,704	2,867,504,012	5,017,698,598	2,212,822,864	21,932,598,581	9,348,457,794	2,114,640,158	1,986,357,645
Reformulated healthier product	9,072,321,584	3,482,500,346	5,589,821,238	6,483,084,283	735,257,439	1,286,589,384	567,390,478	5,623,743,226	2,397,040,460	542,215,425	509,322,473
Total change	-26,309,732,594	-10,099,251,003	-16,210,481,590	-18,800,944,421	-2,132,246,573	-3,731,109,214	-1,645,432,386	-16,308,855,355	-6,951,417,334	-1,572,424,733	-1,477,035,172
Baseline per AE per day	0.74	0.75	0.74	0.82	0.59	0.58	0.64	0.73	0.77	0.78	0.73
Reformulated per AE per day	0.19	0.19	0.19	0.21	0.15	0.15	0.16	0.19	0.20	0.20	0.19
Change per AE per day	-0.55	-0.56	-0.55	-0.61	-0.44	-0.43	-0.47	-0.54	-0.57	-0.58	-0.55
Percent change per AE per day	-74.32%	-74.67%	-74.32%	-74.39%	-74.58%	-74.14%	-73.44%	-73.97%	-74.03%	-74.36%	-75.34%
**Sugars (g)**											
Baseline (2014 average product)	125,198,037,859	48,058,504,775	77,139,533,084	89,466,563,105	10,146,552,658	17,754,933,499	7,829,988,596	77,607,656,519	33,079,158,348	7,482,572,865	7,028,650,127
Reformulated healthier product	45,361,607,920	17,412,501,730	27,949,106,190	32,415,421,415	3,676,287,195	6,432,946,920	2,836,952,390	28,118,716,130	11,985,202,300	2,711,077,125	2,546,612,365
Total change	-79,836,429,939	-30,646,003,045	-49,190,426,894	-57,051,141,690	-6,470,265,463	-11,321,986,579	-4,993,036,206	-49,488,940,389	-21,093,956,048	-4,771,495,740	-4,482,037,762
Baseline per AE per day	2.63	2.67	2.60	2.92	2.08	2.05	2.25	2.58	2.72	2.77	2.60
Reformulated per AE per day	0.95	0.97	0.94	1.06	0.75	0.74	0.82	0.93	0.99	1.00	0.94
Change per AE per day	-1.68	-1.70	-1.66	-1.86	-1.33	-1.31	-1.44	-1.64	-1.74	-1.77	-1.66
Percent change per AE per day	-63.88%	-63.67%	-63.85%	-63.70%	-63.94%	-63.90%	-64.00%	-63.57%	-63.97%	-63.90%	-63.85%
**Sodium (mg)**											
Baseline (2014 average product)	525,287,419,714	201,636,770,033	323,650,649,680	375,370,579,986	42,571,405,718	74,493,525,334	32,851,908,676	325,614,732,785	138,788,642,634	31,394,273,108	29,489,771,187
Reformulated healthier product	605,123,849,653	232,282,773,078	372,841,076,575	432,421,721,676	49,041,671,181	85,815,511,913	37,844,944,883	375,103,673,174	159,882,598,682	36,165,768,848	33,971,808,949
Total change	79,836,429,939	30,646,003,045	49,190,426,894	57,051,141,690	6,470,265,463	11,321,986,579	4,993,036,206	49,488,940,389	21,093,956,048	4,771,495,740	4,482,037,762
Baseline per AE per day	11.02	11.20	10.92	12.25	8.73	8.61	9.45	10.82	11.42	11.63	10.89
Reformulated per AE per day	12.70	12.90	12.58	14.11	10.05	9.92	10.88	12.46	13.16	13.40	12.55
Change per AE per day	1.68	1.70	1.66	1.86	1.33	1.31	1.44	1.64	1.74	1.77	1.66
Percent change per AE per day	15.25%	15.18%	15.20%	15.18%	15.23%	15.21%	15.24%	15.16%	15.24%	15.22%	15.24%
**Dietary Fiber (g)**											
Baseline (2014 average product)	4,536,160,792	1,741,250,173	2,794,910,619	3,241,542,142	367,628,720	643,294,692	283,695,239	2,811,871,613	1,198,520,230	271,107,713	254,661,237
Reformulated healthier product	33,567,589,861	12,885,251,280	20,682,338,581	23,987,411,847	2,720,452,524	4,760,380,721	2,099,344,769	20,807,849,936	8,869,049,702	2,006,197,073	1,884,493,150
Total change	29,031,429,069	11,144,001,107	17,887,427,962	20,745,869,706	2,352,823,805	4,117,086,029	1,815,649,530	17,995,978,323	7,670,529,472	1,735,089,360	1,629,831,914
Baseline per AE per day	0.10	0.10	0.09	0.11	0.08	0.07	0.08	0.09	0.10	0.10	0.09
Reformulated per AE per day	0.70	0.72	0.70	0.78	0.56	0.55	0.60	0.69	0.73	0.74	0.70
Change per AE per day	0.61	0.62	0.60	0.68	0.48	0.48	0.52	0.60	0.63	0.64	0.60
Percent change per AE per day	610.00%	620.00%	666.67%	618.18%	600.00%	685.71%	650.00%	666.67%	630.00%	640.00%	666.67%

Potential Improvements from Reformulation of Macaroni & Cheese Mix by Demographic Group for Consumer Network Households with Children (Weighted), 2014

	All Households	Low Income (<185% of Poverty Line)	High Income (>185% of Poverty Line)	Non-Hispanic White	Non-Hispanic Black	Hispanic	Asian and Other	Urban	Part Urban /Part Rural	Rural	Unspecified
**1-Star Targets**											
**Calories**											
Baseline (2014 average product)	342,422,841,273	142,748,774,961	199,674,066,312	255,388,691,041	22,495,336,337	46,315,069,942	18,223,743,953	207,100,349,158	94,828,030,337	20,538,836,047	19,955,625,731
Reformulated healthier product	347,169,020,075	144,727,355,617	202,441,664,459	258,928,526,139	22,807,134,721	46,957,023,622	18,476,335,593	209,970,879,884	96,142,401,731	20,823,516,205	20,232,222,255
Total change	4,746,178,803	1,978,580,656	2,767,598,147	3,539,835,098	311,798,384	641,953,680	252,591,641	2,870,530,726	1,314,371,395	284,680,157	276,596,525
Baseline per AE per day	7.18	7.93	6.73	8.33	4.61	5.35	5.24	6.88	7.80	7.61	7.37
Reformulated per AE per day	7.28	8.04	6.83	8.45	4.67	5.43	5.31	6.98	7.91	7.71	7.47
Change per AE per day	0.10	0.11	0.09	0.12	0.06	0.07	0.07	0.10	0.11	0.11	0.10
Percent change per AE per day	1.39%	1.39%	1.34%	1.44%	1.30%	1.31%	1.34%	1.45%	1.41%	1.45%	1.36%
**Saturated Fat (g)**											
Baseline (2014 average product)	2,512,682,896	1,047,483,877	1,465,199,019	1,874,030,346	165,069,733	339,857,831	133,724,986	1,519,692,737	695,843,680	150,713,024	146,433,454
Reformulated healthier product	2,512,682,896	1,047,483,877	1,465,199,019	1,874,030,346	165,069,733	339,857,831	133,724,986	1,519,692,737	695,843,680	150,713,024	146,433,454
Total change	0	0	0	0	0	0	0	0	0	0	0
Baseline per AE per day	0.05	0.06	0.05	0.06	0.03	0.04	0.04	0.05	0.06	0.06	0.05
Reformulated per AE per day	0.05	0.06	0.05	0.06	0.03	0.04	0.04	0.05	0.06	0.06	0.05
Change per AE per day	0.00	0.00	0.00	0.00	0.00	0.00	0.00	0.00	0.00	0.00	0.00
Percent change per AE per day	0.00%	0.00%	0.00%	0.00%	0.00%	0.00%	0.00%	0.00%	0.00%	0.00%	0.00%
**Sugars (g)**											
Baseline (2014 average product)	6,560,894,227	2,735,096,789	3,825,797,439	4,893,301,459	431,015,413	887,406,558	349,170,797	3,968,086,592	1,816,925,163	393,528,453	382,354,019
Reformulated healthier product	6,421,300,733	2,676,903,240	3,744,397,493	4,789,188,662	421,844,872	868,525,568	341,741,631	3,883,659,218	1,778,267,181	385,155,507	374,218,827
Total change	-139,593,494	-58,193,549	-81,399,946	-104,112,797	-9,170,541	-18,880,991	-7,429,166	-84,427,374	-38,657,982	-8,372,946	-8,135,192
Baseline per AE per day	0.14	0.15	0.13	0.16	0.09	0.10	0.10	0.13	0.15	0.15	0.14
Reformulated per AE per day	0.13	0.15	0.13	0.16	0.09	0.10	0.10	0.13	0.15	0.14	0.14
Change per AE per day	0.00	0.00	0.00	0.00	0.00	0.00	0.00	0.00	0.00	0.00	0.00
Percent change per AE per day	0.00%	0.00%	0.00%	0.00%	0.00%	0.00%	0.00%	0.00%	0.00%	0.00%	0.00%
**Sodium (mg)**											
Baseline (2014 average product)	822,764,054,815	342,992,776,038	479,771,278,777	613,640,825,518	54,051,166,886	111,284,558,596	43,787,503,815	497,614,944,124	227,850,147,087	49,350,142,545	47,948,821,059
Reformulated healthier product	759,528,201,942	316,631,098,477	442,897,103,466	566,477,728,477	49,896,911,949	102,731,469,855	40,422,091,662	459,369,343,566	210,338,081,150	45,557,198,098	44,263,579,128
Total change	-63,235,852,873	-26,361,677,561	-36,874,175,312	-47,163,097,041	-4,154,254,937	-8,553,088,742	-3,365,412,153	-38,245,600,558	-17,512,065,937	-3,792,944,447	-3,685,241,931
Baseline per AE per day	17.26	19.04	16.18	20.02	11.08	12.86	12.59	16.53	18.75	18.28	17.71
Reformulated per AE per day	15.94	17.58	14.94	18.48	10.23	11.87	11.62	15.26	17.31	16.88	16.35
Change per AE per day	-1.33	-1.46	-1.24	-1.54	-0.85	-0.99	-0.97	-1.27	-1.44	-1.41	-1.36
Percent change per AE per day	-7.71%	-7.67%	-7.66%	-7.69%	-7.67%	-7.70%	-7.70%	-7.68%	-7.68%	-7.71%	-7.68%
**Dietary Fiber (g)**											
Baseline (2014 average product)	2,233,495,907	931,096,779	1,302,399,128	1,665,804,752	146,728,651	302,095,850	118,866,654	1,350,837,989	618,527,715	133,967,133	130,163,070
Reformulated healthier product	3,629,430,849	1,513,032,266	2,116,398,583	2,706,932,722	238,434,058	490,905,756	193,158,313	2,195,111,732	1,005,107,537	217,696,591	211,514,989
Total change	1,395,934,942	581,935,487	813,999,455	1,041,127,970	91,705,407	188,809,906	74,291,659	844,273,743	386,579,822	83,729,458	81,351,919
Baseline per AE per day	0.05	0.05	0.04	0.05	0.03	0.03	0.03	0.04	0.05	0.05	0.05
Reformulated per AE per day	0.08	0.08	0.07	0.09	0.05	0.06	0.06	0.07	0.08	0.08	0.08
Change per AE per day	0.03	0.03	0.03	0.03	0.02	0.02	0.02	0.03	0.03	0.03	0.03
Percent change per AE per day	60.00%	60.00%	75.00%	60.00%	66.67%	66.67%	66.67%	75.00%	60.00%	60.00%	60.00%

Potential Improvements from Reformulation of Frozen Pizza by Demographic Group for Consumer Network Households with Children (Weighted), 2014

	All Households	Low Income (<185% of Poverty Line)	High Income (>185% of Poverty Line)	Non-Hispanic White	Non-Hispanic Black	Hispanic	Asian and Other	Urban	Part Urban /Part Rural	Rural	Unspecified
**1-Star Targets**											
**Calories**											
Baseline (2014 average product)	539,085,542,331	224,957,292,209	314,128,250,122	394,471,616,377	44,427,170,342	73,942,959,470	26,243,795,805	305,792,069,168	161,122,601,907	38,713,446,215	33,457,425,041
Reformulated healthier product	535,246,357,460	223,355,222,472	311,891,134,988	391,662,322,967	44,110,775,064	73,416,362,726	26,056,896,369	303,614,321,494	159,975,141,241	38,437,742,147	33,219,152,578
Total change	-3,839,184,871	-1,602,069,737	-2,237,115,134	-2,809,293,410	-316,395,278	-526,596,744	-186,899,436	-2,177,747,674	-1,147,460,666	-275,704,068	-238,272,463
Baseline per AE per day	11.31	12.49	10.59	12.87	9.11	8.55	7.55	10.16	13.26	14.34	12.36
Reformulated per AE per day	11.23	12.40	10.52	12.78	9.04	8.49	7.49	10.09	13.17	14.24	12.27
Change per AE per day	-0.08	-0.09	-0.08	-0.09	-0.06	-0.06	-0.05	-0.07	-0.09	-0.10	-0.09
Percent change per AE per day	-0.71%	-0.72%	-0.76%	-0.70%	-0.66%	-0.70%	-0.66%	-0.69%	-0.68%	-0.70%	-0.73%
**Saturated Fat (g)**											
Baseline (2014 average product)	9,437,996,142	3,938,421,436	5,499,574,705	6,906,179,634	777,805,059	1,294,550,329	459,461,114	5,353,629,698	2,820,840,805	677,772,501	585,753,139
Reformulated healthier product	7,518,403,706	3,137,386,568	4,381,017,138	5,501,532,929	619,607,420	1,031,251,957	366,011,396	4,264,755,861	2,247,110,472	539,920,467	466,616,907
Total change	-1,919,592,436	-801,034,868	-1,118,557,567	-1,404,646,705	-158,197,639	-263,298,372	-93,449,718	-1,088,873,837	-573,730,333	-137,852,034	-119,136,232
Baseline per AE per day	0.20	0.22	0.19	0.23	0.16	0.15	0.13	0.18	0.23	0.25	0.22
Reformulated per AE per day	0.16	0.17	0.15	0.18	0.13	0.12	0.11	0.14	0.18	0.20	0.17
Change per AE per day	-0.04	-0.04	-0.04	-0.05	-0.03	-0.03	-0.03	-0.04	-0.05	-0.05	-0.04
Percent change per AE per day	-20.00%	-18.18%	-21.05%	-21.74%	-18.75%	-20.00%	-23.08%	-22.22%	-21.74%	-20.00%	-18.18%
**Sugars (g)**											
Baseline (2014 average product)	6,718,573,525	2,803,622,039	3,914,951,485	4,916,263,468	553,691,737	921,544,302	327,074,013	3,811,058,429	2,008,056,166	482,482,119	416,976,811
Reformulated healthier product	4,639,015,053	1,935,834,265	2,703,180,787	3,394,562,871	382,310,961	636,304,399	225,836,819	2,631,445,106	1,386,514,972	333,142,416	287,912,560
Total change	-2,079,558,472	-867,787,774	-1,211,770,698	-1,521,700,597	-171,380,776	-285,239,903	-101,237,195	-1,179,613,323	-621,541,194	-149,339,704	-129,064,251
Baseline per AE per day	0.14	0.16	0.13	0.16	0.11	0.11	0.09	0.13	0.17	0.18	0.15
Reformulated per AE per day	0.10	0.11	0.09	0.11	0.08	0.07	0.06	0.09	0.11	0.12	0.11
Change per AE per day	-0.04	-0.05	-0.04	-0.05	-0.04	-0.03	-0.03	-0.04	-0.05	-0.06	-0.05
Percent change per AE per day	-28.57%	-31.25%	-30.77%	-31.25%	-36.36%	-27.27%	-33.33%	-30.77%	-29.41%	-33.33%	-33.33%
**Sodium (mg)**											
Baseline (2014 average product)	1,173,510,842,297	489,699,316,215	683,811,526,082	858,707,352,446	96,711,490,098	160,963,071,416	57,128,927,604	665,664,872,230	350,740,477,030	84,273,543,452	72,831,949,585
Reformulated healthier product	936,441,176,500	390,771,509,968	545,669,666,532	685,233,484,353	77,174,081,656	128,445,722,474	45,587,887,431	531,188,953,386	279,884,780,879	67,248,817,253	58,118,624,982
Total change	-237,069,665,797	-98,927,806,247	-138,141,859,549	-173,473,868,092	-19,537,408,441	-32,517,348,942	-11,541,040,173	-134,475,918,845	-70,855,696,150	-17,024,726,199	-14,713,324,603
Baseline per AE per day	24.62	27.19	23.06	28.02	19.82	18.60	16.43	22.11	28.87	31.22	26.90
Reformulated per AE per day	19.65	21.70	18.40	22.36	15.82	14.85	13.11	17.65	23.03	24.91	21.47
Change per AE per day	-4.97	-5.49	-4.66	-5.66	-4.00	-3.76	-3.32	-4.47	-5.83	-6.31	-5.43
Percent change per AE per day	-20.19%	-20.19%	-20.21%	-20.20%	-20.18%	-20.22%	-20.21%	-20.22%	-20.19%	-20.21%	-20.19%
**Dietary Fiber (g)**											
Baseline (2014 average product)	3,679,218,835	1,535,316,831	2,143,902,004	2,692,239,518	303,212,142	504,655,213	179,111,960	2,087,008,187	1,099,649,805	264,216,399	228,344,444
Reformulated healthier product	8,478,199,924	3,537,904,002	4,940,295,922	6,203,856,281	698,706,240	1,162,901,143	412,736,255	4,809,192,779	2,533,975,638	608,846,484	526,185,023
Total change	4,798,981,089	2,002,587,171	2,796,393,918	3,511,616,763	395,494,098	658,245,930	233,624,295	2,722,184,592	1,434,325,833	344,630,085	297,840,579
Baseline per AE per day	0.08	0.09	0.07	0.09	0.06	0.06	0.05	0.07	0.09	0.10	0.08
Reformulated per AE per day	0.18	0.20	0.17	0.20	0.14	0.13	0.12	0.16	0.21	0.23	0.19
Change per AE per day	0.10	0.11	0.09	0.11	0.08	0.08	0.07	0.09	0.12	0.13	0.11
Percent change per AE per day	125.00%	122.22%	128.57%	122.22%	133.33%	133.33%	140.00%	128.57%	133.33%	130.00%	137.50%

Potential Improvements from Reformulation of Potato Chips by Demographic Group for Consumer Network Households with Children (Weighted), 2014

	All Households	Low Income (<185% of Poverty Line)	High Income (>185% of Poverty Line)	Non-Hispanic White	Non-Hispanic Black	Hispanic	Asian and Other	Urban	Part Urban /Part Rural	Rural	Unspecified
**1-Star Targets**											
**Calories**											
Baseline (2014 average product)	575,531,150,604	225,923,364,946	349,607,785,658	404,758,934,448	58,708,647,991	77,757,790,691	34,305,777,475	344,431,200,217	159,742,047,558	38,623,018,894	32,734,883,936
Reformulated healthier product	566,316,509,100	222,306,179,650	344,010,329,450	398,278,471,188	57,768,682,980	76,512,836,070	33,756,518,863	338,916,624,630	157,184,469,745	38,004,638,338	32,210,776,388
Total change	-9,214,641,504	-3,617,185,296	-5,597,456,208	-6,480,463,260	-939,965,011	-1,244,954,621	-549,258,612	-5,514,575,587	-2,557,577,813	-618,380,556	-524,107,548
Baseline per AE per day	12.08	12.54	11.79	13.21	12.03	8.99	9.86	11.44	13.15	14.31	12.09
Reformulated per AE per day	11.88	12.34	11.60	12.99	11.84	8.84	9.71	11.26	12.94	14.08	11.90
Change per AE per day	-0.19	-0.20	-0.19	-0.21	-0.19	-0.14	-0.16	-0.18	-0.21	-0.23	-0.19
Percent change per AE per day	-1.57%	-1.59%	-1.61%	-1.59%	-1.58%	-1.56%	-1.62%	-1.57%	-1.60%	-1.61%	-1.57%
**Saturated Fat (g)**											
Baseline (2014 average product)	6,910,981,128	2,712,888,972	4,198,092,156	4,860,347,445	704,973,758	933,715,966	411,943,959	4,135,931,690	1,918,183,360	463,785,417	393,080,661
Reformulated healthier product	4,607,320,752	1,808,592,648	2,798,728,104	3,240,231,630	469,982,506	622,477,310	274,629,306	2,757,287,794	1,278,788,906	309,190,278	262,053,774
Total change	-2,303,660,376	-904,296,324	-1,399,364,052	-1,620,115,815	-234,991,253	-311,238,655	-137,314,653	-1,378,643,897	-639,394,453	-154,595,139	-131,026,887
Baseline per AE per day	0.15	0.15	0.14	0.16	0.14	0.11	0.12	0.14	0.16	0.17	0.15
Reformulated per AE per day	0.10	0.10	0.09	0.11	0.10	0.07	0.08	0.09	0.11	0.11	0.10
Change per AE per day	-0.05	-0.05	-0.05	-0.05	-0.05	-0.04	-0.04	-0.05	-0.05	-0.06	-0.05
Percent change per AE per day	-33.33%	-33.33%	-35.71%	-31.25%	-35.71%	-36.36%	-33.33%	-35.71%	-31.25%	-35.29%	-33.33%
**Sugars (g)**											
Baseline (2014 average product)	3,455,490,564	1,356,444,486	2,099,046,078	2,430,173,723	352,486,879	466,857,983	205,971,980	2,067,965,845	959,091,680	231,892,709	196,540,331
Reformulated healthier product	1,919,716,980	753,580,270	1,166,136,710	1,350,096,513	195,826,044	259,365,546	114,428,878	1,148,869,914	532,828,711	128,829,283	109,189,073
Total change	-1,535,773,584	-602,864,216	-932,909,368	-1,080,077,210	-156,660,835	-207,492,437	-91,543,102	-919,095,931	-426,262,969	-103,063,426	-87,351,258
Baseline per AE per day	0.07	0.08	0.07	0.08	0.07	0.05	0.06	0.07	0.08	0.09	0.07
Reformulated per AE per day	0.04	0.04	0.04	0.04	0.04	0.03	0.03	0.04	0.04	0.05	0.04
Change per AE per day	-0.03	-0.03	-0.03	-0.04	-0.03	-0.02	-0.03	-0.03	-0.04	-0.04	-0.03
Percent change per AE per day	-42.86%	-37.50%	-42.86%	-50.00%	-42.86%	-40.00%	-50.00%	-42.86%	-50.00%	-44.44%	-42.86%
**Sodium (mg)**											
Baseline (2014 average product)	741,010,754,280	290,881,984,220	450,128,770,060	521,137,253,825	75,588,852,984	100,115,100,756	44,169,546,715	443,463,786,804	205,671,882,446	49,728,103,045	42,146,981,985
Reformulated healthier product	518,707,527,996	203,617,388,954	315,090,139,042	364,796,077,678	52,912,197,089	70,080,570,529	30,918,682,701	310,424,650,763	143,970,317,712	34,809,672,132	29,502,887,390
Total change	-222,303,226,284	-87,264,595,266	-135,038,631,018	-156,341,176,148	-22,676,655,895	-30,034,530,227	-13,250,864,015	-133,039,136,041	-61,701,564,734	-14,918,430,914	-12,644,094,596
Baseline per AE per day	15.55	16.15	15.18	17.00	15.49	11.57	12.70	14.73	16.93	18.42	15.57
Reformulated per AE per day	10.88	11.31	10.63	11.90	10.84	8.10	8.89	10.31	11.85	12.90	10.90
Change per AE per day	-4.66	-4.85	-4.55	-5.10	-4.65	-3.47	-3.81	-4.42	-5.08	-5.53	-4.67
Percent change per AE per day	-29.97%	-30.03%	-29.97%	-30.00%	-30.02%	-29.99%	-30.00%	-30.01%	-30.01%	-30.02%	-29.99%
**Dietary Fiber (g)**											
Baseline (2014 average product)	3,839,433,960	1,507,160,540	2,332,273,420	2,700,193,025	391,652,088	518,731,092	228,857,755	2,297,739,828	1,065,657,422	257,658,565	218,378,145
Reformulated healthier product	4,607,320,752	1,808,592,648	2,798,728,104	3,240,231,630	469,982,506	622,477,310	274,629,306	2,757,287,794	1,278,788,906	309,190,278	262,053,774
Total change	767,886,792	301,432,108	466,454,684	540,038,605	78,330,418	103,746,218	45,771,551	459,547,966	213,131,484	51,531,713	43,675,629
Baseline per AE per day	0.08	0.08	0.08	0.09	0.08	0.06	0.07	0.08	0.09	0.10	0.08
Reformulated per AE per day	0.10	0.10	0.09	0.11	0.10	0.07	0.08	0.09	0.11	0.11	0.10
Change per AE per day	0.02	0.02	0.02	0.02	0.02	0.01	0.01	0.02	0.02	0.02	0.02
Percent change per AE per day	25.00%	25.00%	25.00%	22.22%	25.00%	16.67%	14.29%	25.00%	22.22%	20.00%	25.00%

Potential Improvements from Reformulation of Potato Chips by Demographic Group for Consumer Network Households with Children (Weighted), 2014

	All Households	Low Income (<185% of Poverty Line)	High Income (>185% of Poverty Line)	Non-Hispanic White	Non-Hispanic Black	Hispanic	Asian and Other	Urban	Part Urban /Part Rural	Rural	Unspecified
**2-Star Targets**											
**Calories**											
Baseline (2014 average product)	575,531,150,604	225,923,364,946	349,607,785,658	404,758,934,448	58,708,647,991	77,757,790,691	34,305,777,475	344,431,200,217	159,742,047,558	38,623,018,894	32,734,883,936
Reformulated healthier product	539,056,527,984	211,605,339,816	327,451,188,168	379,107,100,710	54,987,953,155	72,829,845,317	32,131,628,802	322,602,671,851	149,618,302,049	36,175,262,526	30,660,291,558
Total change	-36,474,622,620	-14,318,025,130	-22,156,597,490	-25,651,833,738	-3,720,694,836	-4,927,945,374	-2,174,148,673	-21,828,528,366	-10,123,745,509	-2,447,756,368	-2,074,592,378
Baseline per AE per day	12.08	12.54	11.79	13.21	12.03	8.99	9.86	11.44	13.15	14.31	12.09
Reformulated per AE per day	11.31	11.75	11.04	12.37	11.27	8.42	9.24	10.72	12.31	13.40	11.32
Change per AE per day	-0.77	-0.79	-0.75	-0.84	-0.76	-0.57	-0.63	-0.73	-0.83	-0.91	-0.77
Percent change per AE per day	-6.37%	-6.30%	-6.36%	-6.36%	-6.32%	-6.34%	-6.39%	-6.38%	-6.31%	-6.36%	-6.37%
**Saturated Fat (g)**											
Baseline (2014 average product)	6,910,981,128	2,712,888,972	4,198,092,156	4,860,347,445	704,973,758	933,715,966	411,943,959	4,135,931,690	1,918,183,360	463,785,417	393,080,661
Reformulated healthier product	3,455,490,564	1,356,444,486	2,099,046,078	2,430,173,723	352,486,879	466,857,983	205,971,980	2,067,965,845	959,091,680	231,892,709	196,540,331
Total change	-3,455,490,564	-1,356,444,486	-2,099,046,078	-2,430,173,723	-352,486,879	-466,857,983	-205,971,980	-2,067,965,845	-959,091,680	-231,892,709	-196,540,331
Baseline per AE per day	0.15	0.15	0.14	0.16	0.14	0.11	0.12	0.14	0.16	0.17	0.15
Reformulated per AE per day	0.07	0.08	0.07	0.08	0.07	0.05	0.06	0.07	0.08	0.09	0.07
Change per AE per day	-0.07	-0.08	-0.07	-0.08	-0.07	-0.05	-0.06	-0.07	-0.08	-0.09	-0.07
Percent change per AE per day	-46.67%	-53.33%	-50.00%	-50.00%	-50.00%	-45.45%	-50.00%	-50.00%	-50.00%	-52.94%	-46.67%
**Sugars (g)**											
Baseline (2014 average product)	3,455,490,564	1,356,444,486	2,099,046,078	2,430,173,723	352,486,879	466,857,983	205,971,980	2,067,965,845	959,091,680	231,892,709	196,540,331
Reformulated healthier product	1,151,830,188	452,148,162	699,682,026	810,057,908	117,495,626	155,619,328	68,657,327	689,321,948	319,697,227	77,297,570	65,513,444
Total change	-2,303,660,376	-904,296,324	-1,399,364,052	-1,620,115,815	-234,991,253	-311,238,655	-137,314,653	-1,378,643,897	-639,394,453	-154,595,139	-131,026,887
Baseline per AE per day	0.07	0.08	0.07	0.08	0.07	0.05	0.06	0.07	0.08	0.09	0.07
Reformulated per AE per day	0.02	0.03	0.02	0.03	0.02	0.02	0.02	0.02	0.03	0.03	0.02
Change per AE per day	-0.05	-0.05	-0.05	-0.05	-0.05	-0.04	-0.04	-0.05	-0.05	-0.06	-0.05
Percent change per AE per day	-71.43%	-62.50%	-71.43%	-62.50%	-71.43%	-80.00%	-66.67%	-71.43%	-62.50%	-66.67%	-71.43%
**Sodium (mg)**											
Baseline (2014 average product)	741,010,754,280	290,881,984,220	450,128,770,060	521,137,253,825	75,588,852,984	100,115,100,756	44,169,546,715	443,463,786,804	205,671,882,446	49,728,103,045	42,146,981,985
Reformulated healthier product	523,698,792,144	205,576,697,656	318,122,094,488	368,306,328,610	53,421,344,803	70,754,920,949	31,216,197,782	313,411,712,539	145,355,672,361	35,144,628,266	29,786,778,978
Total change	-217,311,962,136	-85,305,286,564	-132,006,675,572	-152,830,925,215	-22,167,508,181	-29,360,179,807	-12,953,348,933	-130,052,074,265	-60,316,210,085	-14,583,474,779	-12,360,203,007
Baseline per AE per day	15.55	16.15	15.18	17.00	15.49	11.57	12.70	14.73	16.93	18.42	15.57
Reformulated per AE per day	10.99	11.41	10.73	12.02	10.95	8.18	8.98	10.41	11.96	13.02	11.00
Change per AE per day	-4.56	-4.74	-4.45	-4.99	-4.54	-3.39	-3.72	-4.32	-4.96	-5.40	-4.57
Percent change per AE per day	-29.32%	-29.35%	-29.31%	-29.35%	-29.31%	-29.30%	-29.29%	-29.33%	-29.30%	-29.32%	-29.35%
**Dietary Fiber (g)**											
Baseline (2014 average product)	3,839,433,960	1,507,160,540	2,332,273,420	2,700,193,025	391,652,088	518,731,092	228,857,755	2,297,739,828	1,065,657,422	257,658,565	218,378,145
Reformulated healthier product	8,062,811,316	3,165,037,134	4,897,774,182	5,670,405,353	822,469,385	1,089,335,293	480,601,286	4,825,253,639	2,237,880,586	541,082,987	458,594,105
Total change	4,223,377,356	1,657,876,594	2,565,500,762	2,970,212,328	430,817,297	570,604,201	251,743,531	2,527,513,811	1,172,223,164	283,424,422	240,215,960
Baseline per AE per day	0.08	0.08	0.08	0.09	0.08	0.06	0.07	0.08	0.09	0.10	0.08
Reformulated per AE per day	0.17	0.18	0.17	0.19	0.17	0.13	0.14	0.16	0.18	0.20	0.17
Change per AE per day	0.09	0.09	0.09	0.10	0.09	0.07	0.07	0.08	0.10	0.11	0.09
Percent change per AE per day	112.50%	112.50%	112.50%	111.11%	112.50%	116.67%	100.00%	100.00%	111.11%	110.00%	112.50%

Potential Improvements from Reformulation of RTE Cereal by Demographic Group for Consumer Network Households with Children (Weighted), 2014

	All Households	Low Income (<185% of Poverty Line)	High Income (>185% of Poverty Line)	Non-Hispanic White	Non-Hispanic Black	Hispanic	Asian and Other	Urban	Part Urban /Part Rural	Rural	Unspecified
**1-Star Targets**											
**Calories**											
Baseline (2014 average product)	1,855,423,092,417	718,161,272,917	1,137,261,819,500	1,299,740,531,716	164,396,649,068	290,867,066,608	100,418,845,025	1,152,484,832,817	491,025,217,077	106,902,526,435	105,010,516,088
Reformulated healthier product	1,847,363,044,231	715,041,545,407	1,132,321,498,825	1,294,094,395,610	163,682,502,026	289,603,525,919	99,982,620,676	1,147,478,382,631	488,892,179,644	106,438,136,659	104,554,345,297
Total change	-8,060,048,186	-3,119,727,511	-4,940,320,676	-5,646,136,107	-714,147,042	-1,263,540,689	-436,224,349	-5,006,450,186	-2,133,037,433	-464,389,776	-456,170,791
Baseline per AE per day	38.93	39.87	38.36	42.40	33.69	33.62	28.87	38.29	40.41	39.61	38.79
Reformulated per AE per day	38.76	39.70	38.19	42.22	33.55	33.47	28.75	38.12	40.23	39.43	38.62
Change per AE per day	-0.17	-0.17	-0.17	-0.18	-0.15	-0.15	-0.13	-0.17	-0.18	-0.17	-0.17
Percent change per AE per day	-0.44%	-0.43%	-0.44%	-0.42%	-0.45%	-0.45%	-0.45%	-0.44%	-0.45%	-0.43%	-0.44%
**Saturated Fat (g)**											
Baseline (2014 average product)	3,224,019,274	1,247,891,004	1,976,128,270	2,258,454,443	285,658,817	505,416,276	174,489,739	2,002,580,074	853,214,973	185,755,910	182,468,316
Reformulated healthier product	3,224,019,274	1,247,891,004	1,976,128,270	2,258,454,443	285,658,817	505,416,276	174,489,739	2,002,580,074	853,214,973	185,755,910	182,468,316
Total change	0	0	0	0	0	0	0	0	0	0	0
Baseline per AE per day	0.07	0.07	0.07	0.07	0.06	0.06	0.05	0.07	0.07	0.07	0.07
Reformulated per AE per day	0.07	0.07	0.07	0.07	0.06	0.06	0.05	0.07	0.07	0.07	0.07
Change per AE per day	0.00	0.00	0.00	0.00	0.00	0.00	0.00	0.00	0.00	0.00	0.00
Percent change per AE per day	0.00%	0.00%	0.00%	0.00%	0.00%	0.00%	0.00%	0.00%	0.00%	0.00%	0.00%
**Sugars (g)**											
Baseline (2014 average product)	128,960,770,976	49,915,640,168	79,045,130,808	90,338,177,704	11,426,352,672	20,216,651,024	6,979,589,576	80,103,202,976	34,128,598,928	7,430,236,416	7,298,732,656
Reformulated healthier product	109,616,655,330	42,428,294,143	67,188,361,187	76,787,451,048	9,712,399,771	17,184,153,370	5,932,651,140	68,087,722,530	29,009,309,089	6,315,700,954	6,203,922,758
Total change	-19,344,115,646	-7,487,346,025	-11,856,769,621	-13,550,726,656	-1,713,952,901	-3,032,497,654	-1,046,938,436	-12,015,480,446	-5,119,289,839	-1,114,535,462	-1,094,809,898
Baseline per AE per day	2.71	2.77	2.67	2.95	2.34	2.34	2.01	2.66	2.81	2.75	2.70
Reformulated per AE per day	2.30	2.36	2.27	2.51	1.99	1.99	1.71	2.26	2.39	2.34	2.29
Change per AE per day	-0.41	-0.42	-0.40	-0.44	-0.35	-0.35	-0.30	-0.40	-0.42	-0.41	-0.40
Percent change per AE per day	-15.13%	-15.16%	-14.98%	-14.92%	-14.96%	-14.96%	-14.93%	-15.04%	-14.95%	-14.91%	-14.81%
**Sodium (mg)**											
Baseline (2014 average product)	2,148,808,846,388	831,719,354,299	1,317,089,492,088	1,505,259,885,993	190,391,601,397	336,859,947,687	116,297,411,310	1,334,719,619,588	568,667,779,638	123,806,314,282	121,615,132,881
Reformulated healthier product	1,919,903,477,905	743,119,093,001	1,176,784,384,904	1,344,909,620,568	170,109,825,404	300,975,392,120	103,908,639,813	1,192,536,434,305	508,089,516,541	110,617,644,643	108,659,882,416
Total change	-228,905,368,482	-88,600,261,298	-140,305,107,184	-160,350,265,425	-20,281,775,993	-35,884,555,568	-12,388,771,497	-142,183,185,282	-60,578,263,097	-13,188,669,638	-12,955,250,464
Baseline per AE per day	45.09	46.18	44.42	49.11	39.02	38.93	33.44	44.34	46.80	45.87	44.92
Reformulated per AE per day	40.28	41.26	39.69	43.88	34.86	34.79	29.88	39.62	41.81	40.98	40.14
Change per AE per day	-4.80	-4.92	-4.73	-5.23	-4.16	-4.15	-3.56	-4.72	-4.99	-4.89	-4.79
Percent change per AE per day	-10.65%	-10.65%	-10.65%	-10.65%	-10.66%	-10.66%	-10.65%	-10.65%	-10.66%	-10.66%	-10.66%
**Dietary Fiber (g)**											
Baseline (2014 average product)	32,240,192,744	12,478,910,042	19,761,282,702	22,584,544,426	2,856,588,168	5,054,162,756	1,744,897,394	20,025,800,744	8,532,149,732	1,857,559,104	1,824,683,164
Reformulated healthier product	41,912,250,567	16,222,583,055	25,689,667,513	29,359,907,754	3,713,564,618	6,570,411,583	2,268,366,612	26,033,540,967	11,091,794,652	2,414,826,835	2,372,088,113
Total change	9,672,057,823	3,743,673,013	5,928,384,811	6,775,363,328	856,976,450	1,516,248,827	523,469,218	6,007,740,223	2,559,644,920	557,267,731	547,404,949
Baseline per AE per day	0.68	0.69	0.67	0.74	0.59	0.58	0.50	0.67	0.70	0.69	0.67
Reformulated per AE per day	0.88	0.90	0.87	0.96	0.76	0.76	0.65	0.86	0.91	0.89	0.88
Change per AE per day	0.20	0.21	0.20	0.22	0.18	0.18	0.15	0.20	0.21	0.21	0.20
Percent change per AE per day	29.41%	30.43%	29.85%	29.73%	30.51%	31.03%	30.00%	29.85%	30.00%	30.43%	29.85%

Potential Improvements from Reformulation of RTE Cereal by Demographic Group for Consumer Network Households with Children (Weighted), 2014

	All Households	Low Income (<185% of Poverty Line)	High Income (>185% of Poverty Line)	Non-Hispanic White	Non-Hispanic Black	Hispanic	Asian and Other	Urban	Part Urban /Part Rural	Rural	Unspecified
**2-Star Targets**											
**Calories**											
Baseline (2014 average product)	1,855,423,092,417	718,161,272,917	1,137,261,819,500	1,299,740,531,716	164,396,649,068	290,867,066,608	100,418,845,025	1,152,484,832,817	491,025,217,077	106,902,526,435	105,010,516,088
Reformulated healthier product	1,861,871,130,966	720,657,054,926	1,141,214,076,041	1,304,257,440,602	164,967,966,702	291,877,899,159	100,767,824,504	1,156,489,992,966	492,731,647,023	107,274,038,256	105,375,452,721
Total change	6,448,038,549	2,495,782,008	3,952,256,540	4,516,908,885	571,317,634	1,010,832,551	348,979,479	4,005,160,149	1,706,429,946	371,511,821	364,936,633
Baseline per AE per day	38.93	39.87	38.36	42.40	33.69	33.62	28.87	38.29	40.41	39.61	38.79
Reformulated per AE per day	39.07	40.01	38.49	42.55	33.81	33.73	28.97	38.42	40.55	39.74	38.92
Change per AE per day	0.14	0.14	0.13	0.15	0.12	0.12	0.10	0.13	0.14	0.14	0.13
Percent change per AE per day	0.36%	0.35%	0.34%	0.35%	0.36%	0.36%	0.35%	0.34%	0.35%	0.35%	0.34%
**Saturated Fat (g)**											
Baseline (2014 average product)	3,224,019,274	1,247,891,004	1,976,128,270	2,258,454,443	285,658,817	505,416,276	174,489,739	2,002,580,074	853,214,973	185,755,910	182,468,316
Reformulated healthier product	3,224,019,274	1,247,891,004	1,976,128,270	2,258,454,443	285,658,817	505,416,276	174,489,739	2,002,580,074	853,214,973	185,755,910	182,468,316
Total change	0	0	0	0	0	0	0	0	0	0	0
Baseline per AE per day	0.07	0.07	0.07	0.07	0.06	0.06	0.05	0.07	0.07	0.07	0.07
Reformulated per AE per day	0.07	0.07	0.07	0.07	0.06	0.06	0.05	0.07	0.07	0.07	0.07
Change per AE per day	0.00	0.00	0.00	0.00	0.00	0.00	0.00	0.00	0.00	0.00	0.00
Percent change per AE per day	0.00%	0.00%	0.00%	0.00%	0.00%	0.00%	0.00%	0.00%	0.00%	0.00%	0.00%
**Sugars (g)**											
Baseline (2014 average product)	128,960,770,976	49,915,640,168	79,045,130,808	90,338,177,704	11,426,352,672	20,216,651,024	6,979,589,576	80,103,202,976	34,128,598,928	7,430,236,416	7,298,732,656
Reformulated healthier product	87,048,520,409	33,693,057,113	53,355,463,295	60,978,269,950	7,712,788,054	13,646,239,441	4,711,222,964	54,069,662,009	23,036,804,276	5,015,409,581	4,926,644,543
Total change	-41,912,250,567	-16,222,583,055	-25,689,667,513	-29,359,907,754	-3,713,564,618	-6,570,411,583	-2,268,366,612	-26,033,540,967	-11,091,794,652	-2,414,826,835	-2,372,088,113
Baseline per AE per day	2.71	2.77	2.67	2.95	2.34	2.34	2.01	2.66	2.81	2.75	2.70
Reformulated per AE per day	1.83	1.87	1.80	1.99	1.58	1.58	1.35	1.80	1.90	1.86	1.82
Change per AE per day	-0.88	-0.90	-0.87	-0.96	-0.76	-0.76	-0.65	-0.86	-0.91	-0.89	-0.88
Percent change per AE per day	-32.47%	-32.49%	-32.58%	-32.54%	-32.48%	-32.48%	-32.34%	-32.33%	-32.38%	-32.36%	-32.59%
**Sodium (mg)**											
Baseline (2014 average product)	2,148,808,846,388	831,719,354,299	1,317,089,492,088	1,505,259,885,993	190,391,601,397	336,859,947,687	116,297,411,310	1,334,719,619,588	568,667,779,638	123,806,314,282	121,615,132,881
Reformulated healthier product	1,200,947,179,714	464,839,399,065	736,107,780,650	841,274,279,869	106,407,909,258	188,267,562,661	64,997,427,927	745,961,077,714	317,822,577,517	69,194,076,624	67,969,447,859
Total change	-947,861,666,674	-366,879,955,235	-580,981,711,439	-663,985,606,124	-83,983,692,139	-148,592,385,026	-51,299,983,384	-588,758,541,874	-250,845,202,121	-54,612,237,658	-53,645,685,022
Baseline per AE per day	45.09	46.18	44.42	49.11	39.02	38.93	33.44	44.34	46.80	45.87	44.92
Reformulated per AE per day	25.20	25.81	24.83	27.45	21.81	21.76	18.69	24.78	26.16	25.64	25.11
Change per AE per day	-19.89	-20.37	-19.60	-21.66	-17.21	-17.17	-14.75	-19.56	-20.64	-20.23	-19.81
Percent change per AE per day	-44.11%	-44.11%	-44.12%	-44.11%	-44.11%	-44.10%	-44.11%	-44.11%	-44.10%	-44.10%	-44.10%
**Dietary Fiber (g)**											
Baseline (2014 average product)	32,240,192,744	12,478,910,042	19,761,282,702	22,584,544,426	2,856,588,168	5,054,162,756	1,744,897,394	20,025,800,744	8,532,149,732	1,857,559,104	1,824,683,164
Reformulated healthier product	56,420,337,302	21,838,092,574	34,582,244,729	39,522,952,746	4,999,029,294	8,844,784,823	3,053,570,440	35,045,151,302	14,931,262,031	3,250,728,432	3,193,195,537
Total change	24,180,144,558	9,359,182,532	14,820,962,027	16,938,408,320	2,142,441,126	3,790,622,067	1,308,673,046	15,019,350,558	6,399,112,299	1,393,169,328	1,368,512,373
Baseline per AE per day	0.68	0.69	0.67	0.74	0.59	0.58	0.50	0.67	0.70	0.69	0.67
Reformulated per AE per day	1.18	1.21	1.17	1.29	1.02	1.02	0.88	1.16	1.23	1.20	1.18
Change per AE per day	0.51	0.52	0.50	0.55	0.44	0.44	0.38	0.50	0.53	0.52	0.51
Percent change per AE per day	75.00%	75.36%	74.63%	74.32%	74.58%	75.86%	76.00%	74.63%	75.71%	75.36%	76.12%

Potential Improvements from Reformulation of RTE Cereal by Demographic Group for Consumer Network Households with Children (Weighted), 2014

	All Households	Low Income (<185% of Poverty Line)	High Income (>185% of Poverty Line)	Non-Hispanic White	Non-Hispanic Black	Hispanic	Asian and Other	Urban	Part Urban /Part Rural	Rural	Unspecified
**3-Star Targets**											
**Calories**											
Baseline (2014 average product)	1,855,423,092,417	718,161,272,917	1,137,261,819,500	1,299,740,531,716	164,396,649,068	290,867,066,608	100,418,845,025	1,152,484,832,817	491,025,217,077	106,902,526,435	105,010,516,088
Reformulated healthier product	1,716,790,263,618	664,501,959,737	1,052,288,303,882	1,202,626,990,685	152,113,319,946	269,134,166,757	92,915,786,231	1,066,373,889,618	454,336,973,229	98,915,022,288	97,164,378,483
Total change	-138,632,828,799	-53,659,313,181	-84,973,515,619	-97,113,541,032	-12,283,329,122	-21,732,899,851	-7,503,058,794	-86,110,943,199	-36,688,243,848	-7,987,504,147	-7,846,137,605
Baseline per AE per day	38.93	39.87	38.36	42.40	33.69	33.62	28.87	38.29	40.41	39.61	38.79
Reformulated per AE per day	36.02	36.89	35.49	39.24	31.18	31.11	26.72	35.42	37.39	36.65	35.89
Change per AE per day	-2.91	-2.98	-2.87	-3.17	-2.52	-2.51	-2.16	-2.86	-3.02	-2.96	-2.90
Percent change per AE per day	-7.47%	-7.47%	-7.48%	-7.48%	-7.48%	-7.47%	-7.48%	-7.47%	-7.47%	-7.47%	-7.48%
**Saturated Fat (g)**											
Baseline (2014 average product)	3,224,019,274	1,247,891,004	1,976,128,270	2,258,454,443	285,658,817	505,416,276	174,489,739	2,002,580,074	853,214,973	185,755,910	182,468,316
Reformulated healthier product	1,612,009,637	623,945,502	988,064,135	1,129,227,221	142,829,408	252,708,138	87,244,870	1,001,290,037	426,607,487	92,877,955	91,234,158
Total change	-1,612,009,637	-623,945,502	-988,064,135	-1,129,227,221	-142,829,408	-252,708,138	-87,244,870	-1,001,290,037	-426,607,487	-92,877,955	-91,234,158
Baseline per AE per day	0.07	0.07	0.07	0.07	0.06	0.06	0.05	0.07	0.07	0.07	0.07
Reformulated per AE per day	0.03	0.03	0.03	0.04	0.03	0.03	0.03	0.03	0.04	0.03	0.03
Change per AE per day	-0.03	-0.03	-0.03	-0.04	-0.03	-0.03	-0.03	-0.03	-0.04	-0.03	-0.03
Percent change per AE per day	-42.86%	-42.86%	-42.86%	-57.14%	-50.00%	-50.00%	-60.00%	-42.86%	-57.14%	-42.86%	-42.86%
**Sugars (g)**											
Baseline (2014 average product)	128,960,770,976	49,915,640,168	79,045,130,808	90,338,177,704	11,426,352,672	20,216,651,024	6,979,589,576	80,103,202,976	34,128,598,928	7,430,236,416	7,298,732,656
Reformulated healthier product	17,732,106,009	6,863,400,523	10,868,705,486	12,421,499,434	1,571,123,492	2,779,789,516	959,693,567	11,014,190,409	4,692,682,353	1,021,657,507	1,003,575,740
Total change	-111,228,664,967	-43,052,239,645	-68,176,425,322	-77,916,678,270	-9,855,229,180	-17,436,861,508	-6,019,896,009	-69,089,012,567	-29,435,916,575	-6,408,578,909	-6,295,156,916
Baseline per AE per day	2.71	2.77	2.67	2.95	2.34	2.34	2.01	2.66	2.81	2.75	2.70
Reformulated per AE per day	0.37	0.38	0.37	0.41	0.32	0.32	0.28	0.37	0.39	0.38	0.37
Change per AE per day	-2.33	-2.39	-2.30	-2.54	-2.02	-2.02	-1.73	-2.30	-2.42	-2.37	-2.33
Percent change per AE per day	-85.98%	-86.28%	-86.14%	-86.10%	-86.32%	-86.32%	-86.07%	-86.47%	-86.12%	-86.18%	-86.30%
**Sodium (mg)**											
Baseline (2014 average product)	2,148,808,846,388	831,719,354,299	1,317,089,492,088	1,505,259,885,993	190,391,601,397	336,859,947,687	116,297,411,310	1,334,719,619,588	568,667,779,638	123,806,314,282	121,615,132,881
Reformulated healthier product	876,933,242,637	339,426,353,142	537,506,889,494	614,299,608,387	77,699,198,170	137,473,226,963	47,461,209,117	544,701,780,237	232,074,472,710	50,525,607,629	49,631,382,061
Total change	-1,271,875,603,751	-492,293,001,157	-779,582,602,594	-890,960,277,606	-112,692,403,228	-199,386,720,724	-68,836,202,193	-790,017,839,351	-336,593,306,927	-73,280,706,653	-71,983,750,820
Baseline per AE per day	45.09	46.18	44.42	49.11	39.02	38.93	33.44	44.34	46.80	45.87	44.92
Reformulated per AE per day	18.40	18.85	18.13	20.04	15.92	15.89	13.65	18.09	19.10	18.72	18.33
Change per AE per day	-26.69	-27.33	-26.29	-29.07	-23.10	-23.04	-19.79	-26.24	-27.70	-27.15	-26.59
Percent change per AE per day	-59.19%	-59.18%	-59.19%	-59.19%	-59.20%	-59.18%	-59.18%	-59.18%	-59.19%	-59.19%	-59.19%
**Dietary Fiber (g)**											
Baseline (2014 average product)	32,240,192,744	12,478,910,042	19,761,282,702	22,584,544,426	2,856,588,168	5,054,162,756	1,744,897,394	20,025,800,744	8,532,149,732	1,857,559,104	1,824,683,164
Reformulated healthier product	41,912,250,567	16,222,583,055	25,689,667,513	29,359,907,754	3,713,564,618	6,570,411,583	2,268,366,612	26,033,540,967	11,091,794,652	2,414,826,835	2,372,088,113
Total change	9,672,057,823	3,743,673,013	5,928,384,811	6,775,363,328	856,976,450	1,516,248,827	523,469,218	6,007,740,223	2,559,644,920	557,267,731	547,404,949
Baseline per AE per day	0.68	0.69	0.67	0.74	0.59	0.58	0.50	0.67	0.70	0.69	0.67
Reformulated per AE per day	0.88	0.90	0.87	0.96	0.76	0.76	0.65	0.86	0.91	0.89	0.88
Change per AE per day	0.20	0.21	0.20	0.22	0.18	0.18	0.15	0.20	0.21	0.21	0.20
Percent change per AE per day	29.41%	30.43%	29.85%	29.73%	30.51%	31.03%	30.00%	29.85%	30.00%	30.43%	29.85%

Potential Improvements from Reformulation of Tortilla Chips and Other Chips by Demographic Group for Consumer Network Households with Children (Weighted), 2014

	All Households	Low Income (<185% of Poverty Line)	High Income (>185% of Poverty Line)	Non-Hispanic White	Non-Hispanic Black	Hispanic	Asian and Other	Urban	Part Urban /Part Rural	Rural	Unspecified
**1-Star Targets**											
**Calories**											
Baseline (2014 average product)	914,274,386,771	327,559,538,649	586,714,848,122	647,568,705,017	72,003,751,975	144,833,192,990	49,868,736,789	548,295,067,838	256,941,811,249	55,967,116,729	53,070,390,955
Reformulated healthier product	883,884,785,514	316,671,774,635	567,213,010,879	626,044,144,100	69,610,416,514	140,019,076,957	48,211,147,943	530,070,267,139	248,401,312,573	54,106,823,598	51,306,382,204
Total change	-30,389,601,258	-10,887,764,015	-19,501,837,243	-21,524,560,916	-2,393,335,462	-4,814,116,033	-1,657,588,847	-18,224,800,699	-8,540,498,677	-1,860,293,130	-1,764,008,752
Baseline per AE per day	19.18	18.19	19.79	21.13	14.76	16.74	14.34	18.21	21.15	20.74	19.60
Reformulated per AE per day	18.55	17.58	19.13	20.43	14.27	16.18	13.86	17.61	20.44	20.05	18.95
Change per AE per day	-0.64	-0.60	-0.66	-0.70	-0.49	-0.56	-0.48	-0.61	-0.70	-0.69	-0.65
Percent change per AE per day	-3.34%	-3.30%	-3.34%	-3.31%	-3.32%	-3.35%	-3.35%	-3.35%	-3.31%	-3.33%	-3.32%
**Saturated Fat (g)**											
Baseline (2014 average product)	9,698,808,912	3,474,818,303	6,223,990,610	6,869,540,718	763,830,467	1,536,420,011	529,017,717	5,816,425,755	2,725,691,067	593,710,574	562,981,517
Reformulated healthier product	5,819,285,347	2,084,890,982	3,734,394,366	4,121,724,431	458,298,280	921,852,006	317,410,630	3,489,855,453	1,635,414,640	356,226,344	337,788,910
Total change	-3,879,523,565	-1,389,927,321	-2,489,596,244	-2,747,816,287	-305,532,187	-614,568,004	-211,607,087	-2,326,570,302	-1,090,276,427	-237,484,229	-225,192,607
Baseline per AE per day	0.20	0.19	0.21	0.22	0.16	0.18	0.15	0.19	0.22	0.22	0.21
Reformulated per AE per day	0.12	0.12	0.13	0.13	0.09	0.11	0.09	0.12	0.13	0.13	0.12
Change per AE per day	-0.08	-0.08	-0.08	-0.09	-0.06	-0.07	-0.06	-0.08	-0.09	-0.09	-0.08
Percent change per AE per day	-40.00%	-42.11%	-38.10%	-40.91%	-37.50%	-38.89%	-40.00%	-42.11%	-40.91%	-40.91%	-38.10%
**Sugars (g)**											
Baseline (2014 average product)	5,172,698,086	1,853,236,428	3,319,461,658	3,663,755,050	407,376,249	819,424,006	282,142,782	3,102,093,736	1,453,701,902	316,645,639	300,256,809
Reformulated healthier product	7,759,047,130	2,779,854,642	4,979,192,488	5,495,632,574	611,064,373	1,229,136,008	423,214,174	4,653,140,604	2,180,552,854	474,968,459	450,385,213
Total change	2,586,349,043	926,618,214	1,659,730,829	1,831,877,525	203,688,124	409,712,003	141,071,391	1,551,046,868	726,850,951	158,322,820	150,128,404
Baseline per AE per day	0.11	0.10	0.11	0.12	0.08	0.09	0.08	0.10	0.12	0.12	0.11
Reformulated per AE per day	0.16	0.15	0.17	0.18	0.13	0.14	0.12	0.15	0.18	0.18	0.17
Change per AE per day	0.05	0.05	0.06	0.06	0.04	0.05	0.04	0.05	0.06	0.06	0.06
Percent change per AE per day	45.45%	50.00%	54.55%	50.00%	50.00%	55.56%	50.00%	50.00%	50.00%	50.00%	54.55%
**Sodium (mg)**											
Baseline (2014 average product)	1,375,937,690,982	492,960,889,848	882,976,801,134	974,558,843,194	108,362,082,181	217,966,785,490	75,049,980,118	825,156,933,776	386,684,706,038	84,227,740,027	79,868,311,141
Reformulated healthier product	860,607,644,125	308,332,210,709	552,275,433,416	609,557,246,377	67,777,223,394	136,331,668,932	46,941,505,422	516,110,845,327	241,859,654,012	52,681,918,222	49,955,226,564
Total change	-515,330,046,858	-184,628,679,140	-330,701,367,718	-365,001,596,816	-40,584,858,787	-81,635,116,558	-28,108,474,697	-309,046,088,449	-144,825,052,027	-31,545,821,805	-29,913,084,577
Baseline per AE per day	28.87	27.37	29.78	31.80	22.21	25.19	21.58	27.41	31.82	31.21	29.50
Reformulated per AE per day	18.06	17.12	18.63	19.89	13.89	15.76	13.50	17.15	19.90	19.52	18.45
Change per AE per day	-10.81	-10.25	-11.15	-11.91	-8.32	-9.44	-8.08	-10.27	-11.92	-11.69	-11.05
Percent change per AE per day	-37.44%	-37.45%	-37.44%	-37.45%	-37.46%	-37.48%	-37.44%	-37.47%	-37.46%	-37.46%	-37.46%
**Dietary Fiber (g)**											
Baseline (2014 average product)	8,405,634,390	3,011,509,196	5,394,125,195	5,953,601,956	661,986,404	1,331,564,009	458,482,021	5,040,902,321	2,362,265,591	514,549,164	487,917,314
Reformulated healthier product	15,518,094,259	5,559,709,284	9,958,384,975	10,991,265,149	1,222,128,746	2,458,272,017	846,428,347	9,306,281,208	4,361,105,707	949,936,918	900,770,426
Total change	7,112,459,869	2,548,200,089	4,564,259,780	5,037,663,193	560,142,342	1,126,708,008	387,946,326	4,265,378,887	1,998,840,116	435,387,754	412,853,112
Baseline per AE per day	0.18	0.17	0.18	0.19	0.14	0.15	0.13	0.17	0.19	0.19	0.18
Reformulated per AE per day	0.33	0.31	0.34	0.36	0.25	0.28	0.24	0.31	0.36	0.35	0.33
Change per AE per day	0.15	0.14	0.15	0.16	0.11	0.13	0.11	0.14	0.16	0.16	0.15
Percent change per AE per day	83.33%	82.35%	83.33%	84.21%	78.57%	86.67%	84.62%	82.35%	84.21%	84.21%	83.33%

Potential Improvements from Reformulation of Tortilla Chips and Other Chips by Demographic Group for Consumer Network Households with Children (Weighted), 2014

	All Households	Low Income (<185% of Poverty Line)	High Income (>185% of Poverty Line)	Non-Hispanic White	Non-Hispanic Black	Hispanic	Asian and Other	Urban	Part Urban /Part Rural	Rural	Unspecified
**2-Star Targets**											
**Calories**											
Baseline (2014 average product)	914,274,386,771	327,559,538,649	586,714,848,122	647,568,705,017	72,003,751,975	144,833,192,990	49,868,736,789	548,295,067,838	256,941,811,249	55,967,116,729	53,070,390,955
Reformulated healthier product	845,089,549,866	302,772,501,425	542,317,048,441	598,565,981,228	66,555,094,648	133,873,396,915	46,095,077,075	506,804,564,119	237,498,548,305	51,731,981,304	49,054,456,138
Total change	-69,184,836,906	-24,787,037,225	-44,397,799,681	-49,002,723,788	-5,448,657,328	-10,959,796,075	-3,773,659,715	-41,490,503,719	-19,443,262,945	-4,235,135,424	-4,015,934,818
Baseline per AE per day	19.18	18.19	19.79	21.13	14.76	16.74	14.34	18.21	21.15	20.74	19.60
Reformulated per AE per day	17.73	16.81	18.29	19.53	13.64	15.47	13.25	16.84	19.55	19.17	18.12
Change per AE per day	-1.45	-1.38	-1.50	-1.60	-1.12	-1.27	-1.09	-1.38	-1.60	-1.57	-1.48
Percent change per AE per day	-7.56%	-7.59%	-7.58%	-7.57%	-7.59%	-7.59%	-7.60%	-7.58%	-7.57%	-7.57%	-7.55%
**Saturated Fat (g)**											
Baseline (2014 average product)	9,698,808,912	3,474,818,303	6,223,990,610	6,869,540,718	763,830,467	1,536,420,011	529,017,717	5,816,425,755	2,725,691,067	593,710,574	562,981,517
Reformulated healthier product	3,879,523,565	1,389,927,321	2,489,596,244	2,747,816,287	305,532,187	614,568,004	211,607,087	2,326,570,302	1,090,276,427	237,484,229	225,192,607
Total change	-5,819,285,347	-2,084,890,982	-3,734,394,366	-4,121,724,431	-458,298,280	-921,852,006	-317,410,630	-3,489,855,453	-1,635,414,640	-356,226,344	-337,788,910
Baseline per AE per day	0.20	0.19	0.21	0.22	0.16	0.18	0.15	0.19	0.22	0.22	0.21
Reformulated per AE per day	0.08	0.08	0.08	0.09	0.06	0.07	0.06	0.08	0.09	0.09	0.08
Change per AE per day	-0.12	-0.12	-0.13	-0.13	-0.09	-0.11	-0.09	-0.12	-0.13	-0.13	-0.12
Percent change per AE per day	-60.00%	-63.16%	-61.90%	-59.09%	-56.25%	-61.11%	-60.00%	-63.16%	-59.09%	-59.09%	-57.14%
**Sugars (g)**											
Baseline (2014 average product)	5,172,698,086	1,853,236,428	3,319,461,658	3,663,755,050	407,376,249	819,424,006	282,142,782	3,102,093,736	1,453,701,902	316,645,639	300,256,809
Reformulated healthier product	7,759,047,130	2,779,854,642	4,979,192,488	5,495,632,574	611,064,373	1,229,136,008	423,214,174	4,653,140,604	2,180,552,854	474,968,459	450,385,213
Total change	2,586,349,043	926,618,214	1,659,730,829	1,831,877,525	203,688,124	409,712,003	141,071,391	1,551,046,868	726,850,951	158,322,820	150,128,404
Baseline per AE per day	0.11	0.10	0.11	0.12	0.08	0.09	0.08	0.10	0.12	0.12	0.11
Reformulated per AE per day	0.16	0.15	0.17	0.18	0.13	0.14	0.12	0.15	0.18	0.18	0.17
Change per AE per day	0.05	0.05	0.06	0.06	0.04	0.05	0.04	0.05	0.06	0.06	0.06
Percent change per AE per day	45.45%	50.00%	54.55%	50.00%	50.00%	55.56%	50.00%	50.00%	50.00%	50.00%	54.55%
**Sodium (mg)**											
Baseline (2014 average product)	1,375,937,690,982	492,960,889,848	882,976,801,134	974,558,843,194	108,362,082,181	217,966,785,490	75,049,980,118	825,156,933,776	386,684,706,038	84,227,740,027	79,868,311,141
Reformulated healthier product	833,450,979,171	298,602,719,462	534,848,259,710	590,322,532,367	65,638,498,088	132,029,692,902	45,460,255,814	499,824,853,213	234,227,719,024	51,019,528,616	48,378,878,318
Total change	-542,486,711,811	-194,358,170,387	-348,128,541,425	-384,236,310,827	-42,723,584,093	-85,937,092,587	-29,589,724,304	-325,332,080,563	-152,456,987,014	-33,208,211,411	-31,489,432,823
Baseline per AE per day	28.87	27.37	29.78	31.80	22.21	25.19	21.58	27.41	31.82	31.21	29.50
Reformulated per AE per day	17.49	16.58	18.04	19.26	13.45	15.26	13.07	16.60	19.28	18.90	17.87
Change per AE per day	-11.38	-10.79	-11.74	-12.54	-8.76	-9.93	-8.51	-10.81	-12.55	-12.30	-11.63
Percent change per AE per day	-39.42%	-39.42%	-39.42%	-39.43%	-39.44%	-39.42%	-39.43%	-39.44%	-39.44%	-39.41%	-39.42%
**Dietary Fiber (g)**											
Baseline (2014 average product)	8,405,634,390	3,011,509,196	5,394,125,195	5,953,601,956	661,986,404	1,331,564,009	458,482,021	5,040,902,321	2,362,265,591	514,549,164	487,917,314
Reformulated healthier product	19,397,617,824	6,949,636,605	12,447,981,219	13,739,081,436	1,527,660,933	3,072,840,021	1,058,035,434	11,632,851,510	5,451,382,134	1,187,421,147	1,125,963,033
Total change	10,991,983,434	3,938,127,410	7,053,856,024	7,785,479,480	865,674,529	1,741,276,012	599,553,413	6,591,949,189	3,089,116,543	672,871,983	638,045,719
Baseline per AE per day	0.18	0.17	0.18	0.19	0.14	0.15	0.13	0.17	0.19	0.19	0.18
Reformulated per AE per day	0.41	0.39	0.42	0.45	0.31	0.36	0.30	0.39	0.45	0.44	0.42
Change per AE per day	0.23	0.22	0.24	0.25	0.18	0.20	0.17	0.22	0.25	0.25	0.24
Percent change per AE per day	127.78%	129.41%	133.33%	131.58%	128.57%	133.33%	130.77%	129.41%	131.58%	131.58%	133.33%

Potential Improvements from Reformulation of Tortilla Chips and Other Chips by Demographic Group for Consumer Network Households with Children (Weighted), 2014

	All Households	Low Income (<185% of Poverty Line)	High Income (>185% of Poverty Line)	Non-Hispanic White	Non-Hispanic Black	Hispanic	Asian and Other	Urban	Part Urban /Part Rural	Rural	Unspecified
**3-Star Targets**											
**Calories**											
Baseline (2014 average product)	914,274,386,771	327,559,538,649	586,714,848,122	647,568,705,017	72,003,751,975	144,833,192,990	49,868,736,789	548,295,067,838	256,941,811,249	55,967,116,729	53,070,390,955
Reformulated healthier product	492,052,905,469	176,289,115,214	315,763,790,255	348,514,699,093	38,751,665,667	77,947,708,533	26,838,832,176	295,086,666,637	138,283,393,466	30,120,916,429	28,561,928,937
Total change	-422,221,481,302	-151,270,423,436	-270,951,057,867	-299,054,005,924	-33,252,086,308	-66,885,484,457	-23,029,904,613	-253,208,401,201	-118,658,417,783	-25,846,200,300	-24,508,462,018
Baseline per AE per day	19.18	18.19	19.79	21.13	14.76	16.74	14.34	18.21	21.15	20.74	19.60
Reformulated per AE per day	10.32	9.79	10.65	11.37	7.94	9.01	7.72	9.80	11.38	11.16	10.55
Change per AE per day	-8.86	-8.40	-9.14	-9.76	-6.82	-7.73	-6.62	-8.41	-9.77	-9.58	-9.05
Percent change per AE per day	-46.19%	-46.18%	-46.18%	-46.19%	-46.21%	-46.18%	-46.16%	-46.18%	-46.19%	-46.19%	-46.17%
**Saturated Fat (g)**											
Baseline (2014 average product)	9,698,808,912	3,474,818,303	6,223,990,610	6,869,540,718	763,830,467	1,536,420,011	529,017,717	5,816,425,755	2,725,691,067	593,710,574	562,981,517
Reformulated healthier product	3,232,936,304	1,158,272,768	2,074,663,537	2,289,846,906	254,610,156	512,140,004	176,339,239	1,938,808,585	908,563,689	197,903,525	187,660,506
Total change	-6,465,872,608	-2,316,545,535	-4,149,327,073	-4,579,693,812	-509,220,311	-1,024,280,007	-352,678,478	-3,877,617,170	-1,817,127,378	-395,807,049	-375,321,011
Baseline per AE per day	0.20	0.19	0.21	0.22	0.16	0.18	0.15	0.19	0.22	0.22	0.21
Reformulated per AE per day	0.07	0.06	0.07	0.07	0.05	0.06	0.05	0.06	0.07	0.07	0.07
Change per AE per day	-0.14	-0.13	-0.14	-0.15	-0.10	-0.12	-0.10	-0.13	-0.15	-0.15	-0.14
Percent change per AE per day	-70.00%	-68.42%	-66.67%	-68.18%	-62.50%	-66.67%	-66.67%	-68.42%	-68.18%	-68.18%	-66.67%
**Sugars (g)**											
Baseline (2014 average product)	5,172,698,086	1,853,236,428	3,319,461,658	3,663,755,050	407,376,249	819,424,006	282,142,782	3,102,093,736	1,453,701,902	316,645,639	300,256,809
Reformulated healthier product	13,578,332,477	4,864,745,624	8,713,586,853	9,617,357,005	1,069,362,653	2,150,988,015	740,624,804	8,142,996,057	3,815,967,494	831,194,803	788,174,123
Total change	8,405,634,390	3,011,509,196	5,394,125,195	5,953,601,956	661,986,404	1,331,564,009	458,482,021	5,040,902,321	2,362,265,591	514,549,164	487,917,314
Baseline per AE per day	0.11	0.10	0.11	0.12	0.08	0.09	0.08	0.10	0.12	0.12	0.11
Reformulated per AE per day	0.28	0.27	0.29	0.31	0.22	0.25	0.21	0.27	0.31	0.31	0.29
Change per AE per day	0.18	0.17	0.18	0.19	0.14	0.15	0.13	0.17	0.19	0.19	0.18
Percent change per AE per day	163.64%	170.00%	163.64%	158.33%	175.00%	166.67%	162.50%	170.00%	158.33%	158.33%	163.64%
**Sodium (mg)**											
Baseline (2014 average product)	1,375,937,690,982	492,960,889,848	882,976,801,134	974,558,843,194	108,362,082,181	217,966,785,490	75,049,980,118	825,156,933,776	386,684,706,038	84,227,740,027	79,868,311,141
Reformulated healthier product	512,743,697,814	183,702,060,926	329,041,636,889	363,169,719,292	40,381,170,662	81,225,404,555	27,967,403,305	307,495,041,581	144,098,201,075	31,387,498,986	29,762,956,172
Total change	-863,193,993,168	-309,258,828,923	-553,935,164,246	-611,389,123,902	-67,980,911,519	-136,741,380,935	-47,082,576,813	-517,661,892,195	-242,586,504,963	-52,840,241,042	-50,105,354,969
Baseline per AE per day	28.87	27.37	29.78	31.80	22.21	25.19	21.58	27.41	31.82	31.21	29.50
Reformulated per AE per day	10.76	10.20	11.10	11.85	8.28	9.39	8.04	10.21	11.86	11.63	10.99
Change per AE per day	-18.11	-17.17	-18.68	-19.95	-13.93	-15.80	-13.54	-17.20	-19.96	-19.58	-18.51
Percent change per AE per day	-62.73%	-62.73%	-62.73%	-62.74%	-62.72%	-62.72%	-62.74%	-62.75%	-62.73%	-62.74%	-62.75%
**Dietary Fiber (g)**											
Baseline (2014 average product)	8,405,634,390	3,011,509,196	5,394,125,195	5,953,601,956	661,986,404	1,331,564,009	458,482,021	5,040,902,321	2,362,265,591	514,549,164	487,917,314
Reformulated healthier product	24,570,315,910	8,802,873,033	15,767,442,877	17,402,836,486	1,935,037,182	3,892,264,027	1,340,178,216	14,734,945,246	6,905,084,036	1,504,066,786	1,426,219,842
Total change	16,164,681,520	5,791,363,838	10,373,317,683	11,449,234,530	1,273,050,778	2,560,700,018	881,696,195	9,694,042,925	4,542,818,445	989,517,623	938,302,528
Baseline per AE per day	0.18	0.17	0.18	0.19	0.14	0.15	0.13	0.17	0.19	0.19	0.18
Reformulated per AE per day	0.52	0.49	0.53	0.57	0.40	0.45	0.39	0.49	0.57	0.56	0.53
Change per AE per day	0.34	0.32	0.35	0.37	0.26	0.30	0.25	0.32	0.37	0.37	0.35
Percent change per AE per day	188.89%	188.24%	194.44%	194.74%	185.71%	200.00%	192.31%	188.24%	194.74%	194.74%	194.44%

Potential Improvements from Reformulation of Yeast Breads by Demographic Group for Consumer Network Households with Children (Weighted), 2014

	All Households	Low Income (<185% of Poverty Line)	High Income (>185% of Poverty Line)	Non-Hispanic White	Non-Hispanic Black	Hispanic	Asian and Other	Urban	Part Urban /Part Rural	Rural	Unspecified
**1-Star Targets**											
**Calories**											
Baseline (2014 average product)	1,344,402,120,562	537,237,149,602	807,164,970,961	920,527,550,151	124,047,574,598	214,285,061,724	85,541,934,089	831,388,362,134	351,072,772,048	84,705,154,556	77,235,831,824
Reformulated healthier product	1,512,336,408,914	604,345,447,792	907,990,961,122	1,035,514,083,331	139,542,820,286	241,052,208,841	96,227,296,456	935,240,186,528	394,926,582,773	95,285,991,668	86,883,647,945
Total change	167,934,288,352	67,108,298,190	100,825,990,161	114,986,533,180	15,495,245,688	26,767,147,117	10,685,362,367	103,851,824,394	43,853,810,725	10,580,837,112	9,647,816,122
Baseline per AE per day	28.21	29.83	27.22	30.03	25.42	24.77	24.60	27.62	28.89	31.38	28.53
Reformulated per AE per day	31.73	33.55	30.62	33.78	28.60	27.86	27.67	31.07	32.50	35.30	32.09
Change per AE per day	3.52	3.73	3.40	3.75	3.18	3.09	3.07	3.45	3.61	3.92	3.56
Percent change per AE per day	12.48%	12.50%	12.49%	12.49%	12.51%	12.47%	12.48%	12.49%	12.50%	12.49%	12.48%
**Saturated Fat (g)**											
Baseline (2014 average product)	927,813,748	370,764,078	557,049,669	635,284,714	85,609,092	147,884,791	59,035,151	573,766,986	242,286,247	58,457,664	53,302,852
Reformulated healthier product	927,813,748	370,764,078	557,049,669	635,284,714	85,609,092	147,884,791	59,035,151	573,766,986	242,286,247	58,457,664	53,302,852
Total change	0	0	0	0	0	0	0	0	0	0	0
Baseline per AE per day	0.02	0.02	0.02	0.02	0.02	0.02	0.02	0.02	0.02	0.02	0.02
Reformulated per AE per day	0.02	0.02	0.02	0.02	0.02	0.02	0.02	0.02	0.02	0.02	0.02
Change per AE per day	0.00	0.00	0.00	0.00	0.00	0.00	0.00	0.00	0.00	0.00	0.00
Percent change per AE per day	0.00%	0.00%	0.00%	0.00%	0.00%	0.00%	0.00%	0.00%	0.00%	0.00%	0.00%
**Sugars (g)**											
Baseline (2014 average product)	28,762,226,182	11,493,686,430	17,268,539,751	19,693,826,125	2,653,881,858	4,584,428,512	1,830,089,687	17,786,776,554	7,510,873,660	1,812,187,572	1,652,388,397
Reformulated healthier product	25,978,784,938	10,381,394,195	15,597,390,743	17,787,971,984	2,397,054,582	4,140,774,140	1,652,984,234	16,065,475,597	6,784,014,919	1,636,814,581	1,492,479,842
Total change	-2,783,441,243	-1,112,292,235	-1,671,149,008	-1,905,854,141	-256,827,277	-443,654,372	-177,105,454	-1,721,300,957	-726,858,741	-175,372,991	-159,908,555
Baseline per AE per day	0.60	0.64	0.58	0.64	0.54	0.53	0.53	0.59	0.62	0.67	0.61
Reformulated per AE per day	0.55	0.58	0.53	0.58	0.49	0.48	0.48	0.53	0.56	0.61	0.55
Change per AE per day	-0.06	-0.06	-0.06	-0.06	-0.05	-0.05	-0.05	-0.06	-0.06	-0.06	-0.06
Percent change per AE per day	-10.00%	-9.38%	-10.34%	-9.38%	-9.26%	-9.43%	-9.43%	-10.17%	-9.68%	-8.96%	-9.84%
**Sodium (mg)**											
Baseline (2014 average product)	2,582,105,660,127	1,031,836,430,187	1,550,269,229,940	1,767,997,358,227	238,250,103,593	411,563,372,518	164,294,825,790	1,596,793,520,925	674,282,625,679	162,687,677,799	148,341,835,725
Reformulated healthier product	2,546,848,737,711	1,017,747,395,208	1,529,101,342,503	1,743,856,539,107	234,996,958,089	405,943,750,472	162,051,490,044	1,574,990,375,472	665,075,748,290	160,466,286,582	146,316,327,368
Total change	-35,256,922,416	-14,089,034,979	-21,167,887,437	-24,140,819,121	-3,253,145,504	-5,619,622,047	-2,243,335,746	-21,803,145,453	-9,206,877,390	-2,221,391,217	-2,025,508,357
Baseline per AE per day	54.18	57.29	52.29	57.68	48.83	47.57	47.24	53.05	55.49	60.27	54.79
Reformulated per AE per day	53.44	56.51	51.57	56.89	48.16	46.92	46.60	52.32	54.73	59.45	54.04
Change per AE per day	-0.74	-0.78	-0.71	-0.79	-0.67	-0.65	-0.65	-0.72	-0.76	-0.82	-0.75
Percent change per AE per day	-1.37%	-1.36%	-1.36%	-1.37%	-1.37%	-1.37%	-1.38%	-1.36%	-1.37%	-1.36%	-1.37%
**Dietary Fiber (g)**											
Baseline (2014 average product)	22,267,529,947	8,898,337,882	13,369,192,066	15,246,833,129	2,054,618,213	3,549,234,977	1,416,843,629	13,770,407,654	5,814,869,930	1,402,983,926	1,279,268,436
Reformulated healthier product	22,267,529,947	8,898,337,882	13,369,192,066	15,246,833,129	2,054,618,213	3,549,234,977	1,416,843,629	13,770,407,654	5,814,869,930	1,402,983,926	1,279,268,436
Total change	0	0	0	0	0	0	0	0	0	0	0
Baseline per AE per day	0.47	0.49	0.45	0.50	0.42	0.41	0.41	0.46	0.48	0.52	0.47
Reformulated per AE per day	0.47	0.49	0.45	0.50	0.42	0.41	0.41	0.46	0.48	0.52	0.47
Change per AE per day	0.00	0.00	0.00	0.00	0.00	0.00	0.00	0.00	0.00	0.00	0.00
Percent change per AE per day	0.00%	0.00%	0.00%	0.00%	0.00%	0.00%	0.00%	0.00%	0.00%	0.00%	0.00%

Potential Improvements from Reformulation of Yeast Breads by Demographic Group for Consumer Network Households with Children (Weighted), 2014

	All Households	Low Income (<185% of Poverty Line)	High Income (>185% of Poverty Line)	Non-Hispanic White	Non-Hispanic Black	Hispanic	Asian and Other	Urban	Part Urban /Part Rural	Rural	Unspecified
**2-Star Targets**											
**Calories**											
Baseline (2014 average product)	1,344,402,120,562	537,237,149,602	807,164,970,961	920,527,550,151	124,047,574,598	214,285,061,724	85,541,934,089	831,388,362,134	351,072,772,048	84,705,154,556	77,235,831,824
Reformulated healthier product	1,142,138,723,542	456,410,580,510	685,728,143,031	782,035,482,565	105,384,792,498	182,046,177,352	72,672,271,127	706,307,159,274	298,254,370,180	71,961,383,892	65,615,810,197
Total change	-202,263,397,020	-80,826,569,091	-121,436,827,929	-138,492,067,587	-18,662,782,100	-32,238,884,373	-12,869,662,962	-125,081,202,861	-52,818,401,868	-12,743,770,665	-11,620,021,627
Baseline per AE per day	28.21	29.83	27.22	30.03	25.42	24.77	24.60	27.62	28.89	31.38	28.53
Reformulated per AE per day	23.96	25.34	23.13	25.51	21.60	21.04	20.90	23.46	24.55	26.66	24.24
Change per AE per day	-4.24	-4.49	-4.10	-4.52	-3.82	-3.73	-3.70	-4.16	-4.35	-4.72	-4.29
Percent change per AE per day	-15.03%	-15.05%	-15.06%	-15.05%	-15.03%	-15.06%	-15.04%	-15.06%	-15.06%	-15.04%	-15.04%
**Saturated Fat (g)**											
Baseline (2014 average product)	927,813,748	370,764,078	557,049,669	635,284,714	85,609,092	147,884,791	59,035,151	573,766,986	242,286,247	58,457,664	53,302,852
Reformulated healthier product	927,813,748	370,764,078	557,049,669	635,284,714	85,609,092	147,884,791	59,035,151	573,766,986	242,286,247	58,457,664	53,302,852
Total change	0	0	0	0	0	0	0	0	0	0	0
Baseline per AE per day	0.02	0.02	0.02	0.02	0.02	0.02	0.02	0.02	0.02	0.02	0.02
Reformulated per AE per day	0.02	0.02	0.02	0.02	0.02	0.02	0.02	0.02	0.02	0.02	0.02
Change per AE per day	0.00	0.00	0.00	0.00	0.00	0.00	0.00	0.00	0.00	0.00	0.00
Percent change per AE per day	0.00%	0.00%	0.00%	0.00%	0.00%	0.00%	0.00%	0.00%	0.00%	0.00%	0.00%
**Sugars (g)**											
Baseline (2014 average product)	28,762,226,182	11,493,686,430	17,268,539,751	19,693,826,125	2,653,881,858	4,584,428,512	1,830,089,687	17,786,776,554	7,510,873,660	1,812,187,572	1,652,388,397
Reformulated healthier product	19,484,088,704	7,786,045,646	11,698,043,057	13,340,978,988	1,797,790,936	3,105,580,605	1,239,738,175	12,049,106,698	5,088,011,189	1,227,610,936	1,119,359,882
Total change	-9,278,137,478	-3,707,640,784	-5,570,496,694	-6,352,847,137	-856,090,922	-1,478,847,907	-590,351,512	-5,737,669,856	-2,422,862,471	-584,576,636	-533,028,515
Baseline per AE per day	0.60	0.64	0.58	0.64	0.54	0.53	0.53	0.59	0.62	0.67	0.61
Reformulated per AE per day	0.41	0.43	0.39	0.44	0.37	0.36	0.36	0.40	0.42	0.45	0.41
Change per AE per day	-0.19	-0.21	-0.19	-0.21	-0.18	-0.17	-0.17	-0.19	-0.20	-0.22	-0.20
Percent change per AE per day	-31.67%	-32.81%	-32.76%	-32.81%	-33.33%	-32.08%	-32.08%	-32.20%	-32.26%	-32.84%	-32.79%
**Sodium (mg)**											
Baseline (2014 average product)	2,582,105,660,127	1,031,836,430,187	1,550,269,229,940	1,767,997,358,227	238,250,103,593	411,563,372,518	164,294,825,790	1,596,793,520,925	674,282,625,679	162,687,677,799	148,341,835,725
Reformulated healthier product	2,076,447,167,576	829,770,007,459	1,246,677,160,117	1,421,767,189,261	191,593,148,344	330,966,161,587	132,120,668,386	1,284,090,513,773	542,236,621,010	130,828,251,137	119,291,781,657
Total change	-505,658,492,551	-202,066,422,728	-303,592,069,823	-346,230,168,967	-46,656,955,249	-80,597,210,932	-32,174,157,404	-312,703,007,152	-132,046,004,670	-31,859,426,662	-29,050,054,068
Baseline per AE per day	54.18	57.29	52.29	57.68	48.83	47.57	47.24	53.05	55.49	60.27	54.79
Reformulated per AE per day	43.57	46.07	42.05	46.39	39.27	38.25	37.99	42.66	44.62	48.47	44.06
Change per AE per day	-10.61	-11.22	-10.24	-11.30	-9.56	-9.32	-9.25	-10.39	-10.87	-11.80	-10.73
Percent change per AE per day	-19.58%	-19.58%	-19.58%	-19.59%	-19.58%	-19.59%	-19.58%	-19.59%	-19.59%	-19.58%	-19.58%
**Dietary Fiber (g)**											
Baseline (2014 average product)	22,267,529,947	8,898,337,882	13,369,192,066	15,246,833,129	2,054,618,213	3,549,234,977	1,416,843,629	13,770,407,654	5,814,869,930	1,402,983,926	1,279,268,436
Reformulated healthier product	42,679,432,399	17,055,147,606	25,624,284,792	29,223,096,830	3,938,018,241	6,802,700,372	2,715,616,955	26,393,281,338	11,145,167,367	2,689,052,526	2,451,931,169
Total change	20,411,902,452	8,156,809,725	12,255,092,727	13,976,263,701	1,883,400,028	3,253,465,395	1,298,773,326	12,622,873,683	5,330,297,436	1,286,068,599	1,172,662,733
Baseline per AE per day	0.47	0.49	0.45	0.50	0.42	0.41	0.41	0.46	0.48	0.52	0.47
Reformulated per AE per day	0.90	0.95	0.86	0.95	0.81	0.79	0.78	0.88	0.92	1.00	0.91
Change per AE per day	0.43	0.45	0.41	0.46	0.39	0.38	0.37	0.42	0.44	0.48	0.43
Percent change per AE per day	91.49%	91.84%	91.11%	92.00%	92.86%	92.68%	90.24%	91.30%	91.67%	92.31%	91.49%

Potential Improvements from Reformulation of Yeast Breads by Demographic Group for Consumer Network Households with Children (Weighted), 2014

	All Households	Low Income (<185% of Poverty Line)	High Income (>185% of Poverty Line)	Non-Hispanic White	Non-Hispanic Black	Hispanic	Asian and Other	Urban	Part Urban /Part Rural	Rural	Unspecified
**3-Star Targets**											
**Calories**											
Baseline (2014 average product)	1,344,402,120,562	537,237,149,602	807,164,970,961	920,527,550,151	124,047,574,598	214,285,061,724	85,541,934,089	831,388,362,134	351,072,772,048	84,705,154,556	77,235,831,824
Reformulated healthier product	1,195,024,107,166	477,544,132,979	717,479,974,187	818,246,711,246	110,264,510,754	190,475,610,422	76,037,274,746	739,011,877,453	312,064,686,265	75,293,470,717	68,654,072,732
Total change	-149,378,013,396	-59,693,016,622	-89,684,996,773	-102,280,838,906	-13,783,063,844	-23,809,451,303	-9,504,659,343	-92,376,484,682	-39,008,085,783	-9,411,683,840	-8,581,759,091
Baseline per AE per day	28.21	29.83	27.22	30.03	25.42	24.77	24.60	27.62	28.89	31.38	28.53
Reformulated per AE per day	25.07	26.51	24.20	26.70	22.60	22.01	21.86	24.55	25.68	27.90	25.36
Change per AE per day	-3.13	-3.31	-3.02	-3.34	-2.82	-2.75	-2.73	-3.07	-3.21	-3.49	-3.17
Percent change per AE per day	-11.10%	-11.10%	-11.09%	-11.12%	-11.09%	-11.10%	-11.10%	-11.12%	-11.11%	-11.12%	-11.11%
**Saturated Fat (g)**											
Baseline (2014 average product)	927,813,748	370,764,078	557,049,669	635,284,714	85,609,092	147,884,791	59,035,151	573,766,986	242,286,247	58,457,664	53,302,852
Reformulated healthier product	927,813,748	370,764,078	557,049,669	635,284,714	85,609,092	147,884,791	59,035,151	573,766,986	242,286,247	58,457,664	53,302,852
Total change	0	0	0	0	0	0	0	0	0	0	0
Baseline per AE per day	0.02	0.02	0.02	0.02	0.02	0.02	0.02	0.02	0.02	0.02	0.02
Reformulated per AE per day	0.02	0.02	0.02	0.02	0.02	0.02	0.02	0.02	0.02	0.02	0.02
Change per AE per day	0.00	0.00	0.00	0.00	0.00	0.00	0.00	0.00	0.00	0.00	0.00
Percent change per AE per day	0.00%	0.00%	0.00%	0.00%	0.00%	0.00%	0.00%	0.00%	0.00%	0.00%	0.00%
**Sugars (g)**											
Baseline (2014 average product)	28,762,226,182	11,493,686,430	17,268,539,751	19,693,826,125	2,653,881,858	4,584,428,512	1,830,089,687	17,786,776,554	7,510,873,660	1,812,187,572	1,652,388,397
Reformulated healthier product	17,628,461,208	7,044,517,490	10,583,943,719	12,070,409,560	1,626,572,752	2,809,811,023	1,121,667,873	10,901,572,726	4,603,438,695	1,110,695,608	1,012,754,179
Total change	-11,133,764,974	-4,449,168,941	-6,684,596,033	-7,623,416,564	-1,027,309,106	-1,774,617,488	-708,421,814	-6,885,203,827	-2,907,434,965	-701,491,963	-639,634,218
Baseline per AE per day	0.60	0.64	0.58	0.64	0.54	0.53	0.53	0.59	0.62	0.67	0.61
Reformulated per AE per day	0.37	0.39	0.36	0.39	0.33	0.32	0.32	0.36	0.38	0.41	0.37
Change per AE per day	-0.23	-0.25	-0.23	-0.25	-0.21	-0.21	-0.20	-0.23	-0.24	-0.26	-0.24
Percent change per AE per day	-38.33%	-39.06%	-39.66%	-39.06%	-38.89%	-39.62%	-37.74%	-38.98%	-38.71%	-38.81%	-39.34%
**Sodium (mg)**											
Baseline (2014 average product)	2,582,105,660,127	1,031,836,430,187	1,550,269,229,940	1,767,997,358,227	238,250,103,593	411,563,372,518	164,294,825,790	1,596,793,520,925	674,282,625,679	162,687,677,799	148,341,835,725
Reformulated healthier product	1,873,255,956,808	748,572,674,290	1,124,683,282,519	1,282,639,836,960	172,844,757,152	298,579,392,423	119,191,970,273	1,158,435,543,926	489,175,932,895	118,026,022,808	107,618,457,179
Total change	-708,849,703,319	-283,263,755,898	-425,585,947,422	-485,357,521,267	-65,405,346,441	-112,983,980,095	-45,102,855,517	-438,357,976,998	-185,106,692,784	-44,661,654,990	-40,723,378,546
Baseline per AE per day	54.18	57.29	52.29	57.68	48.83	47.57	47.24	53.05	55.49	60.27	54.79
Reformulated per AE per day	39.30	41.56	37.93	41.85	35.42	34.51	34.27	38.48	40.26	43.73	39.75
Change per AE per day	-14.87	-15.73	-14.35	-15.84	-13.40	-13.06	-12.97	-14.56	-15.23	-16.55	-15.04
Percent change per AE per day	-27.45%	-27.46%	-27.44%	-27.46%	-27.44%	-27.45%	-27.46%	-27.45%	-27.45%	-27.46%	-27.45%
**Dietary Fiber (g)**											
Baseline (2014 average product)	22,267,529,947	8,898,337,882	13,369,192,066	15,246,833,129	2,054,618,213	3,549,234,977	1,416,843,629	13,770,407,654	5,814,869,930	1,402,983,926	1,279,268,436
Reformulated healthier product	52,885,383,625	21,133,552,469	31,751,831,156	36,211,228,681	4,879,718,255	8,429,433,070	3,365,003,618	32,704,718,179	13,810,316,085	3,332,086,825	3,038,262,536
Total change	30,617,853,677	12,235,214,587	18,382,639,090	20,964,395,552	2,825,100,043	4,880,198,093	1,948,159,990	18,934,310,525	7,995,446,154	1,929,102,899	1,758,994,100
Baseline per AE per day	0.47	0.49	0.45	0.50	0.42	0.41	0.41	0.46	0.48	0.52	0.47
Reformulated per AE per day	1.11	1.17	1.07	1.18	1.00	0.97	0.97	1.09	1.14	1.23	1.12
Change per AE per day	0.64	0.68	0.62	0.68	0.58	0.56	0.56	0.63	0.66	0.71	0.65
Percent change per AE per day	136.17%	138.78%	137.78%	136.00%	138.10%	136.59%	136.59%	136.96%	137.50%	136.54%	138.30%

Potential Improvements from Reformulation of Yogurt by Demographic Group for Consumer Network Households with Children (Weighted), 2014

	All Households	Low Income (<185% of Poverty Line)	High Income (>185% of Poverty Line)	Non-Hispanic White	Non-Hispanic Black	Hispanic	Asian and Other	Urban	Part Urban /Part Rural	Rural	Unspecified
**1-Star Targets**											
**Calories**											
Baseline (2014 average product)	377,672,953,165	112,938,676,390	264,734,276,776	271,829,070,328	24,871,540,282	56,602,609,696	24,369,732,998	243,569,734,246	97,084,376,789	18,828,519,804	18,190,322,327
Reformulated healthier product	339,661,297,203	101,571,735,560	238,089,561,644	244,470,285,392	22,368,293,956	50,905,725,902	21,916,992,078	219,055,167,176	87,313,123,913	16,933,485,460	16,359,520,655
Total change	-38,011,655,962	-11,366,940,830	-26,644,715,132	-27,358,784,936	-2,503,246,326	-5,696,883,794	-2,452,740,920	-24,514,567,070	-9,771,252,876	-1,895,034,344	-1,830,801,672
Baseline per AE per day	7.92	6.27	8.93	8.87	5.10	6.54	7.01	8.09	7.99	6.98	6.72
Reformulated per AE per day	7.13	5.64	8.03	7.98	4.58	5.88	6.30	7.28	7.19	6.27	6.04
Change per AE per day	-0.80	-0.63	-0.90	-0.89	-0.51	-0.66	-0.71	-0.81	-0.80	-0.70	-0.68
Percent change per AE per day	-10.10%	-10.05%	-10.08%	-10.03%	-10.00%	-10.09%	-10.13%	-10.01%	-10.01%	-10.03%	-10.12%
**Saturated Fat (g)**											
Baseline (2014 average product)	2,715,118,283	811,924,345	1,903,193,938	1,954,198,924	178,803,309	406,920,271	175,195,780	1,751,040,505	697,946,634	135,359,596	130,771,548
Reformulated healthier product	3,801,165,596	1,136,694,083	2,664,471,513	2,735,878,494	250,324,633	569,688,379	245,274,092	2,451,456,707	977,125,288	189,503,434	183,080,167
Total change	1,086,047,313	324,769,738	761,277,575	781,679,570	71,521,324	162,768,108	70,078,312	700,416,202	279,178,654	54,143,838	52,308,619
Baseline per AE per day	0.06	0.05	0.06	0.06	0.04	0.05	0.05	0.06	0.06	0.05	0.05
Reformulated per AE per day	0.08	0.06	0.09	0.09	0.05	0.07	0.07	0.08	0.08	0.07	0.07
Change per AE per day	0.02	0.02	0.03	0.03	0.01	0.02	0.02	0.02	0.02	0.02	0.02
Percent change per AE per day	33.33%	40.00%	50.00%	50.00%	25.00%	40.00%	40.00%	33.33%	33.33%	40.00%	40.00%
**Sugars (g)**											
Baseline (2014 average product)	50,501,200,064	15,101,792,817	35,399,407,247	36,348,099,986	3,325,741,547	7,568,717,041	3,258,641,508	32,569,353,393	12,981,807,392	2,517,688,486	2,432,350,793
Reformulated healthier product	34,482,002,194	10,311,439,182	24,170,563,013	24,818,326,335	2,270,802,024	5,167,887,442	2,224,986,406	22,238,214,414	8,863,922,252	1,719,066,869	1,660,798,660
Total change	-16,019,197,870	-4,790,353,636	-11,228,844,234	-11,529,773,652	-1,054,939,523	-2,400,829,599	-1,033,655,102	-10,331,138,980	-4,117,885,141	-798,621,616	-771,552,133
Baseline per AE per day	1.06	0.84	1.19	1.19	0.68	0.87	0.94	1.08	1.07	0.93	0.90
Reformulated per AE per day	0.72	0.57	0.82	0.81	0.47	0.60	0.64	0.74	0.73	0.64	0.61
Change per AE per day	-0.34	-0.27	-0.38	-0.38	-0.22	-0.28	-0.30	-0.34	-0.34	-0.30	-0.28
Percent change per AE per day	-32.08%	-32.14%	-31.93%	-31.93%	-32.35%	-32.18%	-31.91%	-31.48%	-31.78%	-32.26%	-31.11%
**Sodium (mg)**											
Baseline (2014 average product)	249,519,370,208	74,615,847,306	174,903,522,902	179,590,881,116	16,432,024,097	37,395,972,905	16,100,492,182	160,920,622,410	64,141,295,665	12,439,546,872	12,017,905,261
Reformulated healthier product	231,056,565,883	69,094,761,760	161,961,804,124	166,302,328,432	15,216,161,596	34,628,915,062	14,909,160,878	149,013,546,976	59,395,258,553	11,519,101,620	11,128,658,735
Total change	-18,462,804,324	-5,521,085,546	-12,941,718,778	-13,288,552,683	-1,215,862,501	-2,767,057,843	-1,191,331,304	-11,907,075,434	-4,746,037,111	-920,445,253	-889,246,526
Baseline per AE per day	5.24	4.14	5.90	5.86	3.37	4.32	4.63	5.35	5.28	4.61	4.44
Reformulated per AE per day	4.85	3.84	5.46	5.43	3.12	4.00	4.29	4.95	4.89	4.27	4.11
Change per AE per day	-0.39	-0.31	-0.44	-0.43	-0.25	-0.32	-0.34	-0.40	-0.39	-0.34	-0.33
Percent change per AE per day	-7.44%	-7.49%	-7.46%	-7.34%	-7.42%	-7.41%	-7.34%	-7.48%	-7.39%	-7.38%	-7.43%
**Dietary Fiber (g)**											
Baseline (2014 average product)	1,086,047,313	324,769,738	761,277,575	781,679,570	71,521,324	162,768,108	70,078,312	700,416,202	279,178,654	54,143,838	52,308,619
Reformulated healthier product	5,973,260,223	1,786,233,559	4,187,026,664	4,299,237,633	393,367,280	895,224,596	385,430,716	3,852,289,111	1,535,482,595	297,791,111	287,697,406
Total change	4,887,212,909	1,461,463,821	3,425,749,088	3,517,558,063	321,845,956	732,456,488	315,352,404	3,151,872,909	1,256,303,941	243,647,273	235,388,786
Baseline per AE per day	0.02	0.02	0.03	0.03	0.01	0.02	0.02	0.02	0.02	0.02	0.02
Reformulated per AE per day	0.13	0.10	0.14	0.14	0.08	0.10	0.11	0.13	0.13	0.11	0.11
Change per AE per day	0.10	0.08	0.12	0.11	0.07	0.08	0.09	0.10	0.10	0.09	0.09
Percent change per AE per day	500.00%	400.00%	400.00%	366.67%	700.00%	400.00%	450.00%	500.00%	500.00%	450.00%	450.00%

Potential Improvements from Reformulation of Yogurt by Demographic Group for Consumer Network Households with Children (Weighted), 2014

	All Households	Low Income (<185% of Poverty Line)	High Income (>185% of Poverty Line)	Non-Hispanic White	Non-Hispanic Black	Hispanic	Asian and Other	Urban	Part Urban /Part Rural	Rural	Unspecified
**2-Star Targets**											
**Calories**											
Baseline (2014 average product)	377,672,953,165	112,938,676,390	264,734,276,776	271,829,070,328	24,871,540,282	56,602,609,696	24,369,732,998	243,569,734,246	97,084,376,789	18,828,519,804	18,190,322,327
Reformulated healthier product	256,307,165,915	76,645,658,168	179,661,507,747	184,476,378,426	16,879,032,370	38,413,273,582	16,538,481,632	165,298,223,672	65,886,162,250	12,777,945,862	12,344,834,131
Total change	-121,365,787,250	-36,293,018,222	-85,072,769,029	-87,352,691,903	-7,992,507,912	-18,189,336,114	-7,831,251,366	-78,271,510,574	-31,198,214,540	-6,050,573,941	-5,845,488,196
Baseline per AE per day	7.92	6.27	8.93	8.87	5.10	6.54	7.01	8.09	7.99	6.98	6.72
Reformulated per AE per day	5.38	4.26	6.06	6.02	3.46	4.44	4.76	5.49	5.42	4.73	4.56
Change per AE per day	-2.55	-2.02	-2.87	-2.85	-1.64	-2.10	-2.25	-2.60	-2.57	-2.24	-2.16
Percent change per AE per day	-32.20%	-32.22%	-32.14%	-32.13%	-32.16%	-32.11%	-32.10%	-32.14%	-32.17%	-32.09%	-32.14%
**Saturated Fat (g)**											
Baseline (2014 average product)	2,715,118,283	811,924,345	1,903,193,938	1,954,198,924	178,803,309	406,920,271	175,195,780	1,751,040,505	697,946,634	135,359,596	130,771,548
Reformulated healthier product	1,629,070,970	487,154,607	1,141,916,363	1,172,519,354	107,281,985	244,152,163	105,117,468	1,050,624,303	418,767,980	81,215,758	78,462,929
Total change	-1,086,047,313	-324,769,738	-761,277,575	-781,679,570	-71,521,324	-162,768,108	-70,078,312	-700,416,202	-279,178,654	-54,143,838	-52,308,619
Baseline per AE per day	0.06	0.05	0.06	0.06	0.04	0.05	0.05	0.06	0.06	0.05	0.05
Reformulated per AE per day	0.03	0.03	0.04	0.04	0.02	0.03	0.03	0.03	0.03	0.03	0.03
Change per AE per day	-0.02	-0.02	-0.03	-0.03	-0.01	-0.02	-0.02	-0.02	-0.02	-0.02	-0.02
Percent change per AE per day	-33.33%	-40.00%	-50.00%	-50.00%	-25.00%	-40.00%	-40.00%	-33.33%	-33.33%	-40.00%	-40.00%
**Sugars (g)**											
Baseline (2014 average product)	50,501,200,064	15,101,792,817	35,399,407,247	36,348,099,986	3,325,741,547	7,568,717,041	3,258,641,508	32,569,353,393	12,981,807,392	2,517,688,486	2,432,350,793
Reformulated healthier product	22,535,481,749	6,738,972,064	15,796,509,685	16,219,851,069	1,484,067,465	3,377,438,249	1,454,124,974	14,533,636,192	5,792,957,062	1,123,484,647	1,085,403,848
Total change	-27,965,718,315	-8,362,820,754	-19,602,897,561	-20,128,248,917	-1,841,674,083	-4,191,278,791	-1,804,516,534	-18,035,717,202	-7,188,850,330	-1,394,203,839	-1,346,946,944
Baseline per AE per day	1.06	0.84	1.19	1.19	0.68	0.87	0.94	1.08	1.07	0.93	0.90
Reformulated per AE per day	0.47	0.37	0.53	0.53	0.30	0.39	0.42	0.48	0.48	0.42	0.40
Change per AE per day	-0.59	-0.46	-0.66	-0.66	-0.38	-0.48	-0.52	-0.60	-0.59	-0.52	-0.50
Percent change per AE per day	-55.66%	-54.76%	-55.46%	-55.46%	-55.88%	-55.17%	-55.32%	-55.56%	-55.14%	-55.91%	-55.56%
**Sodium (mg)**											
Baseline (2014 average product)	249,519,370,208	74,615,847,306	174,903,522,902	179,590,881,116	16,432,024,097	37,395,972,905	16,100,492,182	160,920,622,410	64,141,295,665	12,439,546,872	12,017,905,261
Reformulated healthier product	247,347,275,581	73,966,307,830	173,380,967,752	178,027,521,976	16,288,981,450	37,070,436,688	15,960,335,558	159,519,790,006	63,582,938,357	12,331,259,196	11,913,288,023
Total change	-2,172,094,626	-649,539,476	-1,522,555,150	-1,563,359,139	-143,042,647	-325,536,217	-140,156,624	-1,400,832,404	-558,357,307	-108,287,677	-104,617,238
Baseline per AE per day	5.24	4.14	5.90	5.86	3.37	4.32	4.63	5.35	5.28	4.61	4.44
Reformulated per AE per day	5.19	4.11	5.85	5.81	3.34	4.28	4.59	5.30	5.23	4.57	4.40
Change per AE per day	-0.05	-0.04	-0.05	-0.05	-0.03	-0.04	-0.04	-0.05	-0.05	-0.04	-0.04
Percent change per AE per day	-0.95%	-0.97%	-0.85%	-0.85%	-0.89%	-0.93%	-0.86%	-0.93%	-0.95%	-0.87%	-0.90%
**Dietary Fiber (g)**											
Baseline (2014 average product)	1,086,047,313	324,769,738	761,277,575	781,679,570	71,521,324	162,768,108	70,078,312	700,416,202	279,178,654	54,143,838	52,308,619
Reformulated healthier product	2,172,094,626	649,539,476	1,522,555,150	1,563,359,139	143,042,647	325,536,217	140,156,624	1,400,832,404	558,357,307	108,287,677	104,617,238
Total change	1,086,047,313	324,769,738	761,277,575	781,679,570	71,521,324	162,768,108	70,078,312	700,416,202	279,178,654	54,143,838	52,308,619
Baseline per AE per day	0.02	0.02	0.03	0.03	0.01	0.02	0.02	0.02	0.02	0.02	0.02
Reformulated per AE per day	0.05	0.04	0.05	0.05	0.03	0.04	0.04	0.05	0.05	0.04	0.04
Change per AE per day	0.02	0.02	0.03	0.03	0.01	0.02	0.02	0.02	0.02	0.02	0.02
Percent change per AE per day	100.00%	100.00%	100.00%	100.00%	100.00%	100.00%	100.00%	100.00%	100.00%	100.00%	100.00%

Potential Improvements from Reformulation of Yogurt by Demographic Group for Consumer Network Households with Children (Weighted), 2014

	All Households	Low Income (<185% of Poverty Line)	High Income (>185% of Poverty Line)	Non-Hispanic White	Non-Hispanic Black	Hispanic	Asian and Other	Urban	Part Urban /Part Rural	Rural	Unspecified
**3-Star Targets**											
**Calories**											
Baseline (2014 average product)	377,672,953,165	112,938,676,390	264,734,276,776	271,829,070,328	24,871,540,282	56,602,609,696	24,369,732,998	243,569,734,246	97,084,376,789	18,828,519,804	18,190,322,327
Reformulated healthier product	306,536,854,151	91,666,258,551	214,870,595,600	220,629,058,520	20,186,893,586	45,941,298,596	19,779,603,562	197,692,473,015	78,798,174,979	15,282,098,388	14,764,107,769
Total change	-71,136,099,015	-21,272,417,839	-49,863,681,176	-51,200,011,809	-4,684,646,696	-10,661,311,100	-4,590,129,436	-45,877,261,231	-18,286,201,811	-3,546,421,415	-3,426,214,558
Baseline per AE per day	7.92	6.27	8.93	8.87	5.10	6.54	7.01	8.09	7.99	6.98	6.72
Reformulated per AE per day	6.43	5.09	7.25	7.20	4.14	5.31	5.69	6.57	6.48	5.66	5.45
Change per AE per day	-1.49	-1.18	-1.68	-1.67	-0.96	-1.23	-1.32	-1.52	-1.50	-1.31	-1.27
Percent change per AE per day	-18.81%	-18.82%	-18.81%	-18.83%	-18.82%	-18.81%	-18.83%	-18.79%	-18.77%	-18.77%	-18.90%
**Saturated Fat (g)**											
Baseline (2014 average product)	2,715,118,283	811,924,345	1,903,193,938	1,954,198,924	178,803,309	406,920,271	175,195,780	1,751,040,505	697,946,634	135,359,596	130,771,548
Reformulated healthier product	0	0	0	0	0	0	0	0	0	0	0
Total change	-2,715,118,283	-811,924,345	-1,903,193,938	-1,954,198,924	-178,803,309	-406,920,271	-175,195,780	-1,751,040,505	-697,946,634	-135,359,596	-130,771,548
Baseline per AE per day	0.06	0.05	0.06	0.06	0.04	0.05	0.05	0.06	0.06	0.05	0.05
Reformulated per AE per day	0.00	0.00	0.00	0.00	0.00	0.00	0.00	0.00	0.00	0.00	0.00
Change per AE per day	-0.06	-0.05	-0.06	-0.06	-0.04	-0.05	-0.05	-0.06	-0.06	-0.05	-0.05
Percent change per AE per day	-100.00%	-100.00%	-100.00%	-100.00%	-100.00%	-100.00%	-100.00%	-100.00%	-100.00%	-100.00%	-100.00%
**Sugars (g)**											
Baseline (2014 average product)	50,501,200,064	15,101,792,817	35,399,407,247	36,348,099,986	3,325,741,547	7,568,717,041	3,258,641,508	32,569,353,393	12,981,807,392	2,517,688,486	2,432,350,793
Reformulated healthier product	21,449,434,436	6,414,202,326	15,035,232,110	15,438,171,500	1,412,546,141	3,214,670,141	1,384,046,662	13,833,219,990	5,513,778,409	1,069,340,808	1,033,095,229
Total change	-29,051,765,628	-8,687,590,492	-20,364,175,137	-20,909,928,487	-1,913,195,406	-4,354,046,900	-1,874,594,846	-18,736,133,404	-7,468,028,984	-1,448,347,677	-1,399,255,564
Baseline per AE per day	1.06	0.84	1.19	1.19	0.68	0.87	0.94	1.08	1.07	0.93	0.90
Reformulated per AE per day	0.45	0.36	0.51	0.50	0.29	0.37	0.40	0.46	0.45	0.40	0.38
Change per AE per day	-0.61	-0.48	-0.69	-0.68	-0.39	-0.50	-0.54	-0.62	-0.61	-0.54	-0.52
Percent change per AE per day	-57.55%	-57.14%	-57.98%	-57.14%	-57.35%	-57.47%	-57.45%	-57.41%	-57.01%	-58.06%	-57.78%
**Sodium (mg)**											
Baseline (2014 average product)	249,519,370,208	74,615,847,306	174,903,522,902	179,590,881,116	16,432,024,097	37,395,972,905	16,100,492,182	160,920,622,410	64,141,295,665	12,439,546,872	12,017,905,261
Reformulated healthier product	235,943,778,793	70,556,225,581	165,387,553,212	169,819,886,496	15,538,007,552	35,361,371,550	15,224,513,282	152,165,419,885	60,651,562,495	11,762,748,892	11,364,047,521
Total change	-13,575,591,415	-4,059,621,725	-9,515,969,690	-9,770,994,620	-894,016,545	-2,034,601,355	-875,978,900	-8,755,202,525	-3,489,733,170	-676,797,980	-653,857,740
Baseline per AE per day	5.24	4.14	5.90	5.86	3.37	4.32	4.63	5.35	5.28	4.61	4.44
Reformulated per AE per day	4.95	3.92	5.58	5.54	3.18	4.09	4.38	5.05	4.99	4.36	4.20
Change per AE per day	-0.28	-0.23	-0.32	-0.32	-0.18	-0.24	-0.25	-0.29	-0.29	-0.25	-0.24
Percent change per AE per day	-5.34%	-5.56%	-5.42%	-5.46%	-5.34%	-5.56%	-5.40%	-5.42%	-5.49%	-5.42%	-5.41%
**Dietary Fiber (g)**											
Baseline (2014 average product)	1,086,047,313	324,769,738	761,277,575	781,679,570	71,521,324	162,768,108	70,078,312	700,416,202	279,178,654	54,143,838	52,308,619
Reformulated healthier product	12,218,032,274	3,653,659,553	8,564,372,721	8,793,895,158	804,614,891	1,831,141,220	788,381,010	7,879,682,273	3,140,759,853	609,118,182	588,471,966
Total change	11,131,984,960	3,328,889,815	7,803,095,146	8,012,215,588	733,093,567	1,668,373,111	718,302,698	7,179,266,071	2,861,581,199	554,974,344	536,163,347
Baseline per AE per day	0.02	0.02	0.03	0.03	0.01	0.02	0.02	0.02	0.02	0.02	0.02
Reformulated per AE per day	0.26	0.20	0.29	0.29	0.16	0.21	0.23	0.26	0.26	0.23	0.22
Change per AE per day	0.23	0.18	0.26	0.26	0.15	0.19	0.21	0.24	0.24	0.21	0.20
Percent change per AE per day	1150.00%	900.00%	866.67%	866.67%	1500.00%	950.00%	1050.00%	1200.00%	1200.00%	1050.00%	1000.00%
